# Synthesis and bioactivity of analogues of the marine antibiotic tropodithietic acid

**DOI:** 10.3762/bjoc.10.188

**Published:** 2014-08-06

**Authors:** Patrick Rabe, Tim A Klapschinski, Nelson L Brock, Christian A Citron, Paul D’Alvise, Lone Gram, Jeroen S Dickschat

**Affiliations:** 1 Kekulé-Institut für Organische Chemie, Rheinische Friedrich-Wilhelms-Universität Bonn, Gerhard-Domagk-Straße 1, 53121 Bonn, Germany; 2 Department of Systems Biology, Technical University of Denmark, Matematiktorvet bldg. 301, 2800 Kongens Lyngby, Denmark

**Keywords:** antibiotics, natural products, *Roseobacter*, SAR study, tropodithietic acid, tropone

## Abstract

Tropodithietic acid (TDA) is a structurally unique sulfur-containing antibiotic from the *Roseobacter* clade bacterium *Phaeobacter inhibens* DSM 17395 and a few other related species. We have synthesised several structural analogues of TDA and used them in bioactivity tests against *Staphylococcus aureus* and *Vibrio anguillarum* for a structure–activity relationship (SAR) study, revealing that the sulfur-free analogue of TDA, tropone-2-carboxylic acid, has an antibiotic activity that is even stronger than the bioactivity of the natural product. The synthesis of this compound and of several analogues is presented and the bioactivity of the synthetic compounds is discussed.

## Introduction

**Figure 1:**
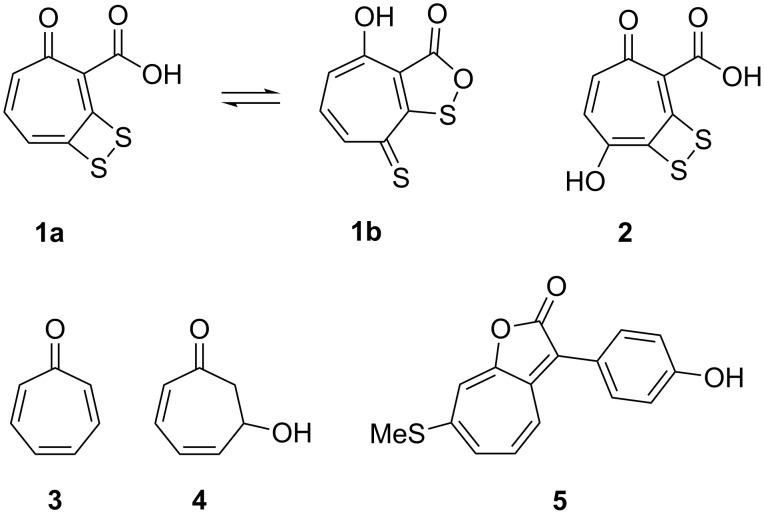
TDA and related natural products from *Phaeobacter inhibens*.

Tropodithietic acid (TDA, **1a**) is an antibiotic produced by the marine bacterium *Phaeobacter inhibens*. It has an unusual structure that is made up by a dithiet moiety fused to tropone-2-carboxylic acid (Figure 1) [1]. In *Phaeobacter* the compound is accompanied by hydroxy-TDA **2**, while its tautomer thiotropocin (**1b**) was previously reported from *Pseudomonas* [2–3]. The biosynthesis of **1** starts from phenylalanine and requires the *paaABCDE* and *paaG* genes of the upper phenylacetic acid (PAA) catabolon [4–5], the six genes *tdaABCDEF* that are located on a plasmid [6–7], and the adjacent *paaZ2* gene [8], a mutated copy of *paaZ* from the PAA catabolon. Particularly interesting is the mechanism of sulfur introduction that we have recently investigated in a study combining gene knockouts and feeding experiments with ^34^S-labelled amino acids [8]. In this study we could show that *S*-thiocysteine is the direct sulfur precursor of TDA. The introduction of sulfur proceeds via nucleophilic attack of *S*-thiocysteine to the Michael acceptor of tropone-2-carboxylic acid coenzyme A ester and oxidative elimination of cysteine. The second sulfur atom is introduced by analogous attack to the vinylogous Michael acceptor. The volatiles tropone (**3**) and tropone hydrate **4** can also be detected in *P. inhibens* headspace extracts and are shunt products of the TDA biosynthetic pathway [9]. Clardy and coworkers have recently reported on a series of structurally related compounds with algicidal activity, represented by roseobacticide A (**5**), but their biosynthesis is unknown [10].

TDA exhibits a strong antibiotic activity against a broad spectrum of bacteria including alpha- and gammaproteobacteria, flavobacteria and actinobacteria [11], but not against the eukaryotic model organisms *Artemia* sp. and *Caenorhabditis elegans*, suggesting *P. inhibens* or other TDA-producing bacteria from the *Roseobacter* clade as promising candidates to be used as probiotics in aquacultures [12]. The mode of action of TDA is unknown, but it is difficult to select resistant and tolerant strains from long-term exposures to sub inhibitory concentrations of TDA, suggesting that TDA may interact with several targets [13]. Here we report on the synthesis and bioactivity of several TDA analogues for investigating the structure–activity relationship (SAR) of this unique marine antibiotic.

## Results and Discussion

### Synthesis of analogues of tropodithietic acid

**Scheme 1:**
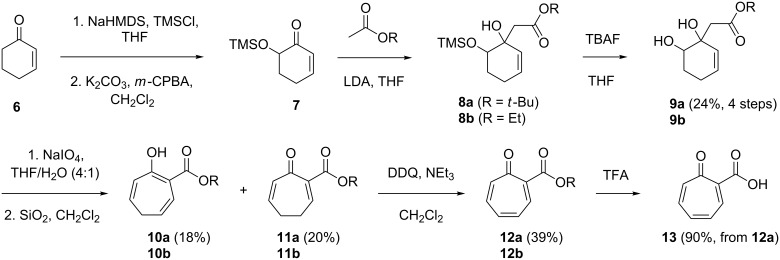
Synthesis of tropone-2-carboxylic acid (**13**).

For a detailed SAR study we first aimed at a series of compounds with a seven-membered carbocyclic core and an overall simplified structure as compared to the antibiotic TDA. Therefore, tropone-2-carboxylic acid (**13**), lacking the unprecedented dithiet moiety of TDA, was synthesised according to Scheme 1. Cyclohexenone **6** was first converted with NaHMDS and TMSCl into the corresponding silylenol ether that upon oxidation with *m*-CPBA under migration of the TMS group yielded the protected hydroxy ketone **7**. Aldol reaction with *tert*-butyl acetate to **8a** and deprotection with TBAF gave **9a** in 24% yield over four steps. Oxidative cleavage of the glycol with NaIO_4_ resulted in a β-ketoester aldehyde that upon treatment with silica gel underwent an intramolecular aldol condensation to a mixture of **10a** and **11a** that were separable by column chromatography. Oxidation with DDQ gave *tert*-butyl tropone-2-carboxylate (**12a**) that was efficiently converted into **13** by stirring in TFA. The synthesis of **12a** followed a previously published route for ethyl tropone-2-carboxylate (**12b**) [14], but saponification of this ester led to decomposition, and therefore, the *tert*-butyl ester **12a** was prepared that allowed for a conversion into **13** under acidic conditions.

**Scheme 2:**
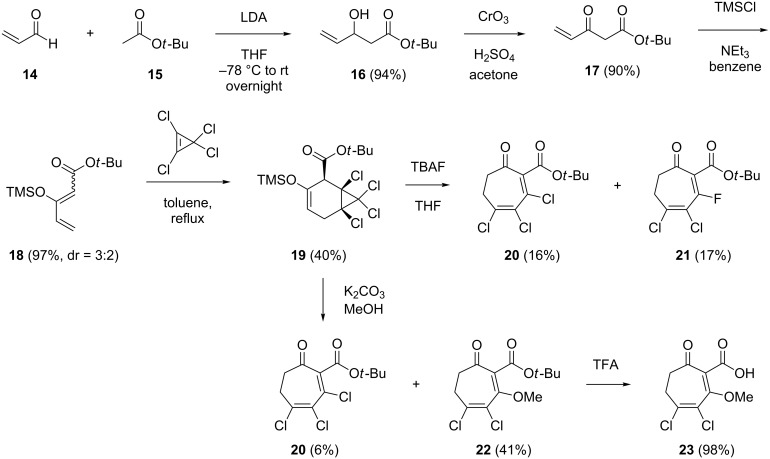
Synthesis of halogenated TDA analogues.

Furthermore, a series of TDA analogues all with a seven-membered carbocyclic core bearing a carbonyl function, a carboxylic acid or ester function in 2-position, and various functional groups such as halogen atoms and methoxy groups was synthesised (Scheme 2). Acrolein (**14**) was reacted with *tert*-butyl acetate (**15**) and LDA to yield the aldol product **16** that was subsequently oxidised to the β-keto ester **17** in a Jones oxidation. Treatment of the latter with TMSCl, imidazole and DMAP resulted in **18** as a 3:2 mixture of two diastereomers that proved to be unstable. However, the crude product was sufficiently pure for its direct usage in a Diels–Alder reaction with tetrachlorocyclopropene, resulting in the adduct **19** as a single stereoisomer. The formation of only one stereoisomer is explainable by an *E*/*Z* isomerisation of **18** and a Diels–Alder reaction that only proceeds from (*Z*)-**18**, but not from (*E*)-**18**. Treatment of **19** with TBAF resulted in cleavage of the TMS protecting group followed by instantaneous elimination of HCl under cyclopropane ring opening to **20** with 16% yield. Furthermore, similar amounts of the fluorinated derivative **21** (17%) were isolated. Its formation is explained by the nucleophilic attack of fluoride to the Michael acceptor in **20** followed by elimination of chloride. In contrast, treatment of **19** with potassium carbonate in MeOH yielded only minor amounts of **20** and the substitution product, methoxy derivative **22**, as main product. All three compounds **20**–**22** were treated under various oxidation conditions (including DDQ, IBX, SeO_2_, and MnO_2_) to install the tropone moiety by dehydrogenation, but unfortunately all these reaction conditions failed. Compound **22** was subsequently converted into the free acid **23** by treatment with TFA, while similar conversions of **20** and **21** were unsuccessful due to the instability of the products.

**Scheme 3:**
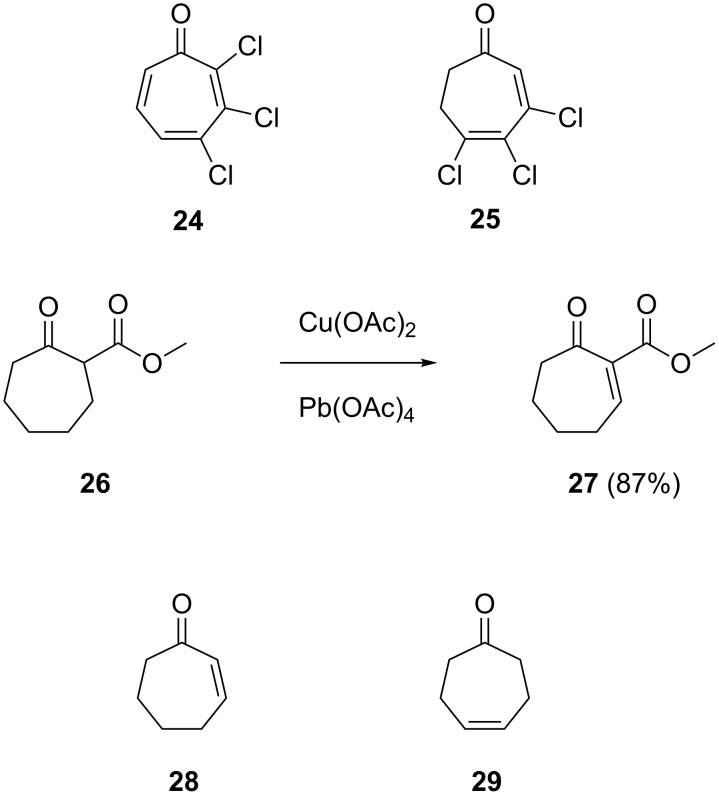
Further compounds included in this SAR study.

Two structurally related halogenated compounds lacking the carboxylic acid function as in TDA, 2,3,4-trichlorotropone (**24**) and 3,4,5-trichloro-6,7-dihydrotropone (**25**), were prepared through a known route (Scheme 3) [15]. Finally, a few cycloheptanone derivatives with rigorously simplified structures as compared to TDA were included in this study. The β-ketoester **27** containing a Michael acceptor was synthesised from methyl cycloheptanone-2-carboxylate (**26**) by oxidation with Cu(OAc)_2_ and Pb(OAc)_2_ according to a known procedure [16]. The compound cyclohept-2-en-1-one (**28**) is commercially available, while we have previously reported the synthesis of cyclohept-4-en-1-one (**29**) that was identified in headspace extracts from streptomycetes [17].

### Bioactivity tests

All synthetic compounds were screened in bioactivity tests towards two bacterial strains, the Gram-positive *Staphylococcus aureus* 8325 and the Gram-negative *Vibrio anguillarum* 90-11-287 (Table 1). These organisms were selected for our bioactivity tests, because *S. aureus* is a clinically important human pathogen and strains with resistances against multiple antibiotic drugs cause severe problems with nosocomial infections. The bioactivity against *V. anguillarum* is interesting from an ecological point of view, because *P. inhibens* was originally isolated from scallop rearings [18–20], suggesting that the natural function of *P. inhibens* may be to prevent molluscs from *Vibrio* infections.

**Table 1:** Bioactivity tests with TDA and TDA analogues.

Compound^a^	*Staphylococcus aureus* 8325		*Vibrio anguillarum* 90-11-287	
	MIC/mg L^−1^	MIC/μM	MIC/mg L^−1^	MIC/μM

**1**	7.8	39	3.9	19
**10a**	inactive		125	600
**10b**	125	700	125	700
**11a**	inactive		125	600
**12a**	125	600	125	600
**12b**	125	700	125	700
**13**	3.9	26	1.0	6.5
**20**	inactive		inactive	
**21**	125	430	125	430
**22**	inactive		inactive	
**23**	inactive		inactive	
**24**	inactive		125	720
**25**	7.8	45	15.6	89
**26**	inactive		inactive	
**27**	62.5	370	46.9	280
**28**	62.5	570	31.3	280
**29**	62.5	570	31.3	570

^a^MICs were determined using the microdilution method according to guidelines of the Clinical and Laboratory Standards Institute [21]. Compounds with MICs > 125 mg L^−1^ were regarded as inactive.

Most of the compounds tested had minimal inhibitory concentrations (MICs) exceeding that of TDA, however, two compounds were of equal or even better antibiotic activity. Compound **25** was as effective, and tropone-2-carboxylic acid (**13**), closely resembling the structure of TDA, but lacking its sulfur atoms, was even more effective with a MIC corresponding to half the MIC of TDA (MIC against *S. aureus* was 26 μM versus 39 μM, while the MIC against *V. anguillarum* was 6.5 μM versus 19 μM). This indicates that the dithiet moiety of TDA is not essential for the direct antibacterial effect of the compound, and may have other physiological or ecological functions. This result is particularly surprising, because the coenzyme A ester of **13** is an intermediate along the biosynthetic pathway to TDA, so the question arises what may have been the evolutionary advantages of extending the biosynthetic pathway from the coenzyme A ester of **13** to TDA by the introduction of two sulfur atoms. A possible answer may be that TDA has more or other targets than **13** which may have the consequence that the development of resistances against TDA is prevented, thus offering an evolutionary advantage.

## Conclusion

We have synthesised a series of compounds with seven-membered carbocyclic rings. Their structures were inspired by the antibiotic TDA from the marine bacterium *P. inhibens*. One compound, 3,4,5-trichloro-5,6-dihydrotropone (**25**), showed a strong antibiotic activity against *S. aureus* and *V. anguillarum* that is similar to the activity of TDA, while tropone-2-carboxylic acid (**13**) had an even stronger antibiotic effect. This suggests that the sulfur atoms in TDA are not essential for bioactivity, but different modes of action for TDA and its sulfur-free analogue cannot be excluded.

## Experimental

**Bioactivity testing.** All synthetic compounds were used in bioactivity tests towards two bacterial strains, *Staphylococcus aureus* 8325 [22] and *Vibrio anguillarum* 90-11-287 [23] (Table 1). The two strains were grown as precultures overnight with agitation at 25 °C in 20 mL cation-adjusted Mueller Hinton II Broth (Cat. No. 297963, Becton, Dickinson and Company, Sparks, MD, USA). The minimum inhibitory concentration (MIC) of the compounds was determined using the microdilution method according to the guidelines of the Clinical and Laboratory Standards Institute (CLSI 2006) [21]. All compounds were dissolved in DMSO to stock solutions of 1.5 g L^−1^ and further diluted in the bacterial growth medium, Mueller Hinton II Broth. Final concentrations ranging from 500 to 0.5 mg L^−1^ were tested and solvent controls were included.

## Supporting Information

File 1 Synthetic procedures, compound characterisation data and copies of NMR spectra.
