# Supplementary material for: Synthesis and bioactivity of analogues of the marine antibiotic tropodithietic acid
Source: Beilstein J Org Chem. 2014 Aug 6;10:1796–801. doi: 10.3762/bjoc.10.188 (PMC4142847; doi:10.3762/bjoc.10.188)
Supplement: File 1 — Synthetic procedures, compound characterisation data and copies of NMR spectra. [file Beilstein_J_Org_Chem-10-1796-s001.pdf]

Supporting Information

for

Synthesis and bioactivity of analogues of the marine antibiotic tropodithietic acid

Patrick Rabe[1], Tim A. Klapschinski[1], Nelson L. Brock[1], Christian A. Citron[1], Paul D'Alvise[2], Lone Gram[2] and Jeroen S. Dickschat*[1]

Address: [1]Kekulé-Institut für Organische Chemie, Rheinische Friedrich-Wilhelms-Universität Bonn, Gerhard-Domagk-Straße 1, 53121 Bonn, Germany and [2]Department of Systems Biology, Technical University of Denmark, Matematiktorvet bldg. 301, 2800 Kongens Lyngby, Denmark

Email: Jeroen S. Dickschat - dickschat@uni-bonn.de

*Corresponding author

Synthetic procedures, compound characterisation data and copies of NMR spectra

GENERAL METHODS

Chemicals were purchased from Acros Organics (Geel, Belgium) or Sigma Aldrich Chemie GmbH (Steinheim, Germany) and used without further purification. All non-aqueous reactions were performed under an inert atmosphere (N_2) in flame-dried flasks. Solvents were purified by distillation and dried according to standard methods. Thin-layer chromatography was performed with 0.2 mm precoated plastic sheets Polygram® Sil G/UV254 (Machery-Nagel). Column chromatography was carried out using Merck silica gel 60 (70-200 mesh). ^{1}H-NMR and ^{13}C-NMR spectra were recorded on Bruker DRX-400 (400 MHz), AV III-400 (400 MHz) or AV II-600 (600 MHz) spectrometers, and were referenced against TMS (δ = 0.00 ppm) for ^{1}H-NMR and $CDCl_3$ (δ = 77.01 ppm) or C_6D_6 (δ = 128.1 ppm) for ^{13}C-NMR. UV spectra were obtained using a Varian Cary 100 Bio, and IR spectra were recorded with a Bruker Tensor 27 ATR (attenuated total reflectance). GC-MS analyses were carried out with an HP6890 gas chromatograph connected to an HP5973 mass selective detector fitted with a BPX-5 fused silica capillary column (25 m, 0.25 mm i. d., 0.25 µm film). Instrumental parameters were (1) inlet pressure, 77.1 kPa, He 23.3 mL min^{-1}, (2) injection volume, 2 µL, (3) transfer line, 300°C, and (4) electron energy 70 eV. The GC was programmed as follows: 5 min at 50°C increasing at 10°C min^{-1} to 320°C, and operated in split mode (20:1, 60 s valve time). The carrier gas was He at 1 mL min^{-1}. Retention indices (*I*) were determined from a homologous series of *n*-alkanes (C_8-C_{38}). Polar compounds with free hydroxy or carboxylic acid functions were derivatised with *N*-methyl-*N*-trimethylsilyltrifluoroacetamide (MSTFA) prior to analysis.

SYNTHESIS OF COMPOUNDS

tert-Butyl 2-(1,6-dihydroxycyclohex-2-en-1-yl)acetate (**9a**): Following the procedure of Do et al. [1], a stirred solution of 2-cyclohexen-1-one (**6**) (5.77 g, 60 mmol, 1.0 eq.) in dry THF (100 mL) was treated with TMS-Cl (7.82 g, 72 mmol, 1.2 eq.). The mixture was stirred for 20 min at 0°C and then a solution of NaHMDS (2 M in THF, 34.5 mL, 69 mmol, 1.15 eq.) was added. The mixture was stirred for 2 h at 0°C, followed by dilution with CH_2Cl_2. The organic layer was washed with H_2O and dried over K_2CO_3. The mixture was concentrated under reduced pressure and the residue taken up with 200 mL of CH_2Cl_2 and cooled to 0°C. Slowly *m*CPBA (15.5 g, 90 mmol, 2.5 eq.) was added in portions and stirring was continued for 2 h at 0°C and over night at room temperature. Hexane was added and the precipitate filtered off. Evaporation of the solvent gave **7** which was used in the next step without further purification.

HN*i*Pr_2 (1.34 g, 13.3 mmol, 1.6 eq.) was dissolved in THF (14 mL) and cooled to 0°C. A 1.6 M solution of *n*BuLi (8.3 mL, 13.3 mmol, 1.6 eq.) was added and stirred for 30 min at 0°C. The mixture was cooled to -78°C and *tert*-butyl acetate (1.36 g, 8.32 mmol, 1.0 eq.) was added dropwise. Stirring was continued for 40 min at -78°C and then **7** (1.53 g, 8.3 mmol, 1.0 eq.) was added dropwise. The reaction was stirred for 2 h at -78°C and was then quenched by the addition of a sat. solution of $NaHCO_3$. The aqueous phase was extracted three times with ethyl acetate, dried over K_2CO_3 and concentrated under reduced pressure to yield crude **8a**. The residue was taken up with THF (20 mL) and cooled to 0°C. A solution of TBAF (1 M in THF, 7.3 mL, 7.3 mmol, 1.1 eq.) was added and stirring was continued for 1 h at 0°C. The solution was diluted with ethyl acetate and washed with H_2O. The organic layer was dried over $MgSO_4$ and concentrated under reduced pressure. Column chromatography on silica gel with hexane/ethyl acetate (2:1) yielded **9a** (1.13 g, 4.94 mmol, 24% over 4 steps)

as colorless oil. TLC (hexane/ethyl acetate 2:1) R_F = 0.24. ^{1}H NMR (400 MHz, $CDCl_3$) δ = 5.83 (ddd, $^3J_{H,H}$ = 9.7 Hz, $^3J_{H,H}$ = 4.5 Hz, $^3J_{H,H}$ = 2.8 Hz, 1H, CH), 5.65 (ddd, $^3J_{H,H}$ = 9.9 Hz, $^4J_{H,H}$ = 2.5 Hz, $^4J_{H,H}$ = 1.7 Hz, 1H, CH), 4.23 (br s, 1H, OH), 3.58 (dd, $^3J_{H,H}$ = 9.6 Hz, $^3J_{H,H}$ = 4.0 Hz, 1H, CH), 2.84 (br s, 1H, OH), 2.70 (d, $^2J_{H,H}$ = 15.5 Hz, 1H, CH_2), 2.42 (d, $^2J_{H,H}$ = 15.5 Hz, 1H, CH_2), 2.24 – 2.17 (m, 1H, CH_2), 2.10 – 2.00 (m, 1H, CH_2), 1.87 – 1.74 (m, 2H, CH_2), 1.48 (s, $^1J_{C,H}$ = 127.0 Hz, 9H, 3x CH_3) ppm. ^{13}C NMR (100 MHz, $CDCl_3$) δ = 172.5 (C_q), 130.4 (CH), 129.3 (CH), 81.9 (C_q), 72.0 (C_q), 69.8 (CH_2), 43.6 (CH_2), 28.1 (3x CH_3), 26.4 (CH_2), 23.9 (CH_2) ppm. IR (ATR) $\tilde{\nu}$ = 3397 (br), 3338 (br), 2982 (w), 2936 (w), 2913 (w), 2838 (w), 1725 (s), 1362 (m), 1345 (s), 1215 (m), 1149 (s), 1133 (s), 1056 (s), 1013 (m), 963 (m), 781 (m) cm^{-1}. MS (EI, 70 eV) *m/z* (%) = 228 (<1) $[M]^+$, 184 (2), 172 (6), 154 (21), 137 (36), 128 (100), 110 (55), 95 (30), 83 (22), 68 (45), 57 (94), 41 (48). GC (BPX-5) *I* = 1585. HR-MS (ESI+, MSTFA) calc. for $C_{18}H_{36}O_4Si_2Na^+$: *m/z* = 395.20444; found: *m/z* = 395.20465.

tert-Butyl 2-hydroxycyclohepta-1,3,6-triene-1-carboxylate (**10a**) and *tert*-butyl 7-oxocyclohepta-1,5-diene-1-carboxylate (**11a**): Compound **9a** (3.3 g, 14.5 mmol, 1.0 eq.) was dissolved in THF/H_2O (4:1, 280 mL). $NaIO_4$ (3.7 g, 17.4 mmol, 1.2 eq.) was added and the reaction was stirred for 2 h at room temperature. The mixture was filtered and concentrated under reduced pressure. The residue was taken up with CH_2Cl_2 (60 mL) and treated with silica gel (29 g). The reaction mixture was stirred over night at room temperature, filtered again and concentrated under reduced pressure. Column chromatography on silica gel with hexane/ethyl acetate (gradient from 10:1 to 3:1) yielded **10a** (543 mg, 2.61 mmol, 18%) and **11a** (602 mg, 2.89 mmol, 20%) as colorless oils.

Analytical data for **10a**: TLC (hexane/ethyl acetate 10:1) R_F = 0.31. ^{1}H NMR (400 MHz, $CDCl_3$) δ = 12.9 (s, 1H, OH), 6.39 (d, $^3J_{H,H}$ = 9.8 Hz, 1H, CH), 6.13 (d, $^3J_{H,H}$ = 10.2 Hz, 1H, CH), 5.97 (dt, $^3J_{H,H}$ = 10.1 Hz, $^3J_{H,H}$ = 7.1 Hz, 1H, CH), 5.30 (dt, $^3J_{H,H}$ = 9.8 Hz, $^3J_{H,H}$ = 6.7 Hz, 1H, CH), 2.37 (t, $^3J_{H,H}$ = 6.9 Hz, $^1J_{C,H}$ = 131.5 Hz, 2H, CH_2), 1.56 (s, $^1J_{C,H}$ = 127.0 Hz, 9H, 3x CH_3) ppm. ^{13}C NMR (100 MHz, $CDCl_3$) δ = 172.3 (C_q), 169.9 (C_q), 133.6 (CH), 124.8 (CH), 123.9 (CH), 117.5 (CH), 106.7 (C_q), 82.2 (C_q), 28.2 (3x CH_3), 27.1 (CH_2) ppm. IR (ATR) $\tilde{\nu}$ = 3040 (w), 2977 (w), 2933 (w), 2884 (w), 2840 (w), 1647 (m), 1547 (m), 1397 (m), 1321 (s), 1233 (s), 1144 (s), 1055 (w), 838 (m), 736 (m) cm^{-1}. UV-Vis (CH_2Cl_2) λ_{max} (log ε) = 313 (3.69), 264 (3.58), 229 (3.89) nm. MS (EI, 70 eV) *m*/*z* (%) = 208 (3) $[M]^+$, 152 (28), 134 (100), 106 (54), 77 (64), 57 (35), 41 (39). GC (BPX-5) *I* = 1475. HR-MS (EI+) calc. for $C_{12}H_{16}O_3^+$: *m*/*z* = 208.10940; found: *m*/*z* = 208.11103.

Analytical data for **11a**: TLC (hexane/ethyl acetate 3:1) R_F = 0.37. ^{1}H NMR (400 MHz, C_6D_6) δ = 6.86 (t, $^3J_{H,H}$ = 6.7 Hz, 1H, CH), 6.02 (dt, $^3J_{H,H}$ = 12.1 Hz, $^3J_{H,H}$ = 1.7 Hz, 1H, CH), 5.83 (dt, $^3J_{H,H}$ = 12.1 Hz, $^3J_{H,H}$ = 5.2 Hz, 1H, CH), 1.69 – 1.64 (m, 2H, CH_2), 1.56 – 1.52 (m, 2H, CH_2), 1.44 (s, 9H, 3x CH_3) ppm. ^{13}C NMR (100 MHz, C_6D_6) δ = 190.5 (C_q), 165.9 (C_q), 144.3 (CH), 143.2 (CH), 141.9 (C_q), 133.0 (CH), 128.6 (CH), 81.3 (C_q), 28.4 (3x CH_3), 26.8 (CH_2), 26.1 (CH_2) ppm. IR (ATR) $\tilde{\nu}$ = 3040 (w), 2978 (w), 2934 (w), 2839 (w), 1646 (m), 1547 (m), 1397 (m), 1321 (m), 1234 (s), 1144 (s), 838 (m), 710 (m) cm^{-1}. UV-Vis (CH_2Cl_2) λ_{max} (log ε) = 313 (3.68), 265 (3.58), 228 (3.89) nm. MS (EI, 70 eV) *m*/*z* (%) = 208 (<1) $[M]^+$, 153 (67), 135 (100), 107 (45), 93 (27), 79 (92), 57 (98), 39 (71). GC (BPX-5) *I* = 1611. HR-MS (EI+) calc. for $C_{12}H_{16}O_3^+$: *m*/*z* = 208.10940; found: *m*/*z* = 208.11359.

tert-Butyl 7-oxocyclohepta-1,3,5-triene-1-carboxylate (**12a**): Compounds **10a** and **11a** (1.03 g, 4.95 mmol, 1.0 eq.) were dissolved in CH_2Cl_2 (25 mL). NEt_3 (550 mg, 5.45 mmol, 1.1 eq.) was added and stirring was continued for 30 min at room temperature. DDQ (1.1 g, 4.95 mmol, 1.0 eq.) was added, followed by stirring for another 15 min. The solvent was removed under reduced pressure and the residue was purified by column chromatography on silica gel with hexane/ethyl acetate (2:1) to yield **12a** (368 mg, 1.79 mmol, 39%) as colorless oil. TLC (hexane/ethyl acetate 2:1) R_F = 0.28. ^{1}H NMR (400 MHz, $CDCl_3$) δ = 7.39 (dd, $^3J_{H,H}$ = 8.0 Hz, $^4J_{H,H}$ = 1.4 Hz, 1H, CH), 7.14 – 6.95 (m, 4H, 4x CH), 1.57 (s, $^1J_{C,H}$ = 127.0 Hz, 9H, 3x CH_3) ppm. ^{13}C NMR (100 MHz, $CDCl_3$) δ = 184.5 (C_q), 166.6 (C_q), 145.2 (C_q), 142.9 (CH), 135.9 (CH), 135.0 (CH), 134.5 (CH), 133.1 (CH), 82.7 (C_q), 28.0 (3x CH_3) ppm. IR (ATR) $\tilde{\nu}$ = 2979 (w), 2934 (w), 1719 (s), 1632 (m), 1584 (s), 1461 (w), 1294 (s), 1240 (m), 1155 (s), 1070 (m), 839 (m), 785 (m) cm^{-1}. UV-Vis (CH_2Cl_2) λ_{max} (log ε) = 313 (3.75), 304 (3.77), 231 (4.18) nm. MS (EI, 70 eV) *m*/*z* (%) = 206 (6) $[M]^+$, 151 (33), 133 (61), 105 (100), 77 (77), 51 (66), 41 (41). GC (BPX-5) *I* = 1675. HR-MS (EI+) calc. for $C_{12}H_{14}O_3^+$: *m*/*z* = 206.09375; found: *m*/*z* = 208.09697.

7-Oxocyclohepta-1,3,5-triene-1-carboxylic acid (**13**): Compound **12a** (103 mg, 0.5 mmol, 1.0 eq.) was stirred in TFA (1 mL) at room temperature for 2 h. Co-evaporation of all volatile constituents with toluene gave analytically pure **13** (67 mg, 0.45 mmol, 90%) as colorless solid. TLC (hexane/ethyl acetate 1:1) R_F = 0.23. ^{1}H NMR (400 MHz, $CDCl_3$) δ = 15.2 (br s, 1H, OH), 8.91 (d, $^3J_{H,H}$ = 8.1 Hz, 1H, CH), 7.55 – 7.44 (m, 4H, 4x CH) ppm. ^{13}C NMR (100 MHz, $CDCl_3$) δ = 189.1 (C_q), 166.1 (C_q), 146.0 (CH), 144.6 (CH), 140.9 (CH), 139.1 (CH), 135.1 (CH), 134.8 (C_q) ppm. IR (ATR) $\tilde{\nu}$ = 3042 (w), 2513 (w), 2371 (w), 1710 (m), 1617 (w), 1427 (s), 1284 (w), 1229 (m), 986 (m),

877 (m), 787 (m), 631 (s), 572 (s) cm^{-1}. UV-Vis (CH_2Cl_2) λ_{max} (log ε) = 355 (3.66), 316 (3.76), 234 (4.23) nm. MS (EI, 70 eV, MSTFA) *m*/*z* (%) = 222 (3) $[M]^+$, 207 (100), 179 (37), 163 (53), 135 (34), 105 (43), 89 (18), 77 (50), 51 (31). GC (BPX-5, MSTFA) *I* = 1660. HR-MS (ESI+) calc. for $C_8H_6O_3Na^+$: *m*/*z* = 173.02091; found: *m*/*z* = 173.02096.

tert-Butyl 3-hydroxypent-4-enoate (**16**): To a cooled (0°C) solution of diisopropylamine (11.0 g, 110.0 mmol, 1.1 eq., 0.2 M in abs. THF) was added dropwise *n*-BuLi (1.6 M in hexane, 68.8 mL, 110.0 mmol, 1.1 eq.). The mixture was stirred for 60 min at 0°C and then cooled to -78°C. *tert*-Butyl acetate (11.6 g, 100.0 mmol, 1.0 eq.) was added and the reaction mixture was stirred for 2 h at -78°C, followed by the dropwise addition of freshly distilled acrolein (5.6 g, 100.0 mmol, 1.0 eq.). Stirring was continued for 3 h at room temperature. The reaction was hydrolyzed by addition of distilled water, followed by extraction with ethyl acetate. The combined organic layers were dried over $MgSO_4$ and concentrated under reduced pressure. Column chromatography on silica gel with hexane/ethyl acetate (5:1) yielded the β-hydroxyester **16** (16.2 g, 84 mmol, 94%) as a colorless oil. TLC (hexane/ethyl acetate 5:1) R_F = 0.25. ^{1}H NMR (400 MHz, $CDCl_3$) δ = 5.88 (ddd, $^3J_{H,H}$ = 5.5 Hz, $^3J_{H,H}$ = 10.5 Hz, $^3J_{H,H}$ = 17.2 Hz, 1H, CH), 5.30 (ddd, $^2J_{H,H}$ = 1.4 Hz, $^3J_{H,H}$ = 17.2 Hz, $^4J_{H,H}$ = 1.5 Hz, 1H, CH_2), 5.14 (ddd, $^2J_{H,H}$ = 1.4 Hz, $^3J_{H,H}$ = 10.5 Hz, $^4J_{H,H}$ = 1.4 Hz, 1H, CH_2), 4.46 – 4.52 (m, 1H, CH), 3.18 (br s, 1H, OH), 2.51 (dd, $^2J_{H,H}$ = 16.1 Hz, $^3J_{H,H}$ = 4.2 Hz, 1H, CH_2), 2.43 (dd, $^2J_{H,H}$ = 16.1 Hz, $^3J_{H,H}$ = 8.2 Hz, 1H, CH_2), 1.46 (s, $^1J_{C,H}$ = 126.9 Hz, 9H, 3x CH_3) ppm. ^{13}C NMR (100 MHz, $CDCl_3$) δ = 171.6 (C_q), 138.9 (CH), 115.1 (CH_2), 81.4 (C_q), 69.0 (CH), 42.1 (CH_2), 28.1 (3x CH_3) ppm. IR (ATR) $\tilde{\nu}$ = 3434 (br), 2980 (w), 2934 (w), 1710 (s), 1477 (w), 1425 (w), 1393 (w),

1368 (m), 1285 (w), 1255 (m), 1152 (s), 1038 (w), 993 (w), 953 (w), 923 (m), 842 (m) cm^{-1}. λ_{max} (log ε) = 269 (1.64), 229 (1.92) nm. MS (EI, 70 eV) *m*/*z* (%) = 172 (<1) $[M]^+$, 116 (26), 98 (28), 81 (4), 70 (8), 57 (100), 41 (46). GC (BPX-5) *I* = 1118.

tert-Butyl 3-oxopent-4-enoate (**17**): Jones' reagent (CrO_3, 8.98 g, 89.8 mmol, 1.5 eq., dissolved in 67 mL H_2O and 8 mL H_2SO_4) was added dropwise to a solution of β-hydroxyester **16** (10.5 g, 61 mmol, 1.0 eq.) in acetone (200 mL) at 0°C. The reaction mixture was stirred for 3 h at room temperature. Methanol was added to quench excess Jones' reagent, followed by extraction with ethyl acetate. The combined organic layers were dried over $MgSO_4$ and concentrated under reduced pressure. Column chromatography on silica gel with hexane/ethyl acetate (10:1) yielded the β-ketoester **17** (9.34 g, 54.9 mmol, 90%) as orange oil. TLC (hexane/ethyl acetate 10:1) R_F = 0.45. ^{1}H NMR (400 MHz, $CDCl_3$) δ = 11.94 (s, 1H, OH, enol form), 6.41 (dd, $^3J_{H,H}$ = 10.5 Hz, $^3J_{H,H}$ = 17.6 Hz, 1H, CH, keto form), 6.26 (d, $^3J_{H,H}$ = 17.6 Hz, 1H, CH_2, keto form), 6.06 (m, 2H, enol form), 5.94 (d, $^3J_{H,H}$ = 10.5 Hz, 1H, CH_2, keto form), 5.50 (m, 1H, enol form), 4.99 (s, 1H, enol form), 3.54 (s, 2H, CH_2, keto form), 1.50 (s, 9H, 3x CH_3, enol form), 1.46 (s, 9H, 3x CH_3, keto form) ppm. ^{13}C NMR (100 MHz, $CDCl_3$) δ = 193.0 (C_q, keto form), 172.6 (C_q), 168.1 (C_q), 166.3 (C_q), 135.9 (CH, keto form), 131.4 (CH, enol form), 129.7 (CH_2, keto form), 121.9 (CH_2, enol form), 93.4 (CH, enol form), 82.0 (C_q, keto form), 81.0 (C_q, enol form), 47.8 (CH_2, keto form), 28.2 (3x CH_3, enol form), 27.9 (3x CH_3, keto form) ppm. IR (ATR) $\tilde{\nu}$ = 3430 (br), 2965 (m), 1713 (s), 1651 (m), 1588 (w), 1457 (w), 1415 (w), 1395 (w), 1369 (m), 1321 (w), 1256 (s), 1144 (s), 1085 (s), 1014 (s), 800 (s), 686 (w) cm^{-1}. λ_{max} (log ε) = 259 (3.15), 228 (3.06) nm. MS (EI, 70 eV) *m*/*z* (%) = 170 (1) $[M]^+$, 152 (1), 115 (10), 97 (38), 69 (40), 57 (100), 41 (84). GC (BPX-5) *I* = 1110.

tert-Butyl 3-((trimethylsilyl)oxy)penta-2,4-dienoate (**18**): To a solution of the β-ketoester **17** (9.3 g, 55.0 mmol, 1.0 eq.) in dry benzene (150 mL) was added triethylamine (8.3 g, 82.5 mmol, 1.5 eq.) and trimethylsilyl chloride (8.9 g, 82.5 mmol, 1.5 eq.). After stirring for 24 h the reaction was hydrolyzed by addition of distilled water, followed by extraction with ethyl acetate. The combined organic layers were dried over $MgSO_4$ and concentrated under reduced pressure to yield the silyl enol ether **18** (12.9 g, 53.0 mmol, 97%) as mixture of *E* and *Z* stereoisomers (2:3, not assigned) which was sufficiently pure to be used without further purification. Note that the minor impurity that can be observed in the NMR spectra is compound **17** that is formed by degradation of **18** during recording of the NMR spectra due to its instability in solution. GC/MS analysis showed that the product is free from starting material **17**. ^{1}H NMR (400 MHz, $CDCl_3$) δ = 7.57 (dd, $^3J_{H,H}$ = 10.6 Hz, $^3J_{H,H}$ = 17.2 Hz, 1H, CH, major stereoisomer), 6.14 (dd, $^3J_{H,H}$ = 10.5 Hz, $^3J_{H,H}$ = 17.1 Hz, 1H, CH, minor stereoisomer), 5.82 (ddd, $^5J_{H,H}$ = 0.6 Hz, $^2J_{H,H}$ = 2.2 Hz, $^3J_{H,H}$ = 17.1 Hz, 1H, CH_2, major stereoisomer), 5.73 (ddd, $^3J_{H,H}$ = 17.1 Hz, $^5J_{H,H}$ = 0.5 Hz, $^2J_{H,H}$ = 1.3 Hz, 1H, CH_2, minor stereoisomer), 5.42 (ddd, $^5J_{H,H}$ = 1.5 Hz, $^2J_{H,H}$ = 2.1 Hz, $^3J_{H,H}$ = 10.6 Hz, 1H, CH_2, major stereoisomer), 5.33 (ddd, $^5J_{H,H}$ = 0.5 Hz, $^2J_{H,H}$ = 1.3 Hz, $^3J_{H,H}$ = 10.4 Hz, 1H, CH_2, minor stereoisomer), 5.18 (s, 1H, CH, minor stereoisomer), 4.96 (d, $^5J_{H,H}$ = 1.5 Hz, 1H, CH, major stereoisomer), 1.47 (s, 9H, 3x CH_3, major stereoisomer), 1.46 (s, 9H, 3x CH_3, minor stereoisomer), 0.27 (s, 9H, 3x CH_3, major stereoisomer), 0.26 (s, 9H, 3x CH_3, minor stereoisomer) ppm. ^{13}C NMR (100 MHz, $CDCl_3$) δ = 166.5 (C_q, major stereoisomer), 165.0 (C_q, minor stereoisomer), 162.1 (C_q, major stereoisomer), 160.0 (C_q, minor stereoisomer), 135.9 (CH, minor stereoisomer), 130.7 (CH, major stereoisomer), 121.0 (CH_2, major stereoisomer),

120.2 (CH_2, minor stereoisomer), 104.9 (CH, minor stereoisomer), 103.2 (CH, major stereoisomer), 79.7 (C_q, major stereoisomer), 79.2 (C_q, minor stereoisomer), 28.3 (3x CH_3, major stereoisomer), 28.3 (3x CH_3, minor stereoisomer), 0.6 (3x CH_3, minor stereoisomer), 0.1 (3x CH_3, major stereoisomer) ppm. IR (ATR) $\tilde{\nu}$ = 2979 (w), 2934 (w), 1734 (w), 1703 (m), 1655 (m), 1635 (m), 1586 (m), 1574 (m), 1456 (w), 1415 (m), 1368 (m), 1301 (w), 1249 (s), 1129 (s), 1064 (m), 1042 (w), 997 (w), 984 (w), 940 (w), 884 (m), 840 (s), 813 (s), 731 (w), 693 (w) cm^{-1}. λ_{max} (log ε) = 252 (3.47) nm. MS (EI, 70 eV, minor stereoisomer) *m*/*z* (%) = 243 (<1), 186 (31), 171 (100), 153 (3), 140 (5), 111 (8), 75 (44), 57 (26), 41 (25). MS (EI, 70 eV, major stereoisomer) *m*/*z* (%) = 186 (34), 171 (100), 153 (3), 140 (4), 75 (43), 57 (28), 41 (26). GC (BPX-5) *I* = 1288 (minor stereoisomer), *I* = 1321 (major stereoisomer).

tert-Butyl 1,6,7,7-tetrachloro-3-((trimethylsilyl)oxy)bicyclo[4.1.0]hept-3-ene-2-carboxylate (**19**): A solution of tetrachlorocyclopropene (10.7 g, 60.0 mmol, 2.0 eq.) and the silyl enol ether **18** (mixture of stereoisomers, 7.3 g, 30.0 mmol, 1.0 eq.) in dry toluene (22 mL) was stirred for 72 h under reflux. The reaction mixture was allowed to cool to room temperature and concentrated under reduced pressure. Column chromatography of the residue on silica gel with hexane/ethyl acetate (10:1) yielded the bicyclic Diels-Alder adduct **19** (5.0 g, 11.9 mmol, 40%) as brown oil. ^{1}H NMR (400 MHz, $CDCl_3$) δ = 4.70 (dd, $^3J_{H,H}$ = 2.7 Hz, $^3J_{H,H}$ = 5.5 Hz, 1H, CH), 3.53 (dd, $^5J_{H,H}$ = 2.5 Hz, $^5J_{H,H}$ = 2.5 Hz, 1H), 3.27 (ddd, $^3J_{H,H}$ = 2.7 Hz, $^4J_{H,H}$ = 2.7 Hz, $^2J_{H,H}$ = 17.8 Hz, 1H, CH_2), 3.04 (ddd, $^5J_{H,H}$ = 2.4 Hz, $^3J_{H,H}$ = 5.5 Hz, $^2J_{H,H}$ = 17.8 Hz, 1H, CH_2), 1.49 (s, 9H, 3x CH_3), 0.18 (s, 9H, 3x CH_3) ppm. ^{13}C NMR (100 MHz, $CDCl_3$) δ = 167.6 (C_q), 146.0 (C_q), 101.1 (CH), 82.4 (C_q), 67.8 (C_q), 54.8 (C_q), 54.0 (CH), 52.8 (C_q), 33.4 (CH_2), 27.8 (3x CH_3), 0.2 (3x CH_3) ppm. IR (ATR) $\tilde{\nu}$ = 2983 (w), 2934 (w), 1648 (m),

1613 (s), 1450 (w), 1431 (w), 1402 (m), 1368 (m), 1343 (s), 1255 (m), 1210 (m), 1146 (s), 1084 (w), 1057 (w), 967 (w), 922 (w), 898 (w), 866 (m), 844 (s), 824 (s), 787 (m), 737 (w), 694 (w), 627 (m), 605 (m) cm^{-1}. λ_{max} (log ε) = 251 (4.00), 235 (3.80) nm. MS (EI, 70 eV) *m*/*z* (%) = 345 (1) $[M–TMS]^+$, 319 (8), 283 (26), 247 (34), 174 (18), 93 (17), 73 (59), 57 (100), 41 (30). GC (BPX-5) *I* = 2020.

tert-Butyl 2,3,4-trichloro-7-oxocyclohepta-1,3-diene-1-carboxylate (**20**) and *tert*-butyl 3,4-dichloro-2-fluoro-7-oxocyclohepta-1,3-diene-1-carboxylate (**21**): A solution of **19** (420 mg, 1.0 mmol, 1.0 eq.) in dry THF (5 mL) was treated with TBAF (1 M in THF, 1.2 mL, 1.2 mmol, 1.2 eq.) and stirred for 1 h at room temperature. The reaction was quenched by addition of distilled water, followed by extraction with ether. The combined organic layers were dried over $MgSO_4$ and concentrated under reduced pressure. Column chromatography on silica gel with hexane/ethyl acetate (15:1) yielded the dihydrotropones **20** (49 mg, 0.16 mmol, 16%) and **21** (59 mg, 0.17 mmol, 17%) as orange oils.

Analytical data for **20**: TLC (hexane/ethyl acetate 5:1) R_F = 0.42. ^{1}H NMR (400 MHz, C_6D_6) δ = 1.94 (t, $^3J_{H,H}$ = 6.3 Hz, 2H, CH_2), 1.62 (t, $^3J_{H,H}$ = 6.3 Hz, 2H, CH_2), 1.38 (s, 9 H, 3x CH_3) ppm. ^{13}C NMR (100 MHz, C_6D_6) δ = 191.7 (C_q), 163.1 (C_q), 143.3 (C_q), 138.0 (C_q), 131.1 (C_q), 126.4 (C_q), 84.1 (C_q), 36.9 (CH_2), 31.9 (CH_2), 28.7 (3x CH_3) ppm. IR (ATR) $\tilde{\nu}$ = 2979 (w), 2933 (w), 1732 (s), 1682 (m), 1590 (w), 1455 (w), 1394 (w), 1369 (m), 1342 (w), 1304 (m), 1246 (s), 1152 (s), 1055 (m), 958 (w), 891 (s), 841 (m), 790 (w), 727 (w), 606 (m) cm^{-1}. λ_{max} (log ε) = 297 (3.93), 229 (3.78) nm. MS (EI, 70 eV) *m*/*z* (%) = 310 (1) $[M]^+$, 265 (2), 255 (23), 237 (100), 210 (34), 183 (12), 146 (16), 111 (22), 75 (7), 57 (60), 41 (99). GC (BPX-5) *I* = 1393. HR-MS (CI+) calc. for $C_{12}H_{14}Cl_3O_3^+$: *m*/*z* = 311.00030; found: *m*/*z* = 310.99798.

Analytical data for **21**: TLC (hexane/ethyl acetate 5:1) R_F = 0.36. ^{1}H NMR (400 MHz, C_6D_6) δ = 1.79 – 1.74 (m, 2H, CH_2), 1.67 – 1.63 (m, 2H, CH_2), 1.46 (s, 9H, 3x CH_3) ppm. ^{13}C NMR (100 MHz, C_6D_6) δ = 193.0 (d, $^3J_{C,F}$ = 10.6 Hz, C_q), 164.7 (d, $^1J_{C,F}$ = 288.2 Hz, C_q), 160.6 (d, $^3J_{C,F}$ = 3.9 Hz, C_q), 126.7 (d, $^3J_{C,F}$ = 7.8 Hz, C_q), 124.6 (d, $^2J_{C,F}$ = 13.2 Hz, C_q), 120.6 (d, $^2J_{C,F}$ = 11.0 Hz, C_q), 83.9 (C_q), 36.1 (CH_2), 28.7 (3x CH_3), 28.2 (d, $^4J_{C,F}$ = 3.1 Hz, CH_2) ppm. ^{19}F NMR (188 MHz, C_6D_6) δ = –95.0 ppm. IR (ATR) $\tilde{\nu}$ = 2979 (w), 2933 (w), 1729 (s), 1684 (m), 1619 (m), 1565 (w), 1457 (w), 1394 (w), 1362 (s), 1336 (m), 1288 (w), 1252 (s), 1206 (w), 1157 (s), 1123 (s), 1052 (w), 992 (w), 893 (s), 868 (w), 842 (m), 797 (w), 740 (w), 698 (w), 622 (m), 579 (w) cm^{-1}. λ_{max} (log ε) = 291 (4.09), 228 (3.57), 223 (3.56) nm. MS (EI, 70 eV) *m*/*z* (%) = 294 (1) $[M]^+$, 239 (30), 221 (100), 194 (43), 165 (15), 130 (22), 109 (10), 95 (15), 75 (6), 57 (73), 41 (40). GC (BPX-5) *I* = 1903. HR-MS (ESI) calc. for $C_{12}H_{14}Cl_2FO_3^+$: *m*/*z* = 295.02985; found: *m*/*z* = 295.03426.

tert-Butyl 3,4-dichloro-2-methoxy-7-oxocyclohepta-1,3-diene-1-carboxylate (**22**): A solution of **19** (3.0 g, 7.18 mmol, 1.0 eq.) in dry MeOH (72 mL) was treated with Na_2CO_3 (0.99 g, 9.33 mmol, 1.3 eq.) and stirred for 12 h at room temperature. The reaction was quenched by addition of distilled water (500 mL), followed by extraction with ether (3 x 200 mL). The combined organic layers were dried over $MgSO_4$ and concentrated under reduced pressure. Column chromatography on silica gel with hexane/ethyl acetate (10:1) yielded the dihydrotropones **20** (120 mg, 0.39 mmol, 6%) and **22** (900 mg, 2.94 mmol, 41%) as orange oils.

Analytical data for **22**: TLC (hexane/ethyl acetate 5:1) R_F = 0.16. ^{1}H NMR (400 MHz, C_6D_6) δ = 3.49 (s, 3H, CH_3), 2.03 (t, $^3J_{H,H}$ = 6.7 Hz, 2H, CH_2), 1.80 (t, $^3J_{H,H}$ = 6.8 Hz, 2H, CH_2), 1.47 (s, 9H, 3x CH_3) ppm. ^{13}C NMR (100 MHz, C_6D_6) δ = 194.2 (C_q), 165.0

(C_q), 163.9 (C_q), 129.1 (C_q), 123.8 (C_q), 121.9 (C_q), 82.9 (C_q), 59.5 (CH_3), 37.4 (CH_2), 30.6 (CH_2), 28.7 (3x CH_3) ppm. IR (ATR) $\tilde{\nu}$ = 2981 (w), 2934 (w), 1708 (m), 1662 (s), 1575 (s), 1448 (m), 1418 (w), 1391 (w), 1354 (m), 1324 (s), 1252 (s), 1161 (s), 1138 (s), 1051 (m), 1022 (w), 1008 (w), 981 (w), 942 (w), 887 (s), 867 (m), 846 (m), 801 (m), 761 (w), 739 (m), 632 (m), 600 (m) cm^{-1}. λ_{max} (log ε) = 289 (4.19), 228 (3.71) nm. MS (EI, 70 eV) *m*/*z* (%) = 306 (9) $[M]^+$, 261 (2); 250 (11), 233 (69), 220 (2), 197 (27), 179 (18), 171 (100), 143 (38), 111 (13), 99 (14), 77 (16), 57 (56). GC (BPX-5) *I* = 2040. HR-MS (ESI) calc. for $C_{13}H_{16}Cl_2O_4Na^+$: *m*/*z* = 329.03179; found: *m*/*z* = 329.03193.

3,4-Dichloro-2-methoxy-7-oxocyclohepta-1,3-diene-1-carboxylic acid (**23**): The ester **22** (100 mg, 0.33 mmol, 1.0 eq.) was dissolved in CH_2Cl_2 (3.5 mL) and was treated with TFA (0.5 mL). The mixture was stirred for 45 min. After the reaction was completed, TFA and solvent were evaporated under reduced pressure. The pure acid **23** (81 mg, 32.3 mmol, 98 %) was obtained as yellow oil. TLC (hexane/ethyl acetate 1:1) R_F = 0.15. ^{1}H NMR (300 MHz, C_6D_6) δ = 11.82 (s, 1H, COOH), 3.64 (s, 3H, CH_3), 2.00 (t, $^3J_{H,H}$ = 6.7 Hz, 2H, CH_2), 1.81 (t, $^3J_{H,H}$ = 6.7 Hz, 2H, CH_2) ppm. ^{13}C NMR (75 MHz, C_6D_6) δ = 200.6 (C_q), 175.5 (C_q), 164.3 (C_q), 131.1 (C_q), 128.5 (C_q), 112.5 (C_q), 63.1 (CH_3), 36.7 (CH_2), 29.2 (CH_2) ppm. IR (ATR) $\tilde{\nu}$ = 2955 (w), 2861 (w), 1730 (s), 1667 (w), 1569 (s), 1447 (s), 1407 (m), 1345 (s), 1321 (s), 1234 (w), 1181 (w), 1149 (w), 1128 (w), 1049 (w), 1020 (w), 1003 (w), 963 (w), 891 (s), 852 (w), 803 (m), 736 (w), 693 (w), 655 (m), 585 (m) cm^{-1}. λ_{max} (log ε) = 301 (3.99), 231 (3.79) nm. HR-MS (ESI) calc. for $C_9H_9Cl_2O_4^+$: *m*/*z* = 250.98724; found: *m*/*z* = 250.98735.

References

1. Y.-S. Do, R. Sun, H. J. Kim, J. E. Yeo, S.-H. Bae, S. Koo, *J. Org. Chem.,* **2009,** *74,* 917–920.

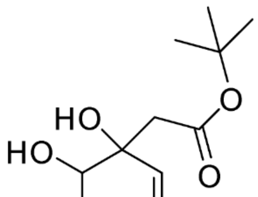

9a

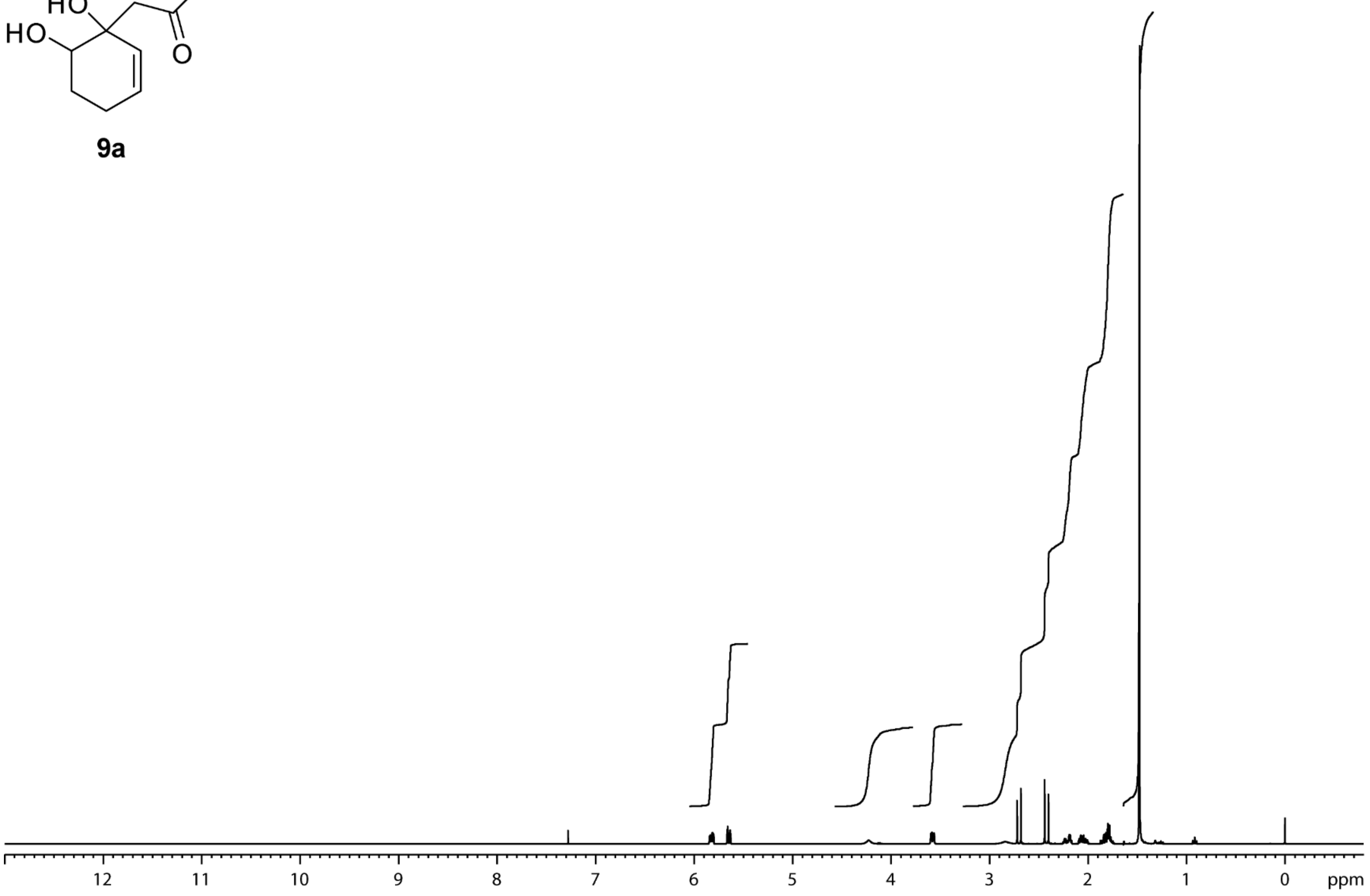


```
Current Data Parameters
NAME        brn119886_od
EXPNO                  1
PROCNO                 1

F2  Acquisition Parameters
Date_           20130226
Time                9.54
INSTRUM           drx400
PROBHD   5 mm QNP 1H/13
PULPROG             zg30
TD                 65536
SOLVENT            CDCl3
NS                    64
DS                     2
SWH             8278.146 Hz
FIDRES          0.126314 Hz
AQ             3.9584243 sec
RG                    57
DW                60.400 usec
DE                  6.00 usec
TE                 298.2 K
D1            1.00000000 sec
TD0                    1

======== CHANNEL f1 ========
NUC1                  1H
P1                 10.20 usec
PL1                 2.00 dB
SFO1         399.8924689 MHz

F2  Processing parameters
SI                 32768
SF           399.8900053 MHz
SR                  5.25 Hz
WDW                   EM
SSB                    0
LB                  0.00 Hz
GB                     0
PC                  1.40
```

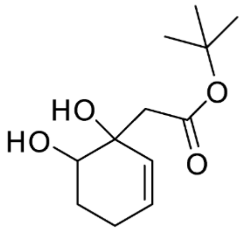

9a

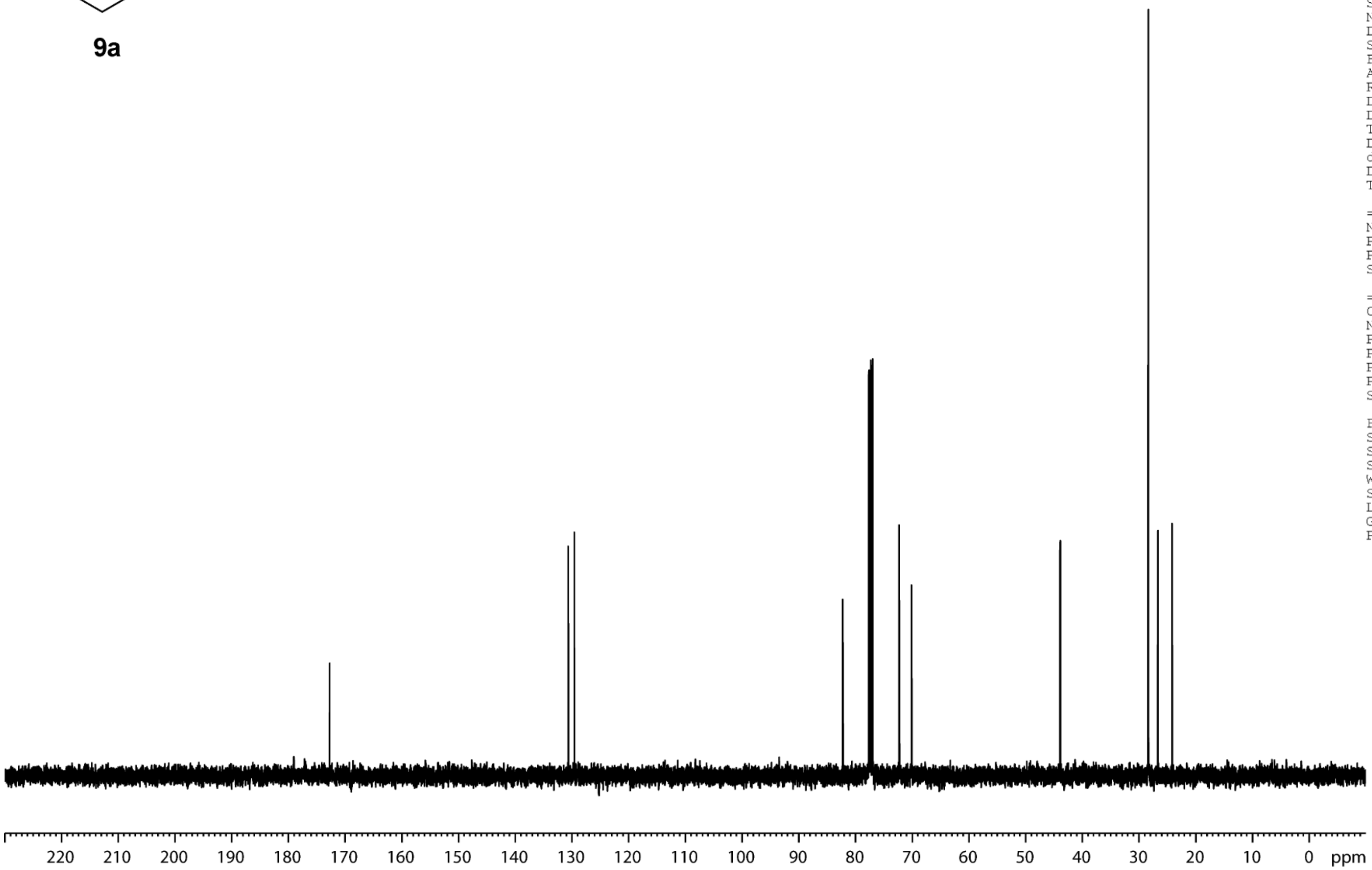


```
Current Data Parameters
NAME        brn119886_od
EXPNO                  2
PROCNO                 1

F2  Acquisition Parameters
Date_           20130226
Time               10.03
INSTRUM           drx400
PROBHD   5 mm QNP 1H/13
PULPROG          zgpg30
TD               131072
SOLVENT           CDCl3
NS                    96
DS                     4
SWH          26315.789 Hz
FIDRES        0.200774 Hz
AQ           2.4904180 sec
RG                  8192
DW              19.000 usec
DE               6.00 usec
TE               299.2 K
D1          2.00000000 sec
d11         0.03000000 sec
DELTA       1.89999998 sec
TD0                    1

======== CHANNEL f1 ========
NUC1                 13C
P1               11.00 usec
PL1               3.00 dB
SFO1       100.5635842 MHz

======== CHANNEL f2 ========
CPDPRG2          waltz16
NUC2                  1H
PCPD2            80.00 usec
PL2               2.00 dB
PL12             16.06 dB
PL13             16.06 dB
SFO2       399.8915996 MHz

F2  Processing parameters
SI                 65536
SF         100.5523935 MHz
SR               27.50 Hz
WDW                   EM
SSB                    0
LB                1.00 Hz
GB                     0
PC                  1.40
```

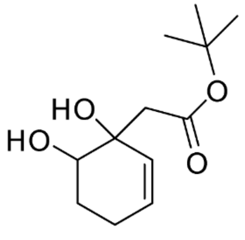

9a

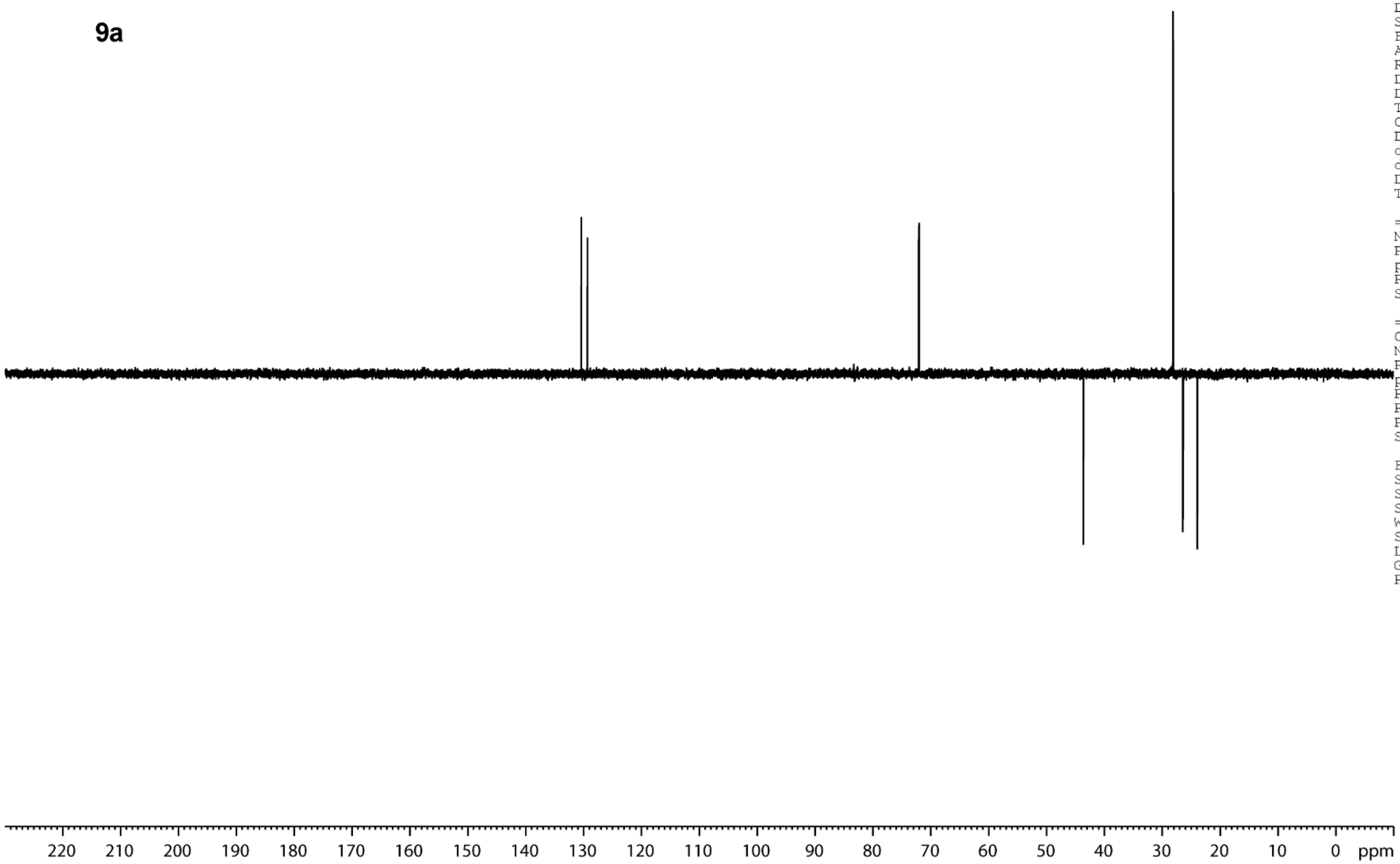


```
Current Data Parameters
NAME       brn119886_od
EXPNO                 3
PROCNO                1

F2  Acquisition Parameters
Date_          20130226
Time              10.11
INSTRUM          drx400
PROBHD   5 mm QNP 1H/13
PULPROG         dept135
TD               131072
SOLVENT           CDCl3
NS                   96
DS                    4
SWH          26315.789 Hz
FIDRES        0.200774 Hz
AQ           2.4904180 sec
RG                 8192
DW               19.000 use
DE                 7.00 use
TE                298.2 K
CNST2       145.0000000
D1           2.00000000 sec
d2           0.00344828 sec
d12          0.00002000 sec
DELTA        0.00001401 sec
TD0                   1

======== CHANNEL f1 =======
NUC1                 13C
P1                 11.00 use
p2                 22.00 use
PL1                 3.00 dB
SFO1        100.5635842 MHz

======== CHANNEL f2 =======
CPDPRG2          waltz16
NUC2                  1H
P3                 10.00 use
p4                 20.00 use
PCPD2              80.00 use
PL2                 2.00 dB
PL12               16.06 dB
SFO2        399.8915996 MHz

F2  Processing parameters
SI                 65536
SF          100.5524178 MHz
SR                  3.20 Hz
WDW                   EM
SSB                    0
LB                  1.00 Hz
GB                     0
PC                  1.40
```

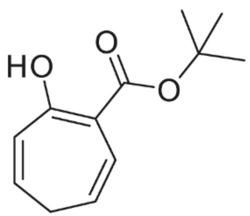

10a

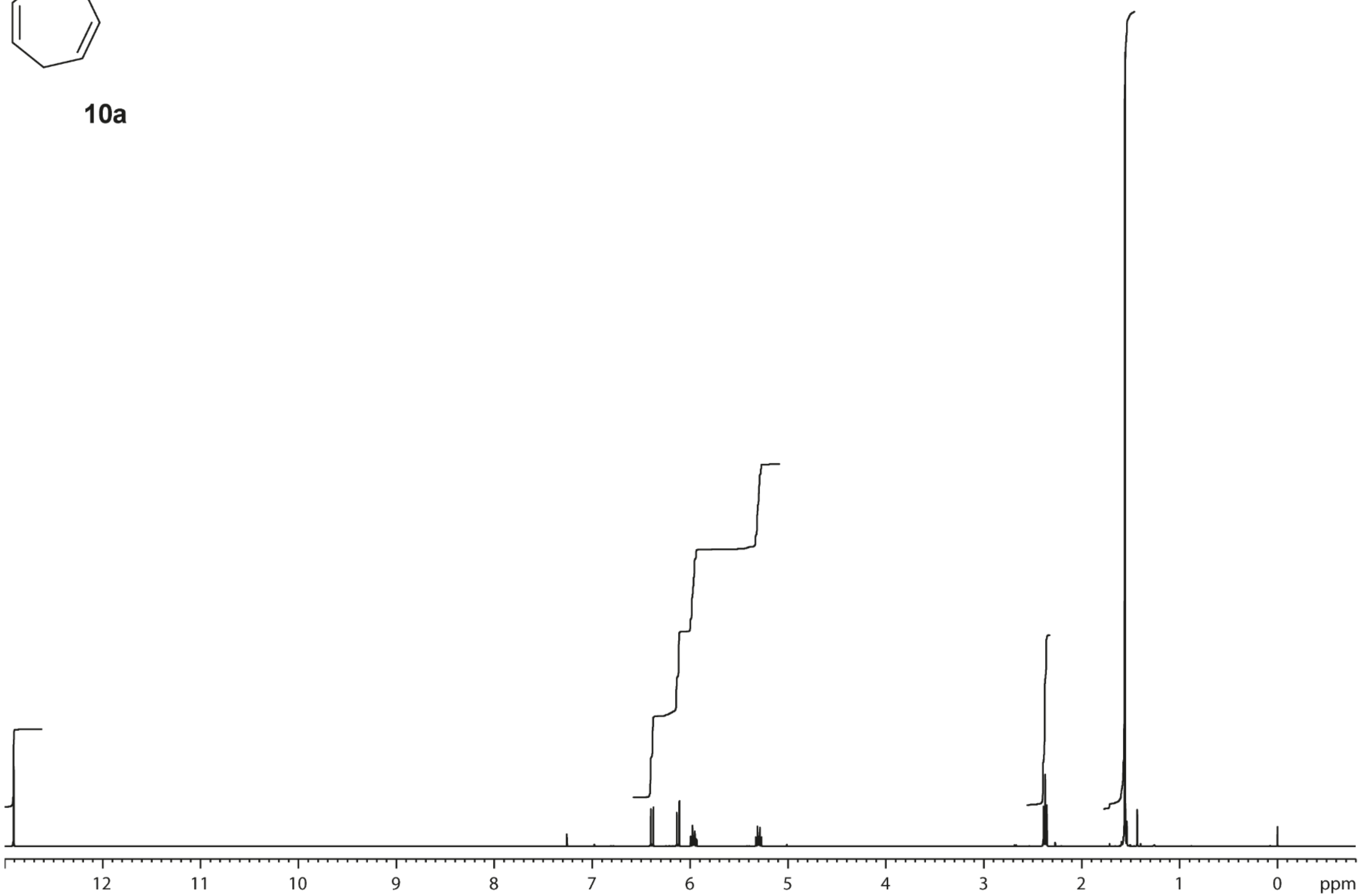

NAME	brn119907_od
EXPNO	1
PROCNO	1
Date_	20130227
Time	21.36
INSTRUM	AVIII400
PROBHD	5 mm PABBO BB-
PULPROG	zg30
TD	65536
SOLVENT	CDCl3
NS	64
DS	2
SWH	8223.685 Hz
FIDRES	0.125483 Hz
AQ	3.9846387 sec
RG	45.2
DW	60.800 use
DE	6.50 use
TE	296.0 K
D1	1.00000000 sec
TD0	1
======== CHANNEL f1 ========	
NUC1	1H
P1	10.33 use
PL1	-4.00 dB
SFO1	400.4024726 MHz
SI	32768
SF	400.4000188 MHz
SR	18.80 Hz
WDW	EM
SSB	0
LB	0.00 Hz
GB	0
PC	1.40
F1P	13.000 ppm
F2P	-0.800 ppm

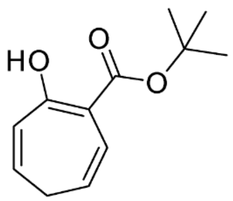

10a

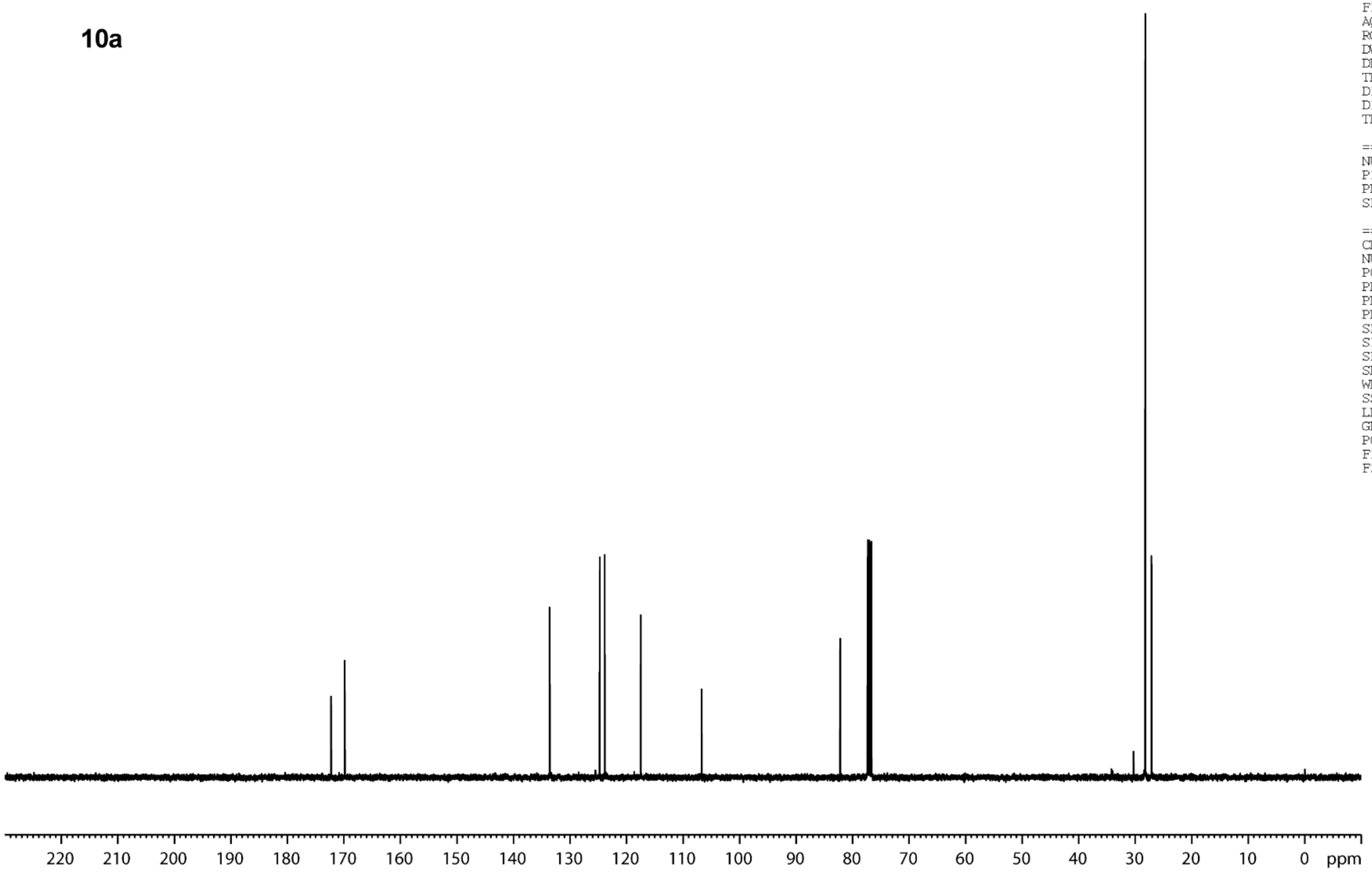

NAME	brn119907_od
EXPNO	2
PROCNO	1
Date_	20130227
Time	21.46
INSTRUM	AVIII400
PROBHD	5 mm PABBO BB
PULPROG	zgpg30
TD	131072
SOLVENT	CDCl3
NS	96
DS	4
SWH	26315.789 Hz
FIDRES	0.200774 Hz
AQ	2.4904180 sec
RG	71.8
DW	19.000 use
DE	6.50 use
TE	297.2 K
D1	2.00000000 sec
D11	0.03000000 sec
TD0	10

======== CHANNEL f1 =======

NUC1	13C
P1	8.50 use
PL1	3.00 dB
SFO1	100.6918371 MHz

======== CHANNEL f2 =======

CPDPRG2	waltz16
NUC2	1H
PCPD2	80.00 use
PL2	4.00 dB
PL12	13.78 dB
PL13	14.00 dB
SFO2	400.4016016 MHz
SI	65536
SF	100.6806656 MHz
SR	4.59 Hz
WDW	EM
SSB	0
LB	1.00 Hz
GB	0
PC	1.40
F1P	230.000 ppm
F2P	10.000 ppm

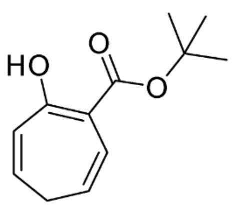
HO
O
O

10a

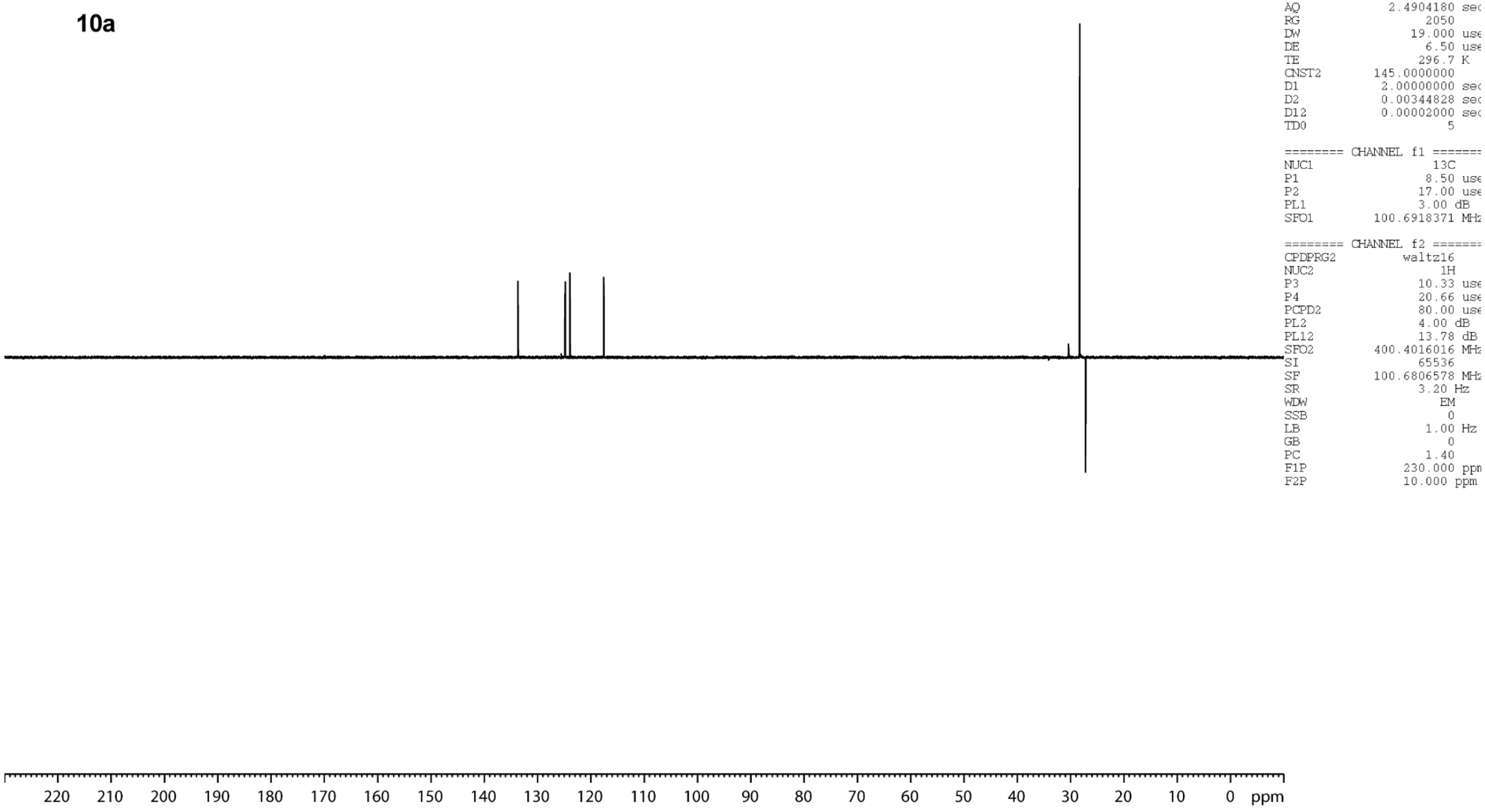
NAME brn119907_od
EXPNO 3
PROCNO 1
Date_ 20130227
Time 21.56
INSTRUM AVIII400
PROBHD 5 mm PABBO BB
PULPROG dept135
TD 131072
SOLVENT CDCl3
NS 96
DS 4
SWH 26315.789 Hz
FIDRES 0.200774 Hz
AQ 2.4904180 sec
RG 2050
DW 19.000 use
DE 6.50 use
TE 296.7 K
CNST2 145.0000000
D1 2.00000000 sec
D2 0.00344828 sec
D12 0.00002000 sec
TD0 5
======== CHANNEL f1 =======
NUC1 13C
P1 8.50 use
P2 17.00 use
PL1 3.00 dB
SFO1 100.6918371 MHz
======== CHANNEL f2 =======
CPDPRG2 waltz16
NUC2 1H
P3 10.33 use
P4 20.66 use
PCPD2 80.00 use
PL2 4.00 dB
PL12 13.78 dB
SFO2 400.4016016 MHz
SI 65536
SF 100.6806578 MHz
SR 3.20 Hz
WDW EM
SSB 0
LB 1.00 Hz
GB 0
PC 1.40
F1P 230.000 ppm
F2P 10.000 ppm
220 210 200 190 180 170 160 150 140 130 120 110 100 90 80 70 60 50 40 30 20 10 0 ppm

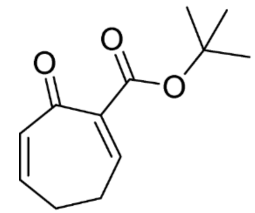

11a

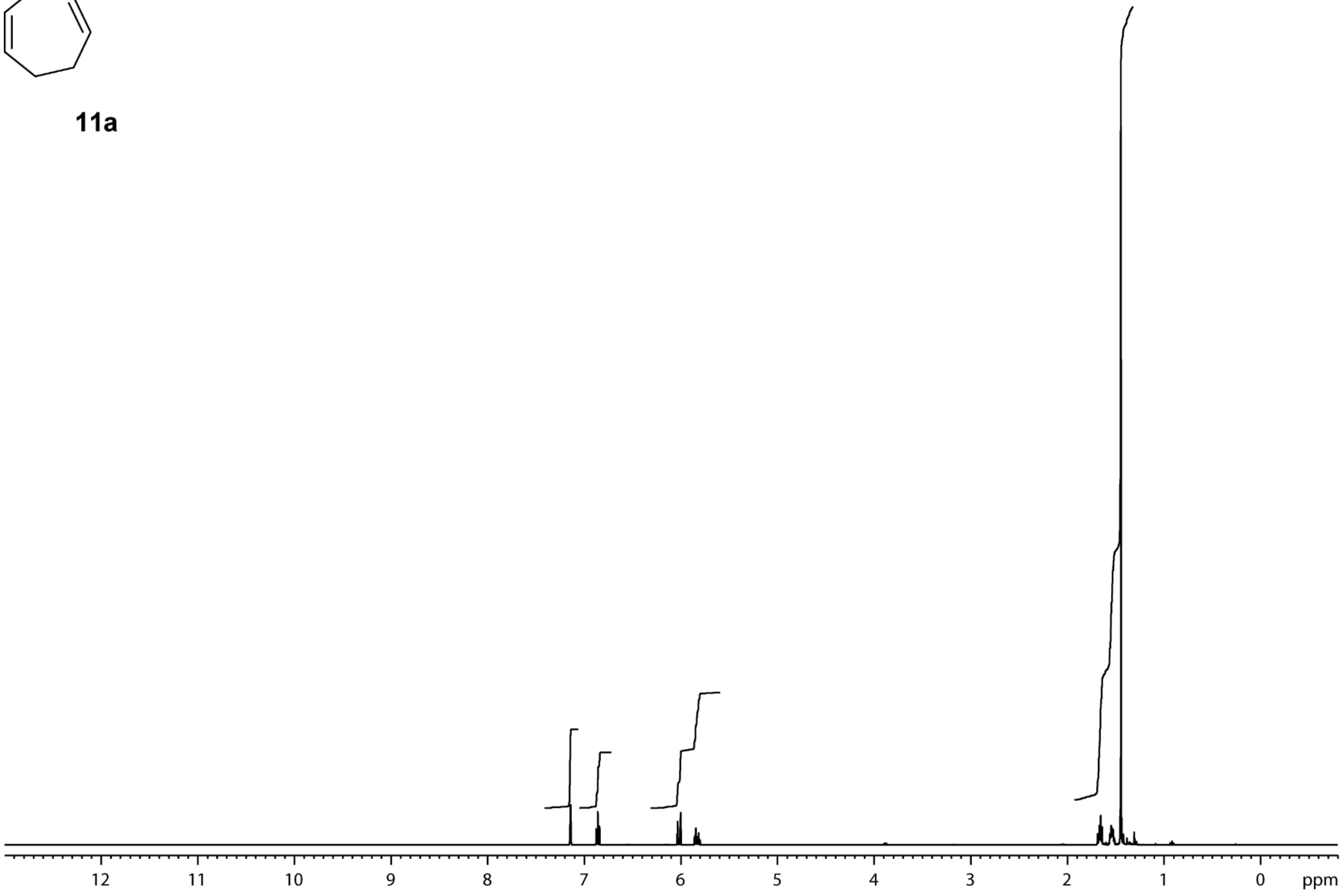

Current Data Parameters
NAME brn121113_od
EXPNO 1
PROCNO 1

F2 Acquisition Parameters
Date_ 20130624
Time 11.38
INSTRUM drx400
PROBHD 5 mm QNP 1H/13
PULPROG zg30
TD 65536
SOLVENT C6D6
NS 64
DS 2
SWH 8278.146 Hz
FIDRES 0.126314 Hz
AQ 3.9584243 sec
RG 50.8
DW 60.400 use
DE 6.00 use
TE 299.2 K
D1 1.00000000 sec
TD0 1

======== CHANNEL f1 ========
NUC1 1H
P1 10.20 use
PL1 2.00 dB
SFO1 399.8724688 MHz

F2 Processing parameters
SI 32768
SF 399.8700000 MHz
SR 0.00 Hz
WDW EM
SSB 0
LB 0.00 Hz
GB 0
PC 1.40

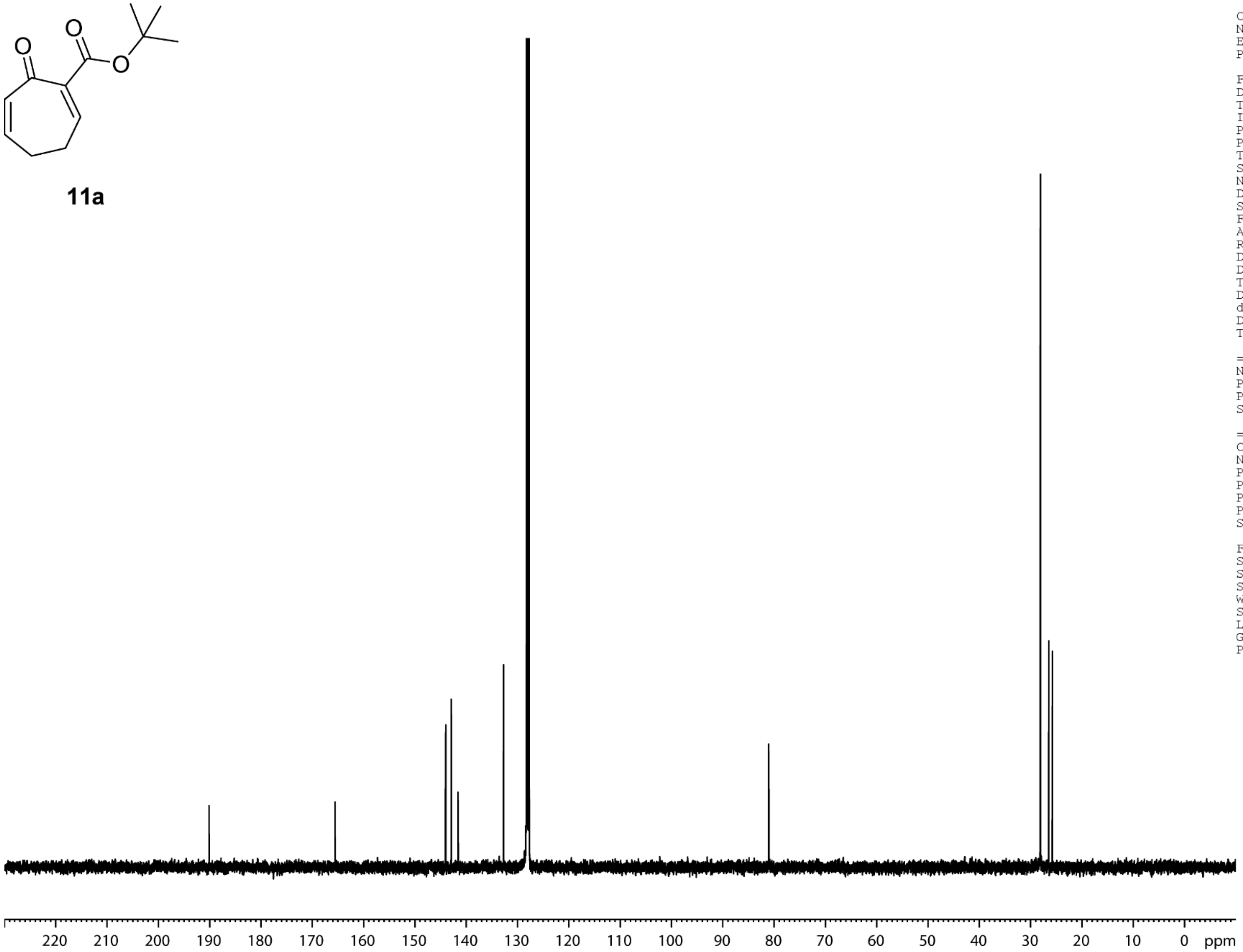


```
Current Data Parameters
NAME        brn121113_od
EXPNO                  2
PROCNO                 1

F2  Acquisition Parameters
Date_           20130624
Time               11.47
INSTRUM           drx400
PROBHD   5 mm QNP 1H/13
PULPROG          zgpg30
TD                131072
SOLVENT             C6D6
NS                    96
DS                     4
SWH         26315.789 Hz
FIDRES       0.200774 Hz
AQ          2.4904180 sec
RG                7298.2
DW            19.000 use
DE              6.00 use
TE              299.2 K
D1         2.00000000 sec
d11        0.03000000 sec
DELTA      1.89999998 sec
TD0                    1

======== CHANNEL f1 =======
NUC1                 13C
P1              11.00 use
PL1               3.00 dB
SFO1      100.5585542 MHz

======== CHANNEL f2 =======
CPDPRG2          waltz16
NUC2                  1H
PCPD2           80.00 use
PL2               2.00 dB
PL12             16.06 dB
PL13             16.06 dB
SFO2      399.8715995 MHz

F2  Processing parameters
SI                 65536
SF        100.5473556 MHz
SR              36.36 Hz
WDW                   EM
SSB                    0
LB                1.00 Hz
GB                     0
PC                  1.40
```

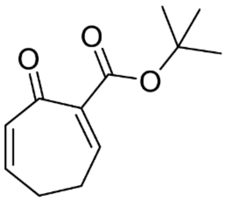

11a

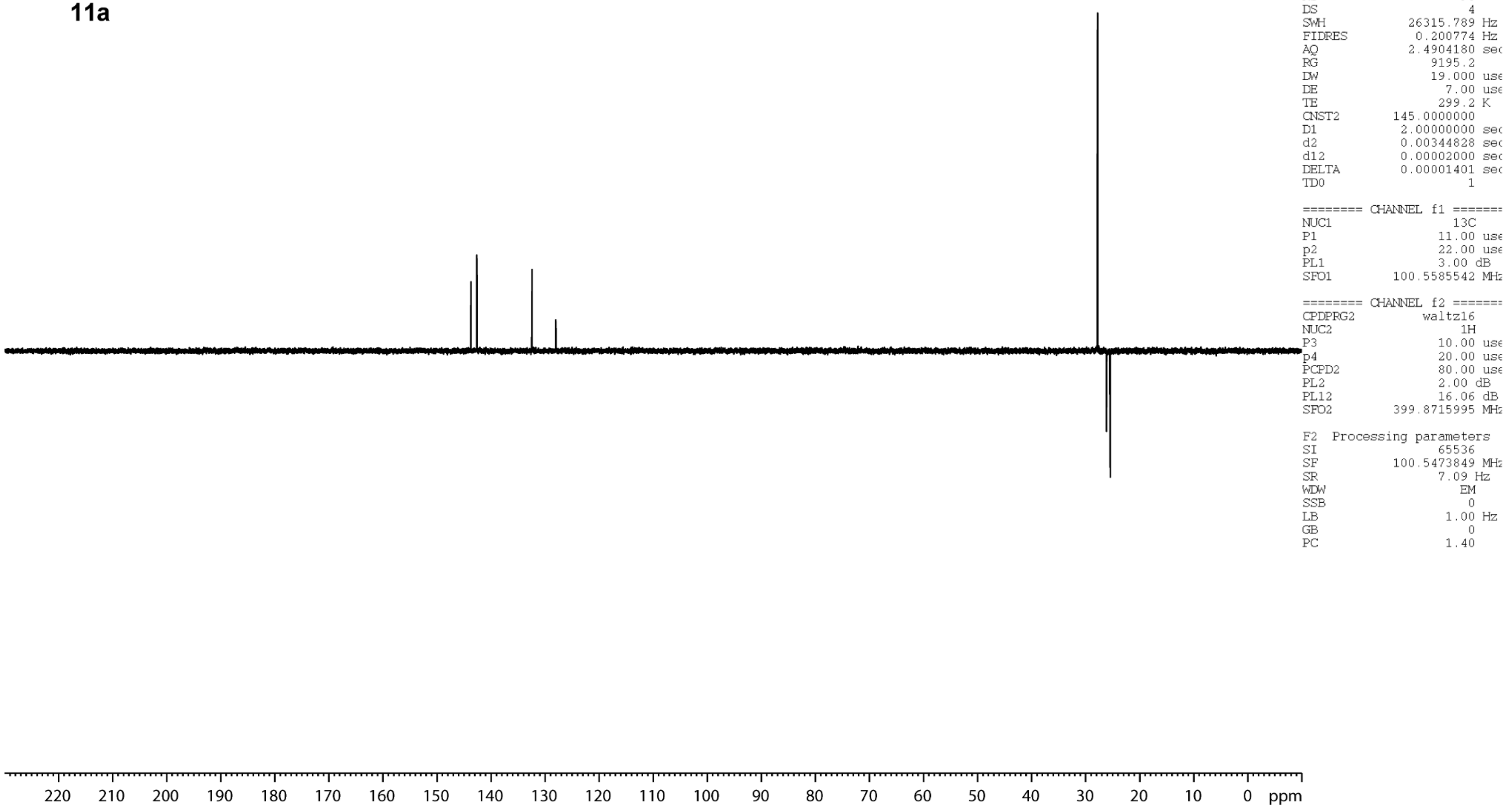

Current Data Parameters
NAME brn121113_od
EXPNO 3
PROCNO 1
F2 Acquisition Parameters
Date_ 20130624
Time 11.55
INSTRUM drx400
PROBHD 5 mm QNP 1H/13
PULPROG dept135
TD 131072
SOLVENT C6D6
NS 96
DS 4
SWH 26315.789 Hz
FIDRES 0.200774 Hz
AQ 2.4904180 sec
RG 9195.2
DW 19.000 use
DE 7.00 use
TE 299.2 K
CNST2 145.0000000
D1 2.00000000 sec
d2 0.00344828 sec
d12 0.00002000 sec
DELTA 0.00001401 sec
TD0 1
======== CHANNEL f1 ========
NUC1 13C
P1 11.00 use
p2 22.00 use
PL1 3.00 dB
SFO1 100.5585542 MHz
======== CHANNEL f2 ========
CPDPRG2 waltz16
NUC2 1H
P3 10.00 use
p4 20.00 use
PCPD2 80.00 use
PL2 2.00 dB
PL12 16.06 dB
SFO2 399.8715995 MHz
F2 Processing parameters
SI 65536
SF 100.5473849 MHz
SR 7.09 Hz
WDW EM
SSB 0
LB 1.00 Hz
GB 0
PC 1.40
220 210 200 190 180 170 160 150 140 130 120 110 100 90 80 70 60 50 40 30 20 10 0 ppm

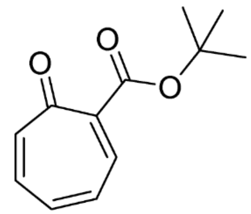

12a

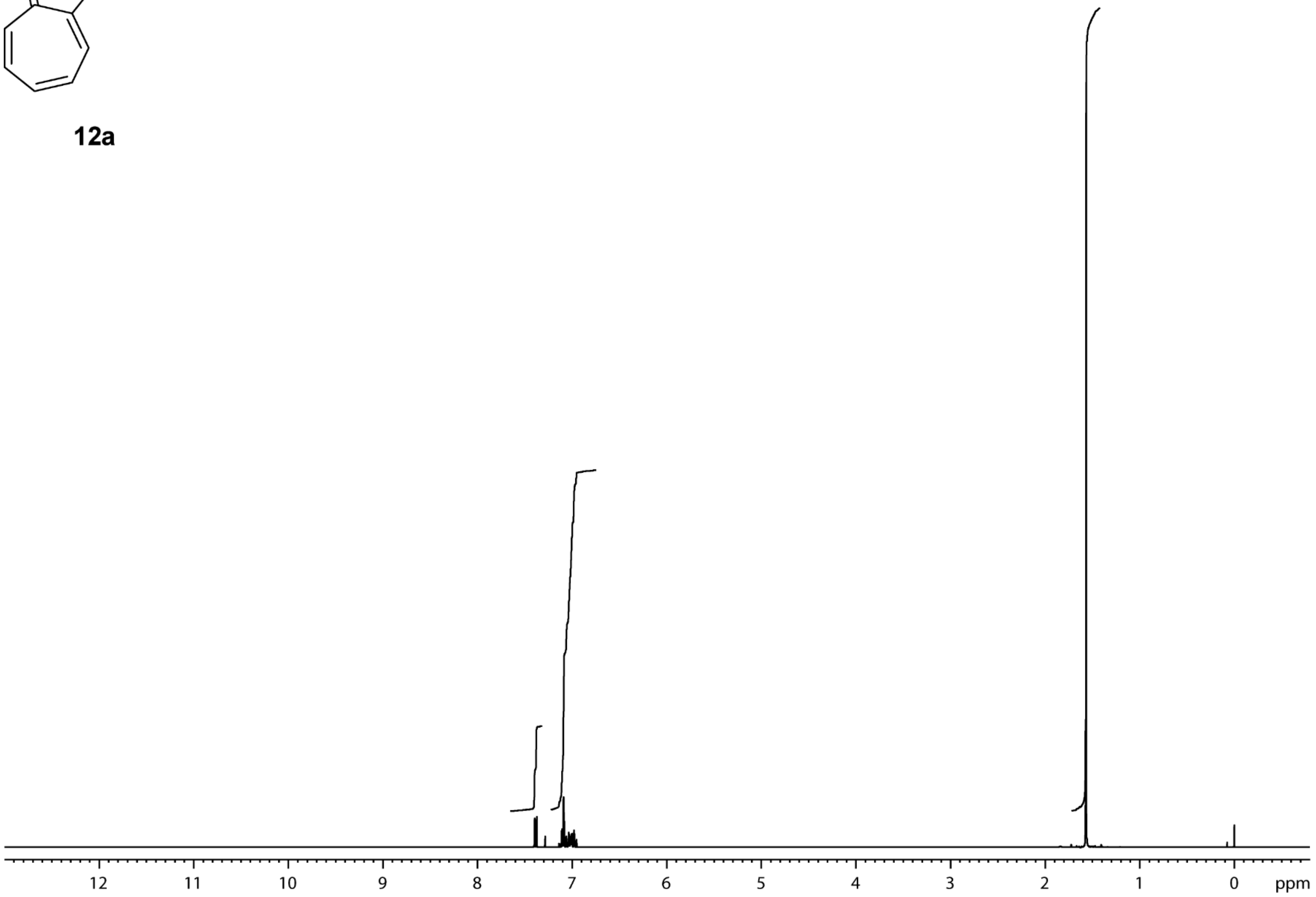


```
Current Data Parameters
NAME          brn119952_od
EXPNO                    1
PROCNO                   1

F2  Acquisition Parameters
Date_             20130301
Time                 10.31
INSTRUM             drx400
PROBHD    5 mm QNP 1H/13
PULPROG               zg30
TD                   65536
SOLVENT              CDCl3
NS                      64
DS                       2
SWH               8278.146 Hz
FIDRES            0.126314 Hz
AQ               3.9584243 sec
RG                    71.8
DW                  60.400 usec
DE                    6.00 usec
TE                   298.2 K
D1              1.00000000 sec
TD0                      1

======== CHANNEL f1 ========
NUC1                    1H
P1                   10.20 usec
PL1                   2.00 dB
SFO1           399.8924689 MHz

F2  Processing parameters
SI                   32768
SF             399.8900033 MHz
SR                    3.33 Hz
WDW                     EM
SSB                      0
LB                    0.00 Hz
GB                       0
PC                    1.40
```

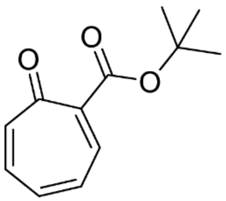

12a

Current Data Parameters
NAME brn119952_od
EXPNO 2
PROCNO 1

F2 Acquisition Parameters
Date_ 20130301
Time 11.11
INSTRUM drx400
PROBHD 5 mm QNP 1H/13
PULPROG zgpg30
TD 131072
SOLVENT CDCl3
NS 512
DS 4
SWH 26315.789 Hz
FIDRES 0.200774 Hz
AQ 2.4904180 sec
RG 10321.3
DW 19.000 usec
DE 6.00 usec
TE 299.2 K
D1 2.00000000 sec
d11 0.03000000 sec
DELTA 1.89999998 sec
TD0 1

======== CHANNEL f1 ========
NUC1 13C
P1 11.00 usec
PL1 3.00 dB
SFO1 100.5635842 MHz

======== CHANNEL f2 ========
CPDPRG2 waltz16
NUC2 1H
PCPD2 80.00 usec
PL2 2.00 dB
PL12 16.06 dB
PL13 16.06 dB
SFO2 399.8915996 MHz

F2 Processing parameters
SI 65536
SF 100.5524276 MHz
SR 6.61 Hz
WDW EM
SSB 0
LB 1.00 Hz
GB 0
PC 1.40

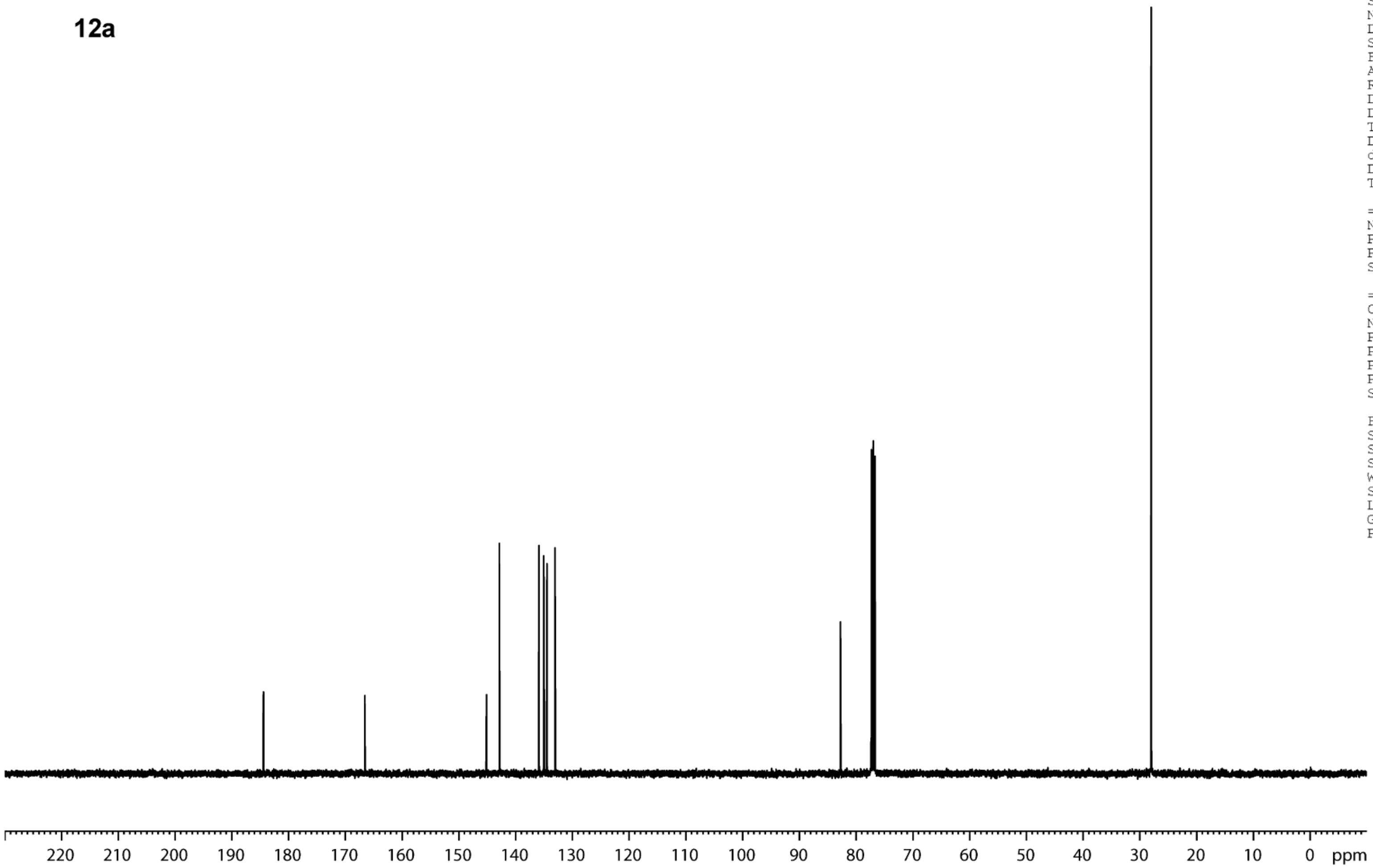

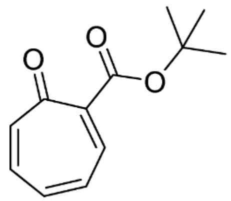

12a

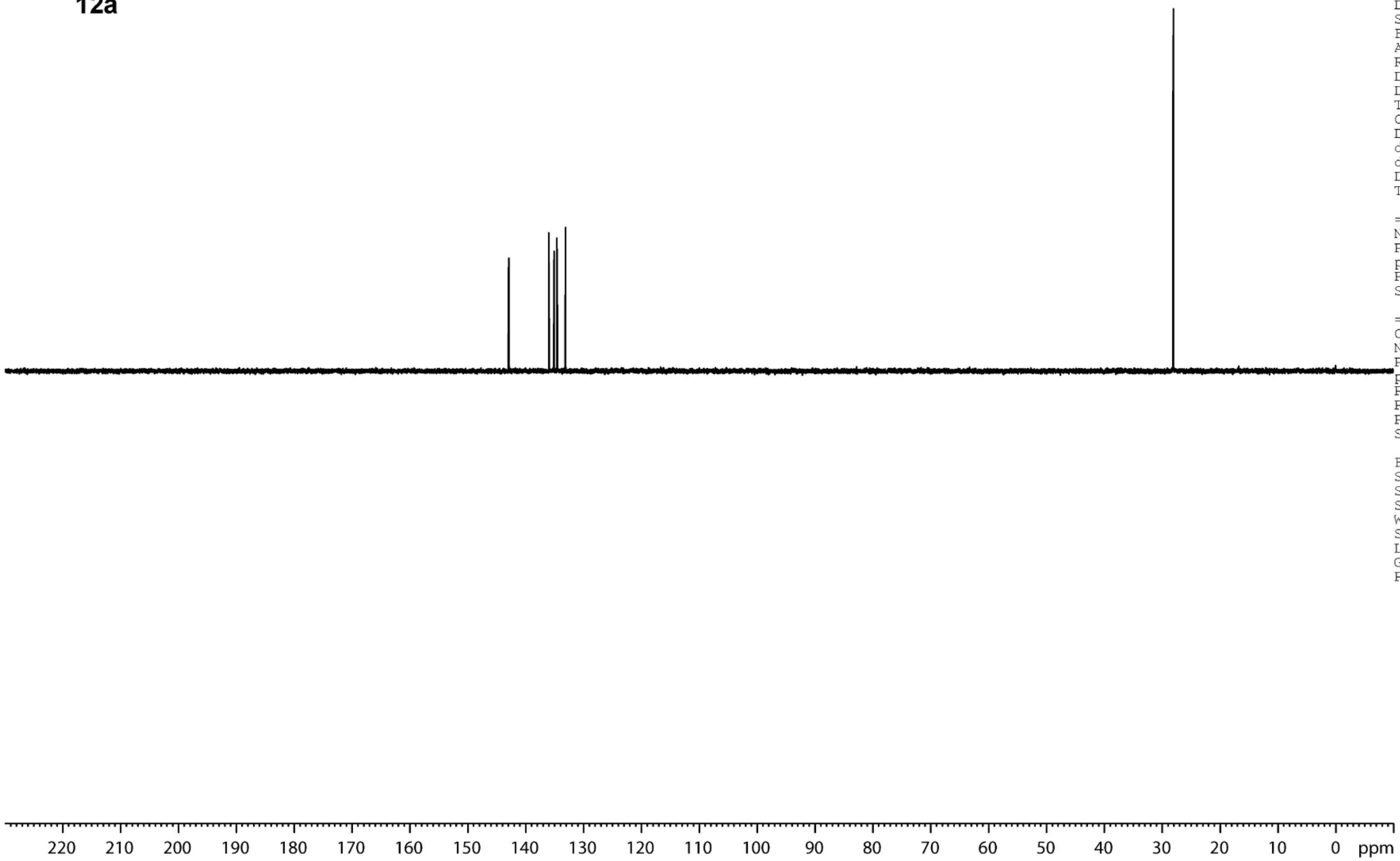

Current Data Parameters
NAME brn119952_od
EXPNO 3
PROCNO 1

F2 Acquisition Parameters
Date_ 20130301
Time 11.31
INSTRUM drx400
PROBHD 5 mm QNP 1H/13
PULPROG dept135
TD 131072
SOLVENT CDCl3
NS 256
DS 4
SWH 26315.789 Hz
FIDRES 0.200774 Hz
AQ 2.4904180 sec
RG 7298.2
DW 19.000 usec
DE 7.00 usec
TE 299.2 K
CNST2 145.0000000
D1 2.00000000 sec
d2 0.00344828 sec
d12 0.00002000 sec
DELTA 0.00001401 sec
TD0 1

======== CHANNEL f1 ========
NUC1 13C
P1 11.00 usec
p2 22.00 usec
PL1 3.00 dB
SFO1 100.5635842 MHz

======== CHANNEL f2 ========
CPDPRG2 waltz16
NUC2 1H
P3 10.00 usec
p4 20.00 usec
PCPD2 80.00 usec
PL2 2.00 dB
PL12 16.06 dB
SFO2 399.8915996 MHz

F2 Processing parameters
SI 65536
SF 100.5524178 MHz
SR 3.20 Hz
WDW EM
SSB 0
LB 1.00 Hz
GB 0
PC 1.40

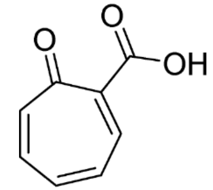

13

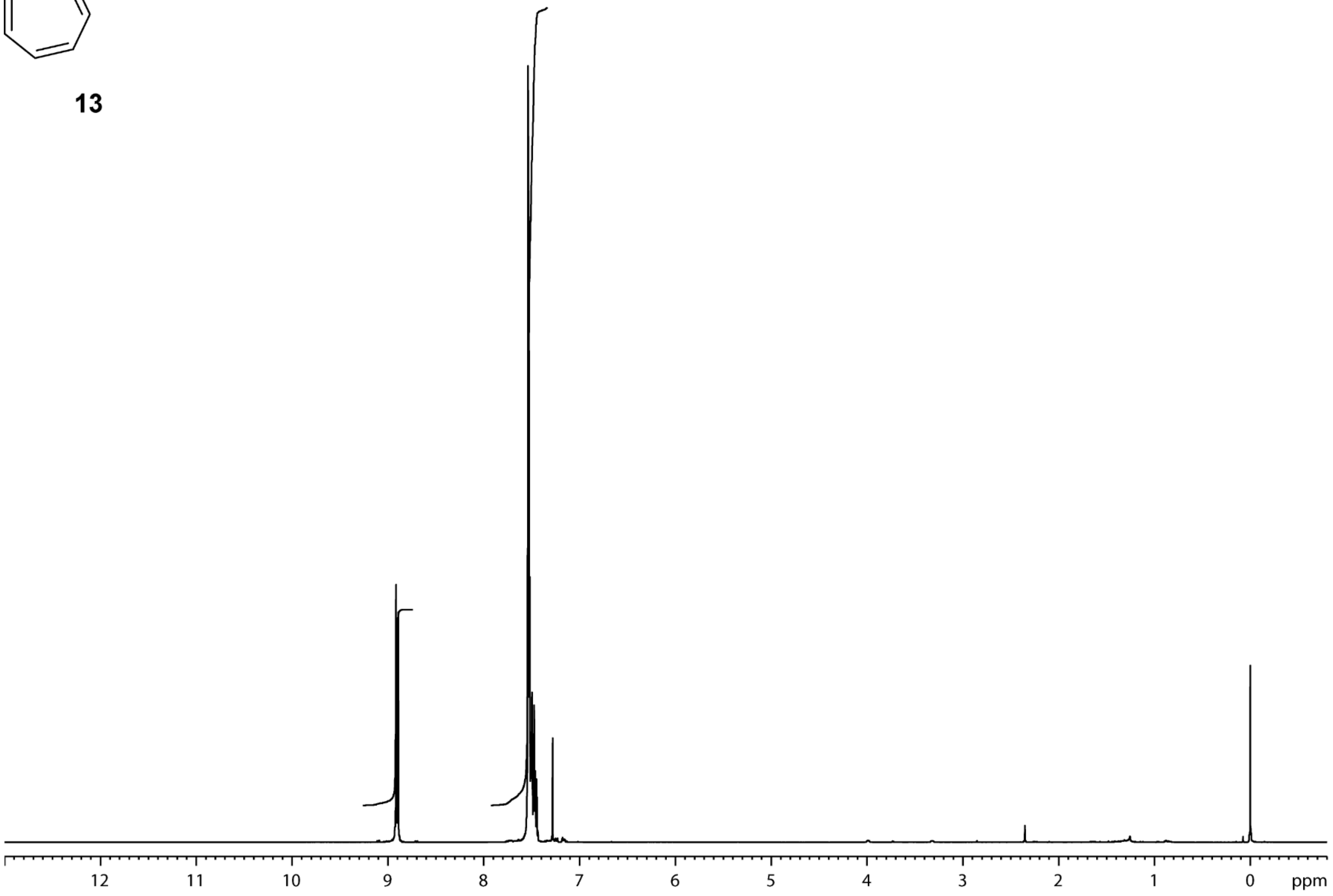


```
Current Data Parameters
NAME        brn119979_od
EXPNO                  1
PROCNO                 1

F2 - Acquisition Parameters
Date_           20130304
Time               14.38
INSTRUM           drx400
PROBHD   5 mm QNP 1H/13
PULPROG             zg30
TD                 65536
SOLVENT            CDCl3
NS                    64
DS                     2
SWH             8278.146 Hz
FIDRES          0.126314 Hz
AQ             3.9584243 sec
RG                   181
DW                60.400 usec
DE                  6.00 usec
TE                 299.2 K
D1            1.00000000 sec
TD0                    1

======== CHANNEL f1 ========
NUC1                  1H
P1                 10.20 usec
PL1                -2.00 dB
SFO1         399.8924689 MHz

F2 - Processing parameters
SI                 32768
SF           399.8900044 MHz
SR                  4.41 Hz
WDW                   EM
SSB                    0
LB                  0.00 Hz
GB                     0
PC                  1.40
```

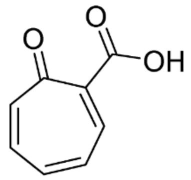

13

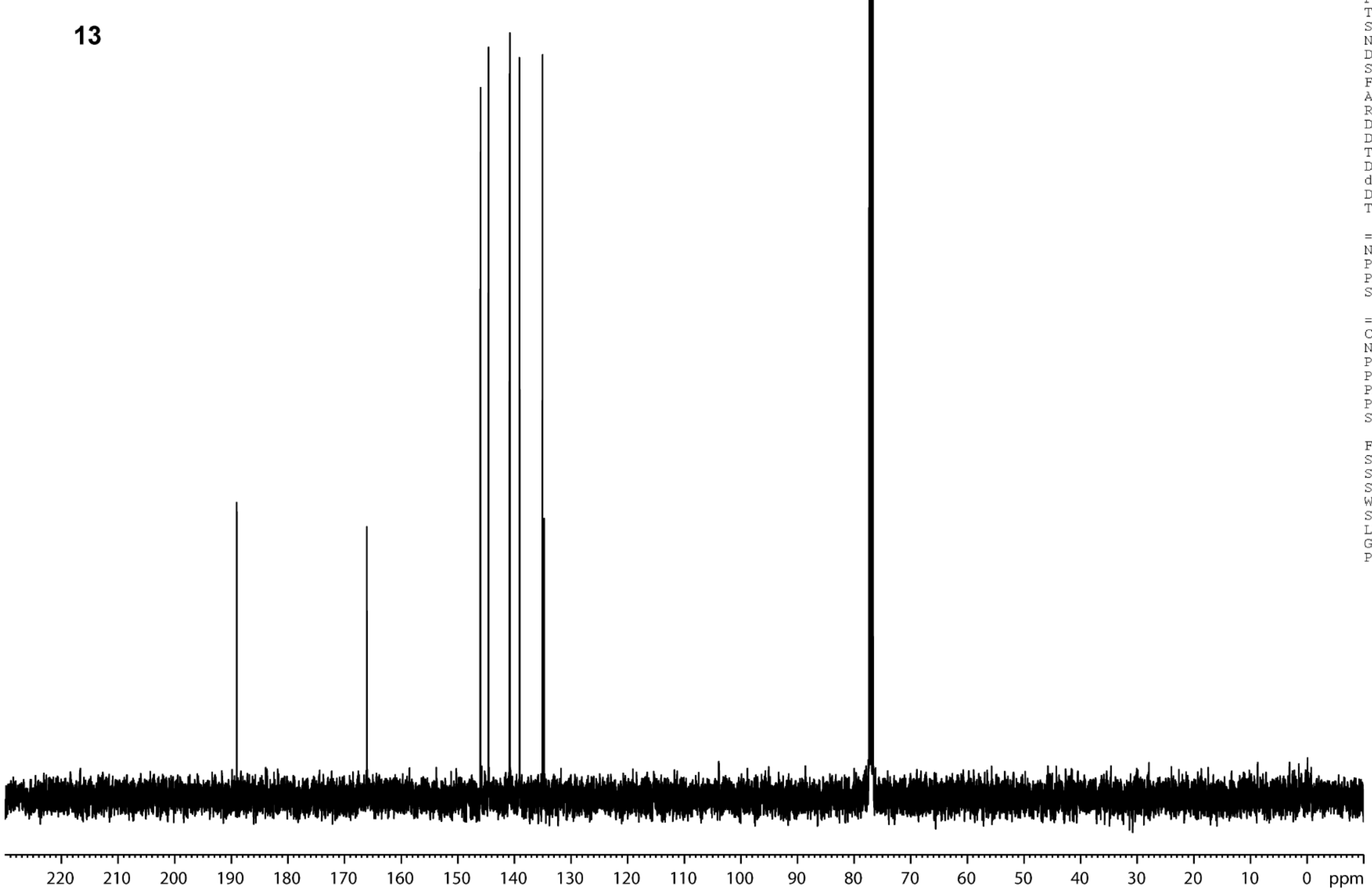


```
Current Data Parameters
NAME        brn119979_od
EXPNO                  2
PROCNO                 1

F2  Acquisition Parameters
Date_           20130304
Time               14.46
INSTRUM           drx400
PROBHD   5 mm QNP 1H/13
PULPROG          zgpg30
TD                131072
SOLVENT            CDCl3
NS                   384
DS                     4
SWH            26315.789 Hz
FIDRES          0.200774 Hz
AQ             2.4904180 sec
RG               10321.3
DW                19.000 use
DE                  6.00 use
TE                 299.2 K
D1            2.00000000 sec
d11           0.03000000 sec
DELTA         1.89999998 sec
TD0                    1

======== CHANNEL f1 =======
NUC1                 13C
P1                 11.00 use
PL1                 3.00 dB
SFO1         100.5635842 MHz

======== CHANNEL f2 =======
CPDPRG2          waltz16
NUC2                  1H
PCPD2              80.00 use
PL2                 2.00 dB
PL12               16.06 dB
PL13               16.06 dB
SFO2         399.8915996 MHz

F2  Processing parameters
SI                 65536
SF           100.5524273 MHz
SR                  6.35 Hz
WDW                   EM
SSB                    0
LB                  1.00 Hz
GB                     0
PC                  1.40
```

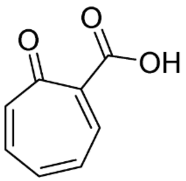

13

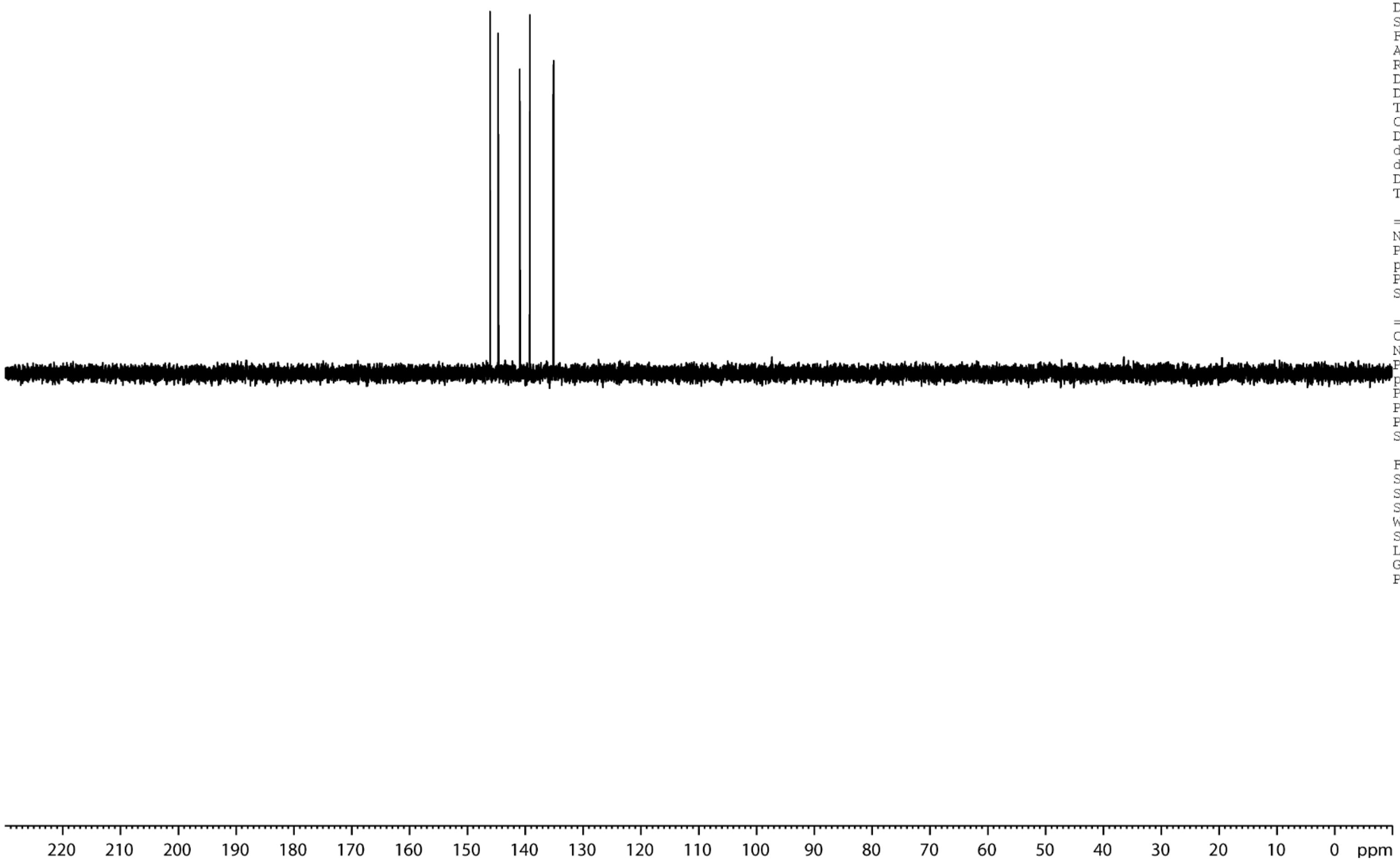


```
Current Data Parameters
NAME        brn119979_od
EXPNO                  3
PROCNO                 1

F2  Acquisition Parameters
Date_           20130304
Time               15.18
INSTRUM           drx400
PROBHD   5 mm QNP 1H/13
PULPROG         dept135
TD                131072
SOLVENT            CDCl3
NS                   288
DS                     4
SWH           26315.789 Hz
FIDRES         0.200774 Hz
AQ            2.4904180 sec
RG                  8192
DW                19.000 usec
DE                  7.00 usec
TE                 299.2 K
CNST2        145.0000000
D1            2.00000000 sec
d2            0.00344828 sec
d12           0.00002000 sec
DELTA         0.00001401 sec
TD0                    1

======== CHANNEL f1 ========
NUC1                 13C
P1                 11.00 usec
p2                 22.00 usec
PL1                 3.00 dB
SFO1         100.5635842 MHz

======== CHANNEL f2 ========
CPDPRG2          waltz16
NUC2                  1H
P3                 10.00 usec
p4                 20.00 usec
PCPD2              80.00 usec
PL2                 2.00 dB
PL12               16.06 dB
SFO2         399.8915996 MHz

F2  Processing parameters
SI                 65536
SF           100.5524178 MHz
SR                  3.20 Hz
WDW                   EM
SSB                    0
LB                  1.00 Hz
GB                     0
PC                  1.40
```

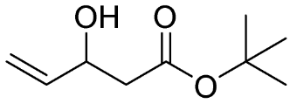

16

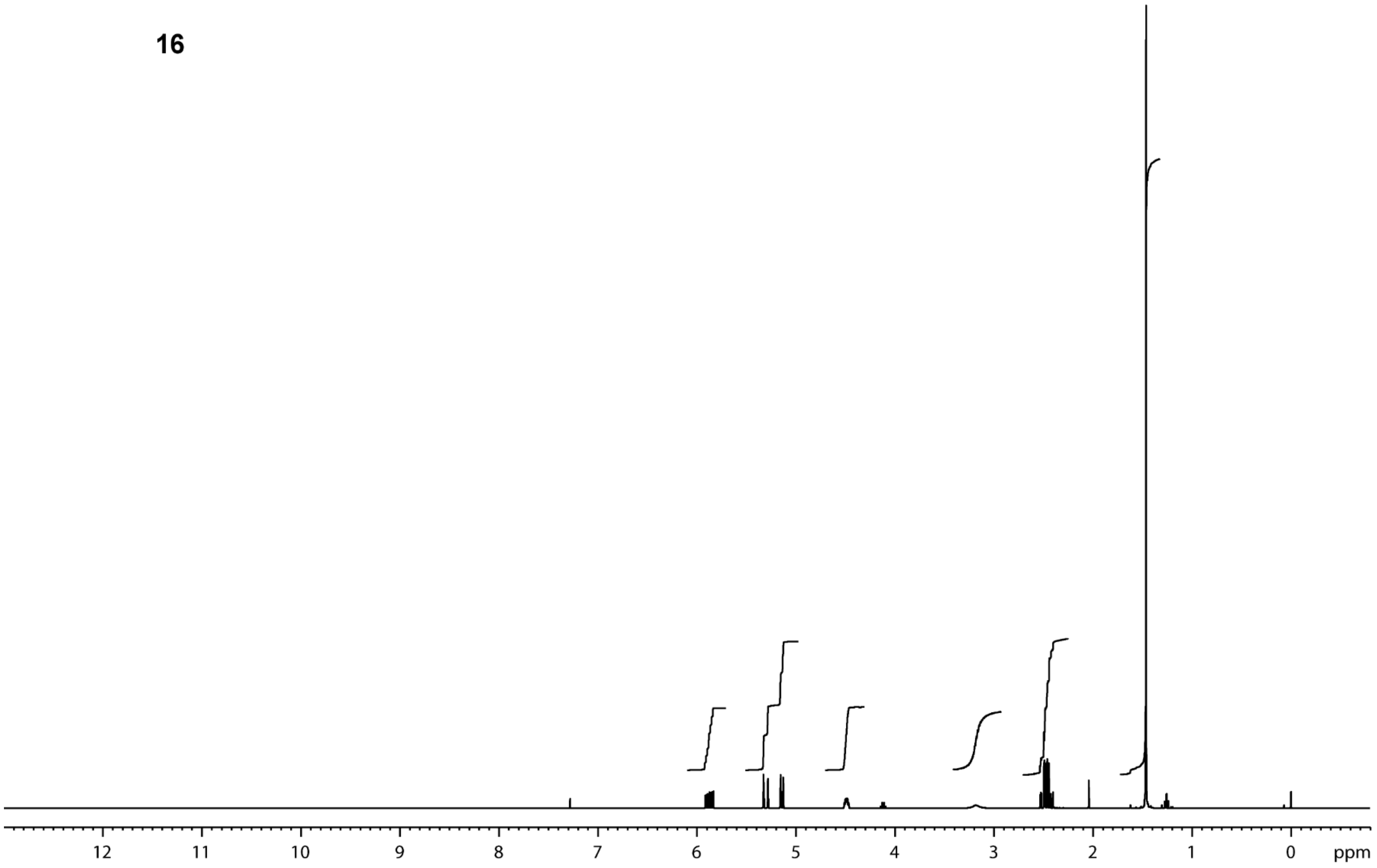


```
Current Data Parameters
NAME         rap120781_od
EXPNO                   1
PROCNO                  1

F2  Acquisition Parameters
Date_            20130516
Time                10.46
INSTRUM            drx400
PROBHD   5 mm QNP 1H/13
PULPROG              zg30
TD                  65536
SOLVENT             CDCl3
NS                     64
DS                      2
SWH              8278.146 Hz
FIDRES           0.126314 Hz
AQ              3.9584243 sec
RG                   50.8
DW                 60.400 usec
DE                   6.00 usec
TE                  299.2 K
D1             1.00000000 sec
TD0                     1

======== CHANNEL f1 ========
NUC1                   1H
P1                  10.20 usec
PL1                  2.00 dB
SFO1          399.8924689 MHz

F2  Processing parameters
SI                  32768
SF            399.8900044 MHz
SR                   4.38 Hz
WDW                    EM
SSB                     0
LB                   0.00 Hz
GB                      0
PC                   1.40
```

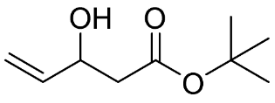

16

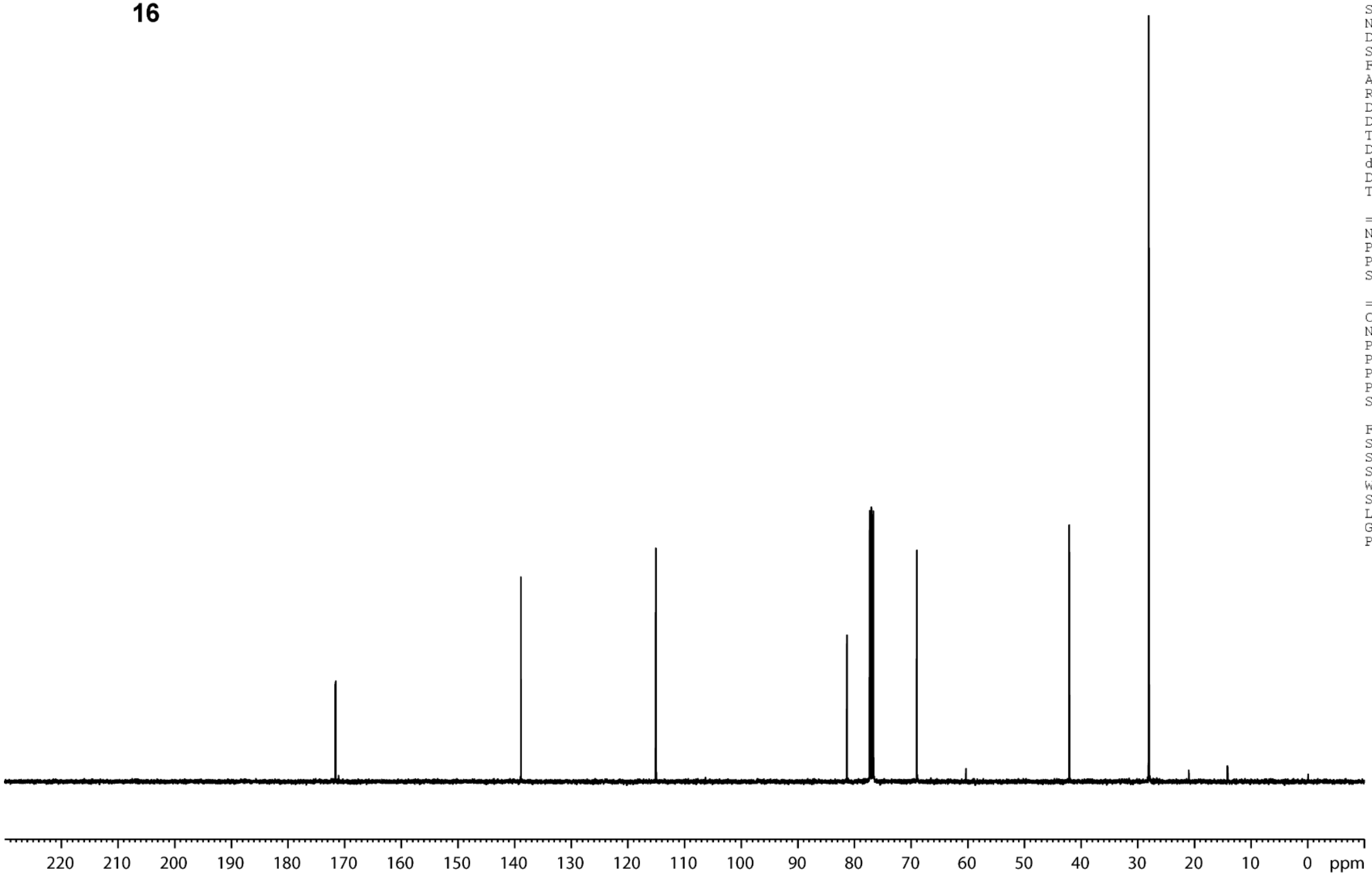


```
Current Data Parameters
NAME        rap120781_od
EXPNO                  2
PROCNO                 1

F2  Acquisition Parameters
Date_           20130516
Time               12.05
INSTRUM           drx400
PROBHD   5 mm QNP 1H/13
PULPROG          zgpg30
TD                131072
SOLVENT            CDCl3
NS                  1024
DS                     4
SWH            26315.789 Hz
FIDRES          0.200774 Hz
AQ             2.4904180 sec
RG                9195.2
DW                19.000 usec
DE                  6.00 usec
TE                 300.2 K
D1            2.00000000 sec
d11           0.03000000 sec
DELTA         1.89999998 sec
TD0                    1

======== CHANNEL f1 ========
NUC1                 13C
P1                 11.00 usec
PL1                 3.00 dB
SFO1         100.5635842 MHz

======== CHANNEL f2 ========
CPDPRG2          waltz16
NUC2                  1H
PCPD2              80.00 usec
PL2                 2.00 dB
PL12               16.06 dB
PL13               16.06 dB
SFO2         399.8915996 MHz

F2  Processing parameters
SI                 65536
SF           100.5524246 MHz
SR                  3.62 Hz
WDW                   EM
SSB                    0
LB                  1.00 Hz
GB                     0
PC                  1.40
```

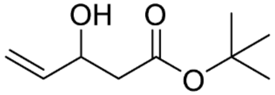

16

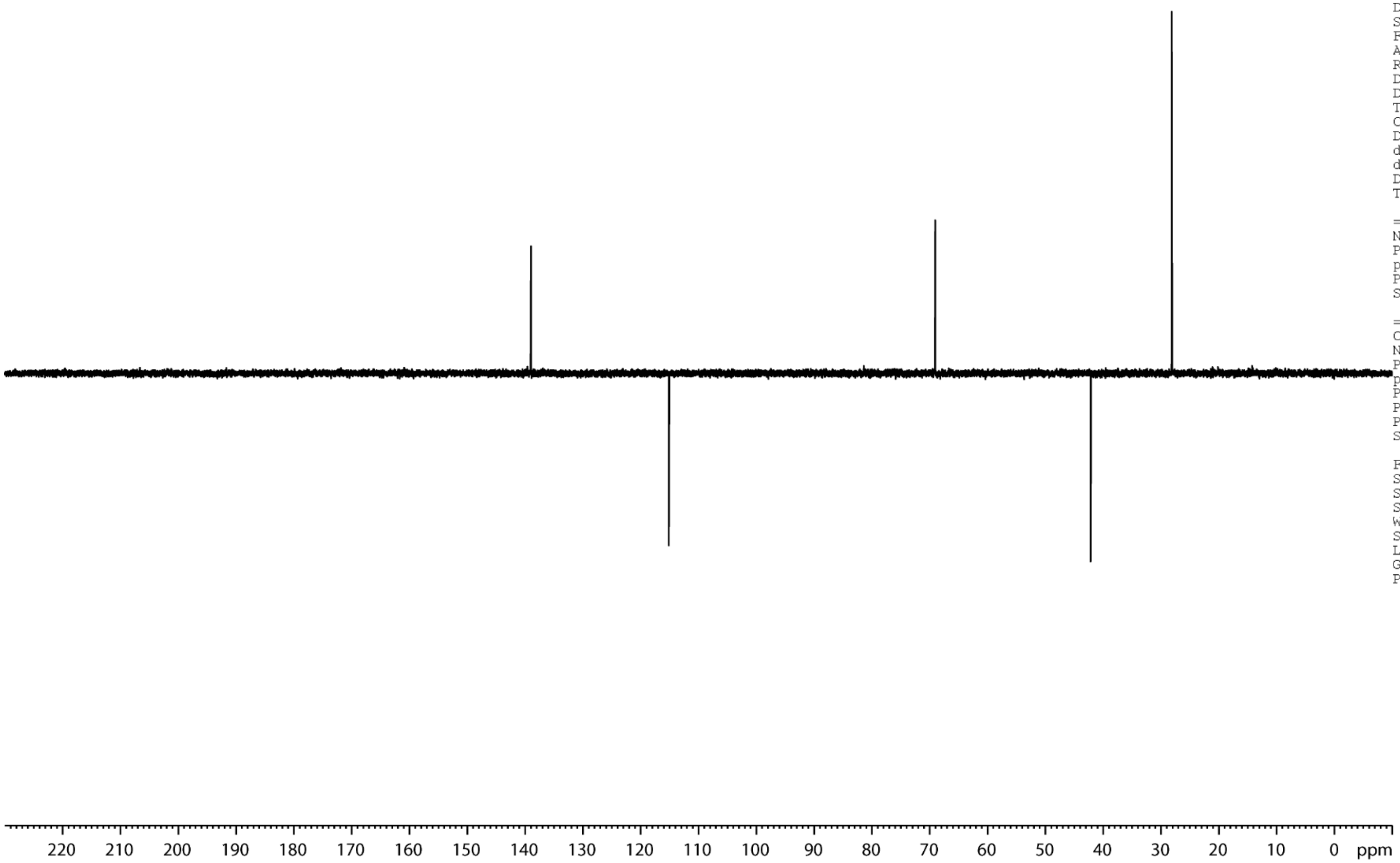

Current Data Parameters
NAME rap120781_od
EXPNO 3
PROCNO 1

F2 Acquisition Parameters
Date_ 20130516
Time 12.14
INSTRUM drx400
PROBHD 5 mm QNP 1H/13
PULPROG dept135
TD 131072
SOLVENT CDCl3
NS 96
DS 4
SWH 26315.789 Hz
FIDRES 0.200774 Hz
AQ 2.4904180 sec
RG 9195.2
DW 19.000 usec
DE 7.00 usec
TE 300.2 K
CNST2 145.0000000
D1 2.00000000 sec
d2 0.00344828 sec
d12 0.00002000 sec
DELTA 0.00001401 sec
TD0 1

======== CHANNEL f1 ========
NUC1 13C
P1 11.00 usec
p2 22.00 usec
PL1 3.00 dB
SFO1 100.5635842 MHz

======== CHANNEL f2 ========
CPDPRG2 waltz16
NUC2 1H
P3 10.00 usec
p4 20.00 usec
PCPD2 80.00 usec
PL2 2.00 dB
PL12 16.06 dB
SFO2 399.8915996 MHz

F2 Processing parameters
SI 65536
SF 100.5524178 MHz
SR 3.20 Hz
WDW EM
SSB 0
LB 1.00 Hz
GB 0
PC 1.40

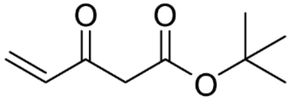

17

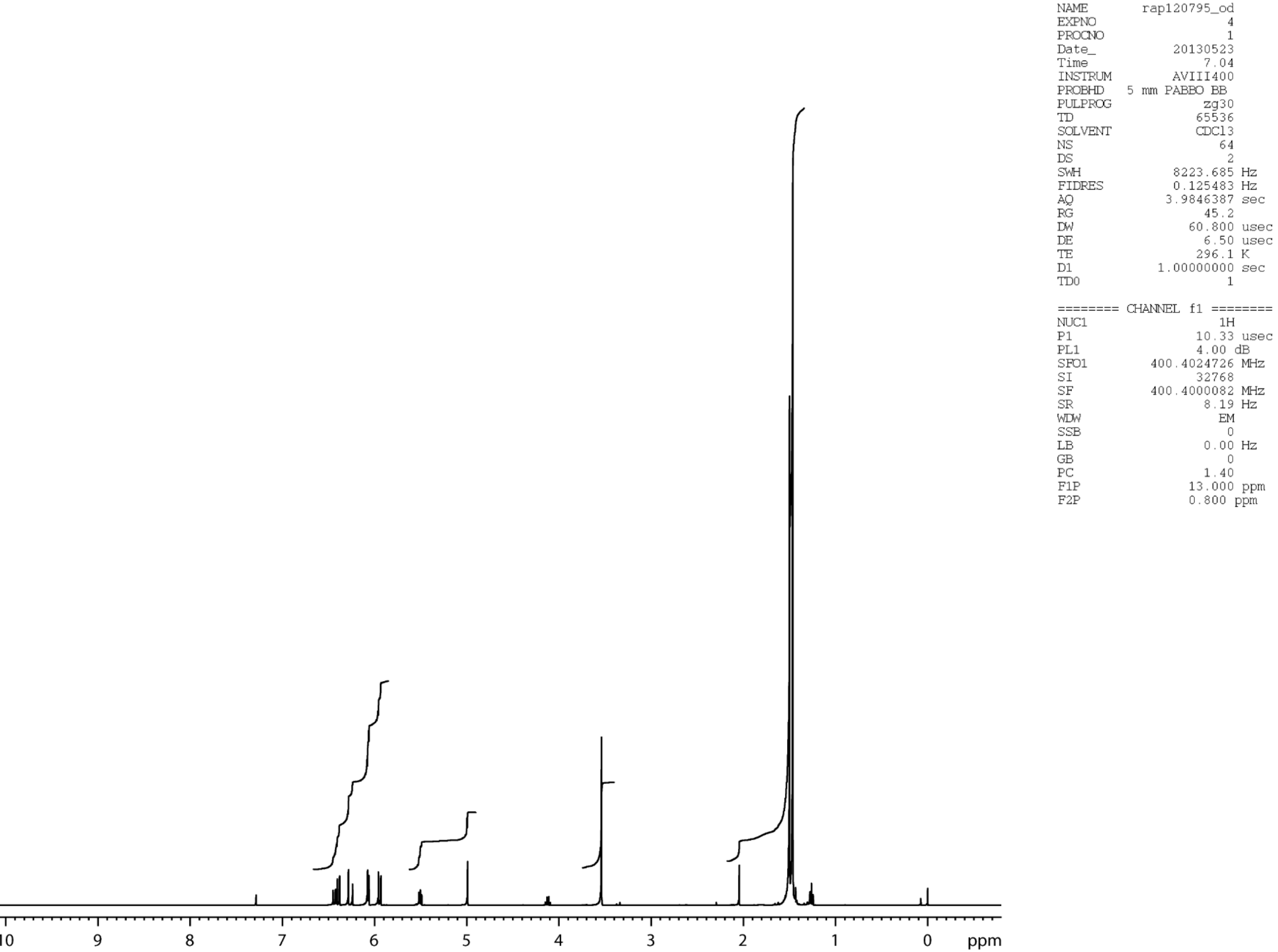

NAME rap120795_od
EXPNO 4
PROCNO 1
Date_ 20130523
Time 7.04
INSTRUM AVIII400
PROBHD 5 mm PABBO BB
PULPROG zg30
TD 65536
SOLVENT CDCl3
NS 64
DS 2
SWH 8223.685 Hz
FIDRES 0.125483 Hz
AQ 3.9846387 sec
RG 45.2
DW 60.800 usec
DE 6.50 usec
TE 296.1 K
D1 1.00000000 sec
TD0 1
======== CHANNEL f1 ========
NUC1 1H
P1 10.33 usec
PL1 4.00 dB
SFO1 400.4024726 MHz
SI 32768
SF 400.4000082 MHz
SR 8.19 Hz
WDW EM
SSB 0
LB 0.00 Hz
GB 0
PC 1.40
F1P 13.000 ppm
F2P 0.800 ppm
12 11 10 9 8 7 6 5 4 3 2 1 0 ppm

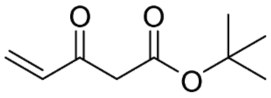

17

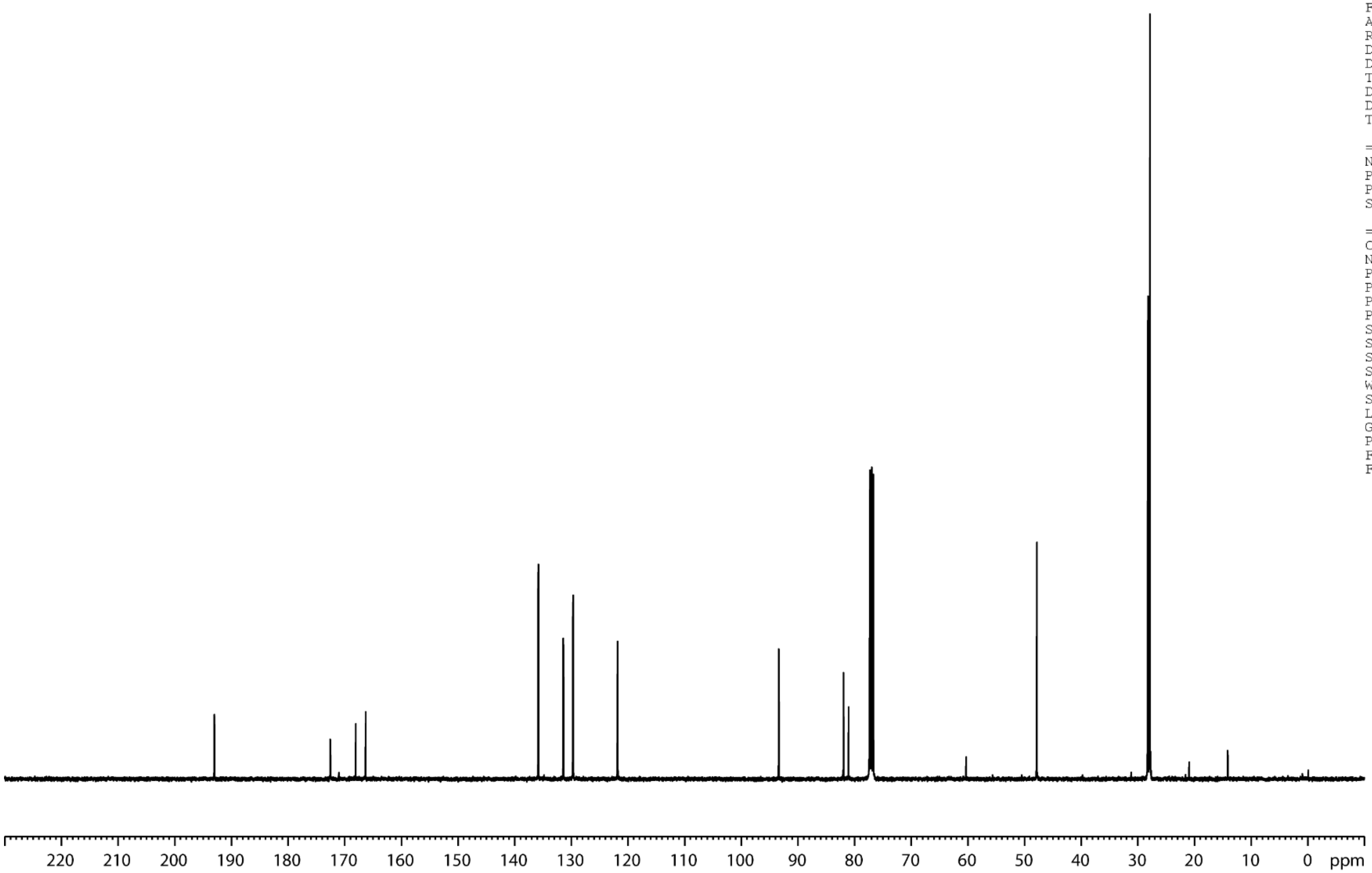


```
NAME        rap120795_od
EXPNO                  2
PROCNO                 1
Date_           20130521
Time               15.00
INSTRUM         AVIII400
PROBHD   5 mm PABBO BB
PULPROG          zgpg30
TD                131072
SOLVENT           CDCl3
NS                  1024
DS                     4
SWH           26315.789 Hz
FIDRES         0.200774 Hz
AQ            2.4904180 sec
RG                  71.8
DW                19.000 usec
DE                  6.50 usec
TE                 298.7 K
D1            2.00000000 sec
D11           0.03000000 sec
TD0                    1

======== CHANNEL f1 ========
NUC1                 13C
P1                  8.50 usec
PL1                 3.00 dB
SFO1         100.6918371 MHz

======== CHANNEL f2 ========
CPDPRG2           waltz16
NUC2                  1H
PCPD2              80.00 usec
PL2                 4.00 dB
PL12               13.78 dB
PL13               14.00 dB
SFO2         400.4016016 MHz
SI                 65536
SF           100.6806651 MHz
SR                  4.09 Hz
WDW                   EM
SSB                    0
LB                  1.00 Hz
GB                     0
PC                  1.40
F1P              230.000 ppm
F2P               10.000 ppm
```

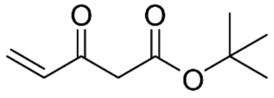

17

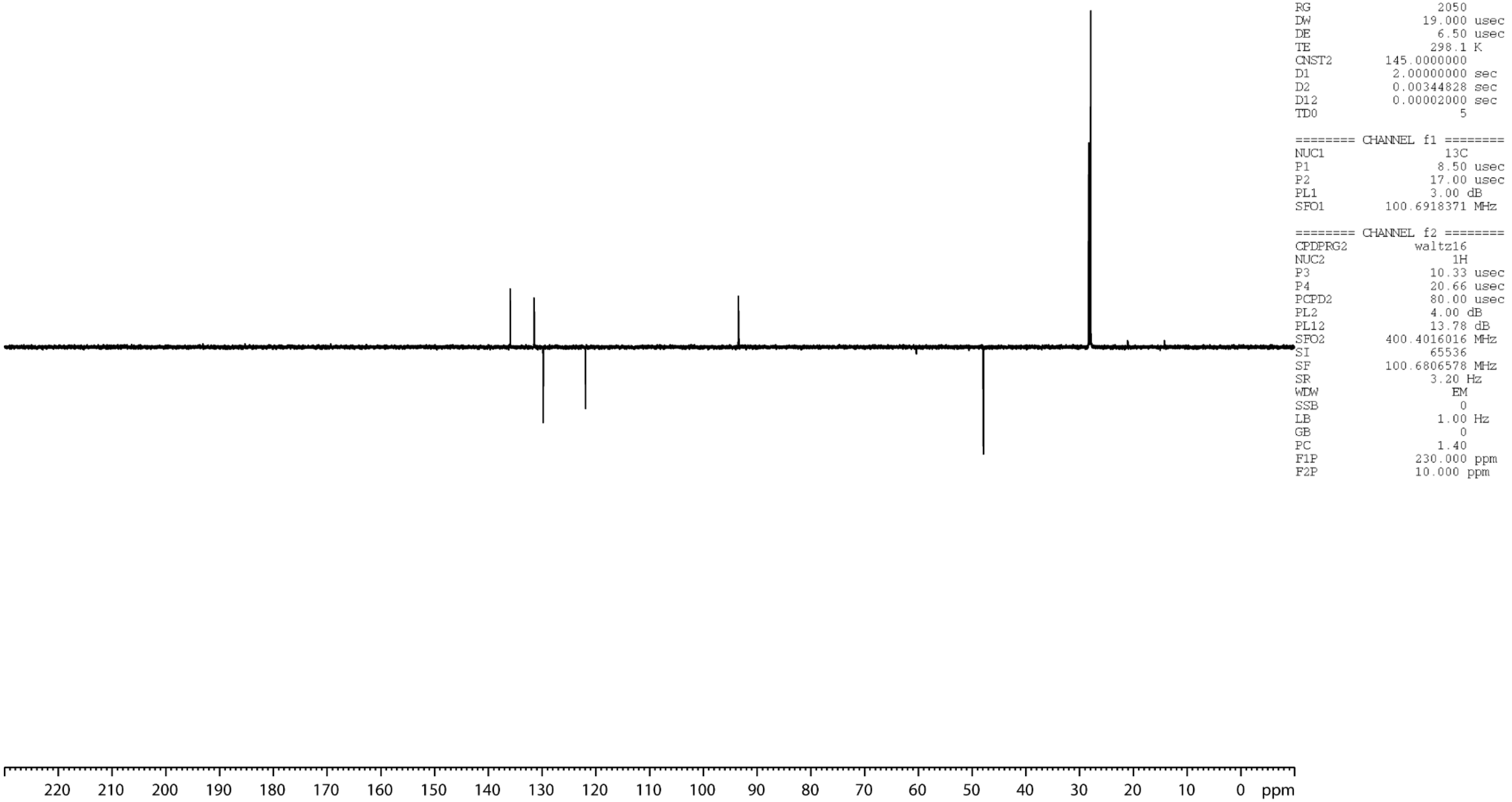
NAME rap120795_od
EXPNO 3
PROCNO 1
Date_ 20130521
Time 15.09
INSTRUM AVIII400
PROBHD 5 mm PABBO BB
PULPROG dept135
TD 131072
SOLVENT CDCl3
NS 96
DS 4
SWH 26315.789 Hz
FIDRES 0.200774 Hz
AQ 2.4904180 sec
RG 2050
DW 19.000 usec
DE 6.50 usec
TE 298.1 K
CNST2 145.0000000
D1 2.00000000 sec
D2 0.00344828 sec
D12 0.00002000 sec
TD0 5
======== CHANNEL f1 ========
NUC1 13C
P1 8.50 usec
P2 17.00 usec
PL1 3.00 dB
SFO1 100.6918371 MHz
======== CHANNEL f2 ========
CPDPRG2 waltz16
NUC2 1H
P3 10.33 usec
P4 20.66 usec
PCPD2 80.00 usec
PL2 4.00 dB
PL12 13.78 dB
SFO2 400.4016016 MHz
SI 65536
SF 100.6806578 MHz
SR 3.20 Hz
WDW EM
SSB 0
LB 1.00 Hz
GB 0
PC 1.40
F1P 230.000 ppm
F2P 10.000 ppm
220 210 200 190 180 170 160 150 140 130 120 110 100 90 80 70 60 50 40 30 20 10 0 ppm

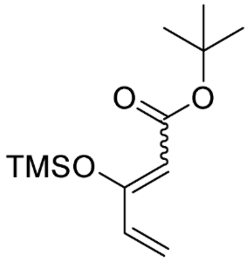

18

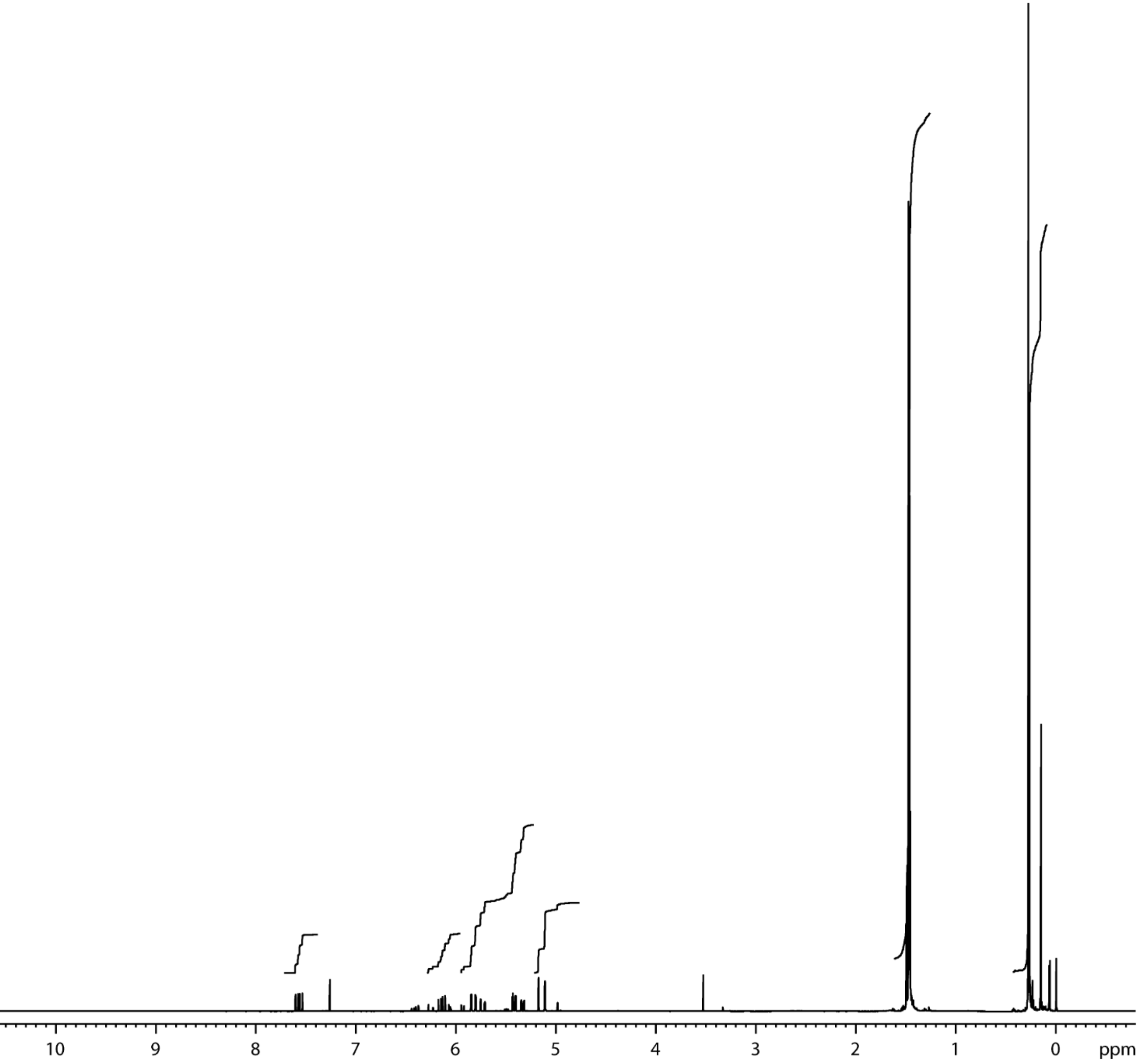

Current Data Parameters
NAME rap120923_od
EXPNO 1
PROCNO 1

F2 Acquisition Parameters
Date_ 20130603
Time 9.39
INSTRUM drx400
PROBHD 5 mm QNP 1H/13
PULPROG zg30
TD 65536
SOLVENT CDCl3
NS 64
DS 2
SWH 8278.146 Hz
FIDRES 0.126314 Hz
AQ 3.9584243 sec
RG 57
DW 60.400 usec
DE 6.00 usec
TE 298.2 K
D1 1.00000000 sec
TD0 1

======== CHANNEL f1 ========
NUC1 1H
P1 10.20 usec
PL1 2.00 dB
SFO1 399.8724688 MHz

F2 Processing parameters
SI 32768
SF 399.8700729 MHz
SR 72.85 Hz
WDW EM
SSB 0
LB 0.00 Hz
GB 0
PC 1.40

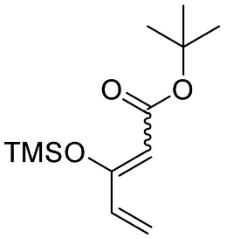

18

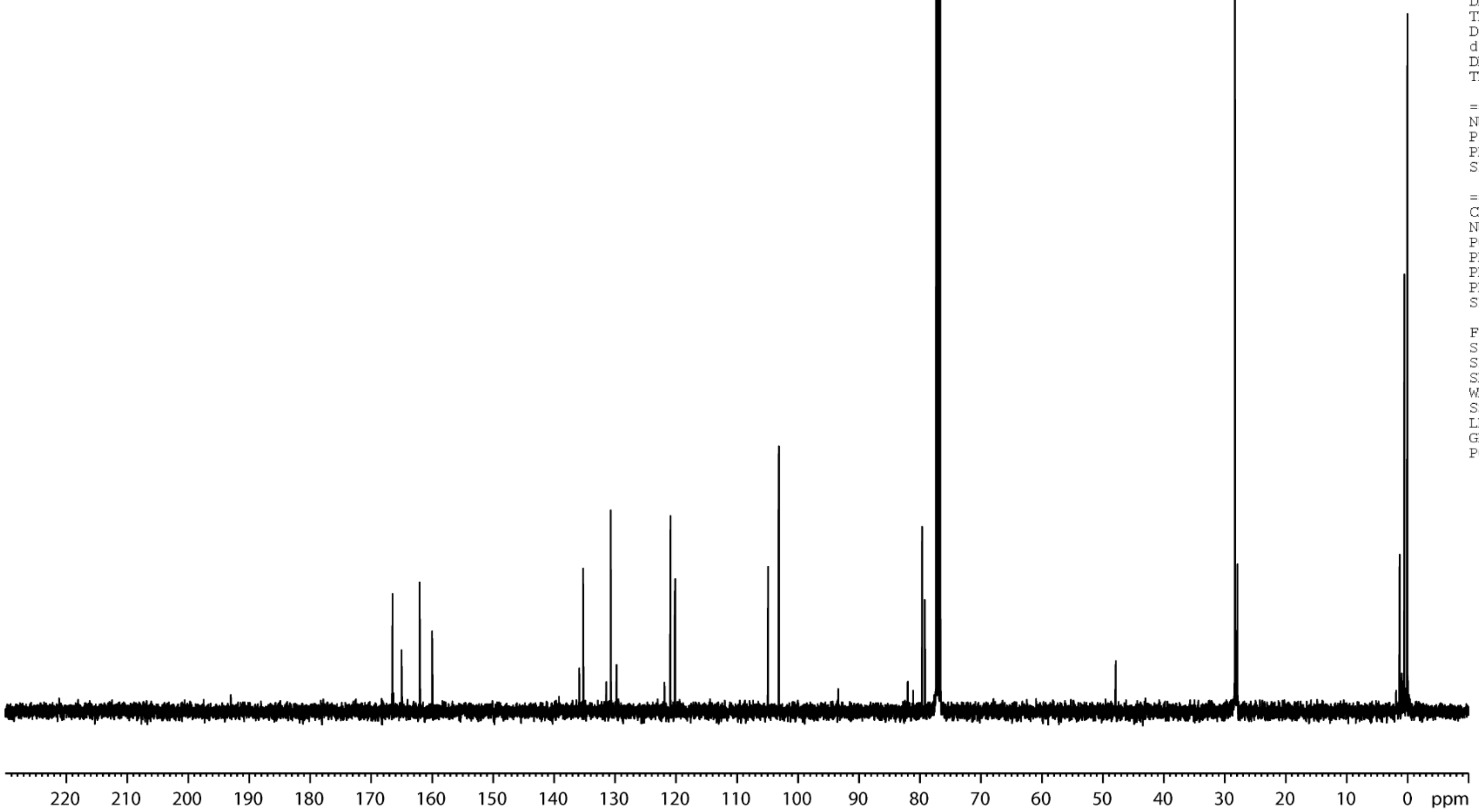


```
Current Data Parameters
NAME        rap120923_od
EXPNO                  2
PROCNO                 1

F2  Acquisition Parameters
Date_           20130603
Time               10.58
INSTRUM           drx400
PROBHD   5 mm QNP 1H/13
PULPROG          zgpg30
TD                131072
SOLVENT            CDCl3
NS                  1024
DS                     4
SWH            26315.789 Hz
FIDRES          0.200774 Hz
AQ             2.4904180 sec
RG                  6502
DW                19.000 usec
DE                  6.00 usec
TE                 299.2 K
D1            2.00000000 sec
d11           0.03000000 sec
DELTA         1.89999998 sec
TD0                    1

======== CHANNEL f1 ========
NUC1                 13C
P1                 11.00 usec
PL1                 3.00 dB
SFO1         100.5585542 MHz

======== CHANNEL f2 ========
CPDPRG2          waltz16
NUC2                  1H
PCPD2              80.00 usec
PL2                 2.00 dB
PL12               16.06 dB
PL13               16.06 dB
SFO2         399.8715995 MHz

F2  Processing parameters
SI                 65536
SF           100.5473940 MHz
SR                  2.01 Hz
WDW                   EM
SSB                    0
LB                  1.00 Hz
GB                     0
PC                  1.40
```

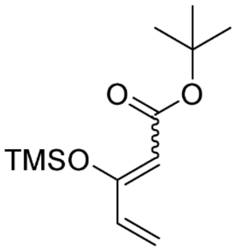

18

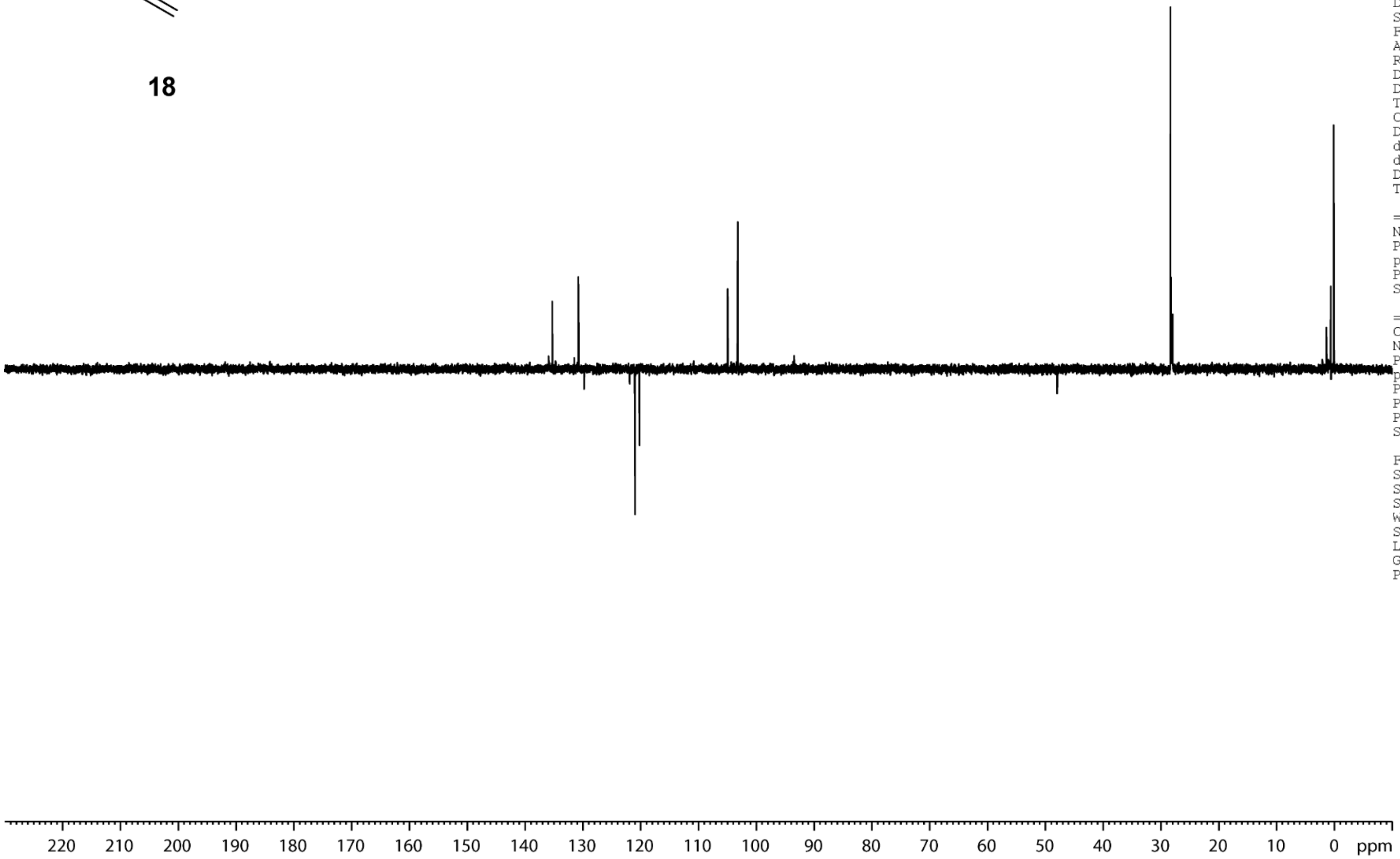


```
Current Data Parameters
NAME        rap120923_od
EXPNO                  3
PROCNO                 1

F2  Acquisition Parameters
Date_           20130603
Time               11.38
INSTRUM           drx400
PROBHD   5 mm QNP 1H/13
PULPROG          dept135
TD                131072
SOLVENT            CDCl3
NS                   512
DS                     4
SWH            26315.789 Hz
FIDRES          0.200774 Hz
AQ             2.4904180 sec
RG                9195.2
DW                19.000 usec
DE                  7.00 usec
TE                 298.2 K
CNST2        145.0000000
D1            2.00000000 sec
d2            0.00344828 sec
d12           0.00002000 sec
DELTA         0.00001401 sec
TD0                    1

======== CHANNEL f1 ========
NUC1                 13C
P1                 11.00 usec
p2                 22.00 usec
PL1                 3.00 dB
SFO1         100.5585542 MHz

======== CHANNEL f2 ========
CPDPRG2          waltz16
NUC2                  1H
P3                 10.00 usec
p4                 20.00 usec
PCPD2              80.00 usec
PL2                 2.00 dB
PL12               16.06 dB
SFO2         399.8715995 MHz

F2  Processing parameters
SI                 65536
SF           100.5473888 MHz
SR                  3.20 Hz
WDW                   EM
SSB                    0
LB                  1.00 Hz
GB                     0
PC                  1.40
```

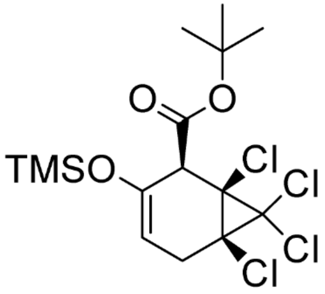

19

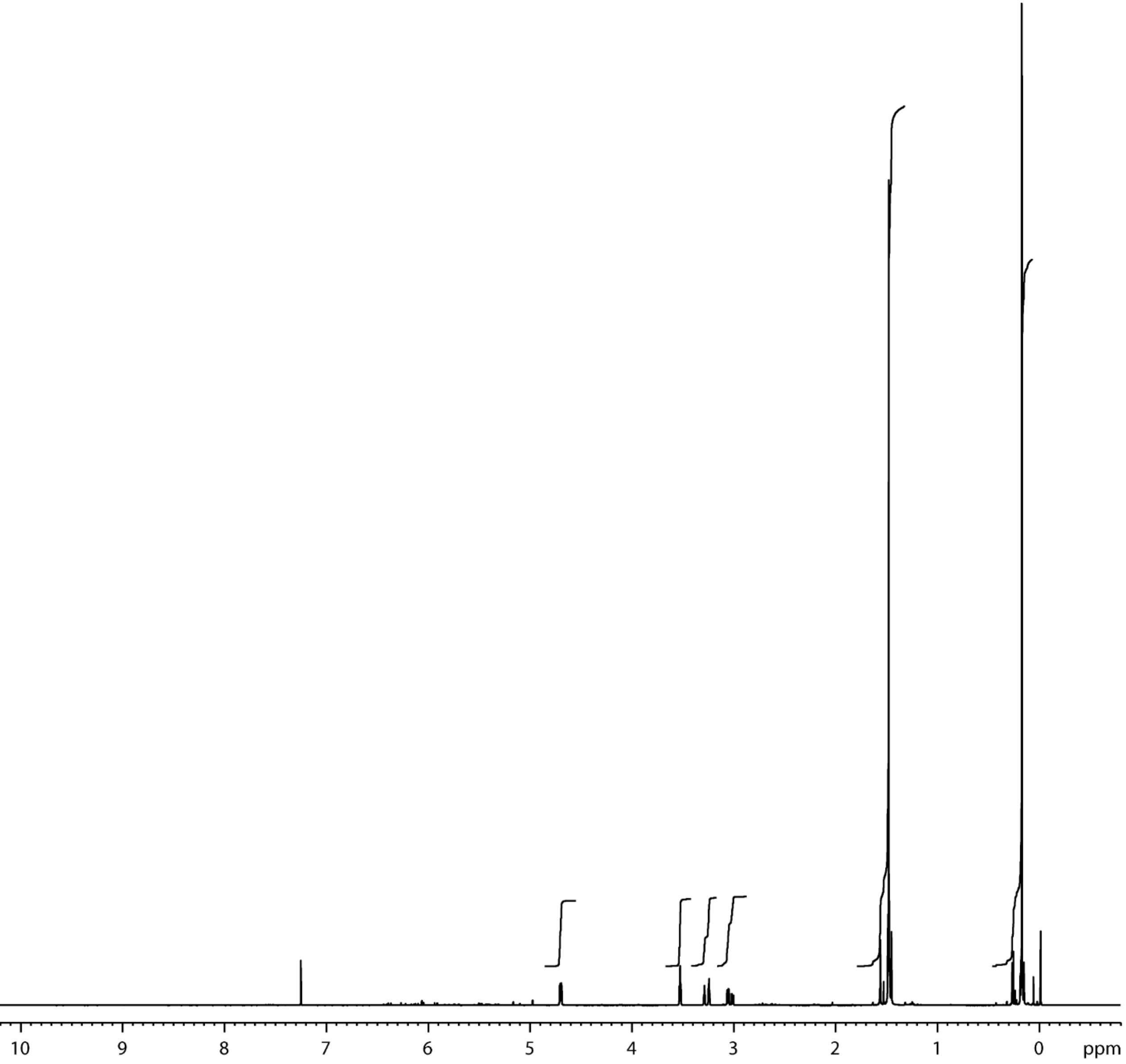


```
Current Data Parameters
NAME        rap120964_od
EXPNO                  1
PROCNO                 1

F2  Acquisition Parameters
Date_           20130606
Time               11.06
INSTRUM           drx400
PROBHD   5 mm QNP 1H/13
PULPROG             zg30
TD                 65536
SOLVENT            CDCl3
NS                    64
DS                     2
SWH             8278.146 Hz
FIDRES          0.126314 Hz
AQ             3.9584243 sec
RG                   114
DW                60.400 usec
DE                  6.00 usec
TE                 298.2 K
D1            1.00000000 sec
TD0                    1

======== CHANNEL f1 ========
NUC1                  1H
P1                 10.20 usec
PL1                 2.00 dB
SFO1         399.8724688 MHz

F2  Processing parameters
SI                 32768
SF           399.8700862 MHz
SR                 86.18 Hz
WDW                   EM
SSB                    0
LB                  0.00 Hz
GB                     0
PC                  1.40
```

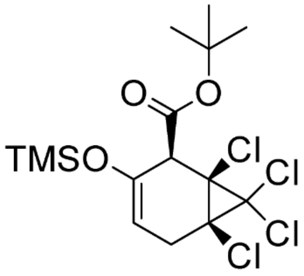

19

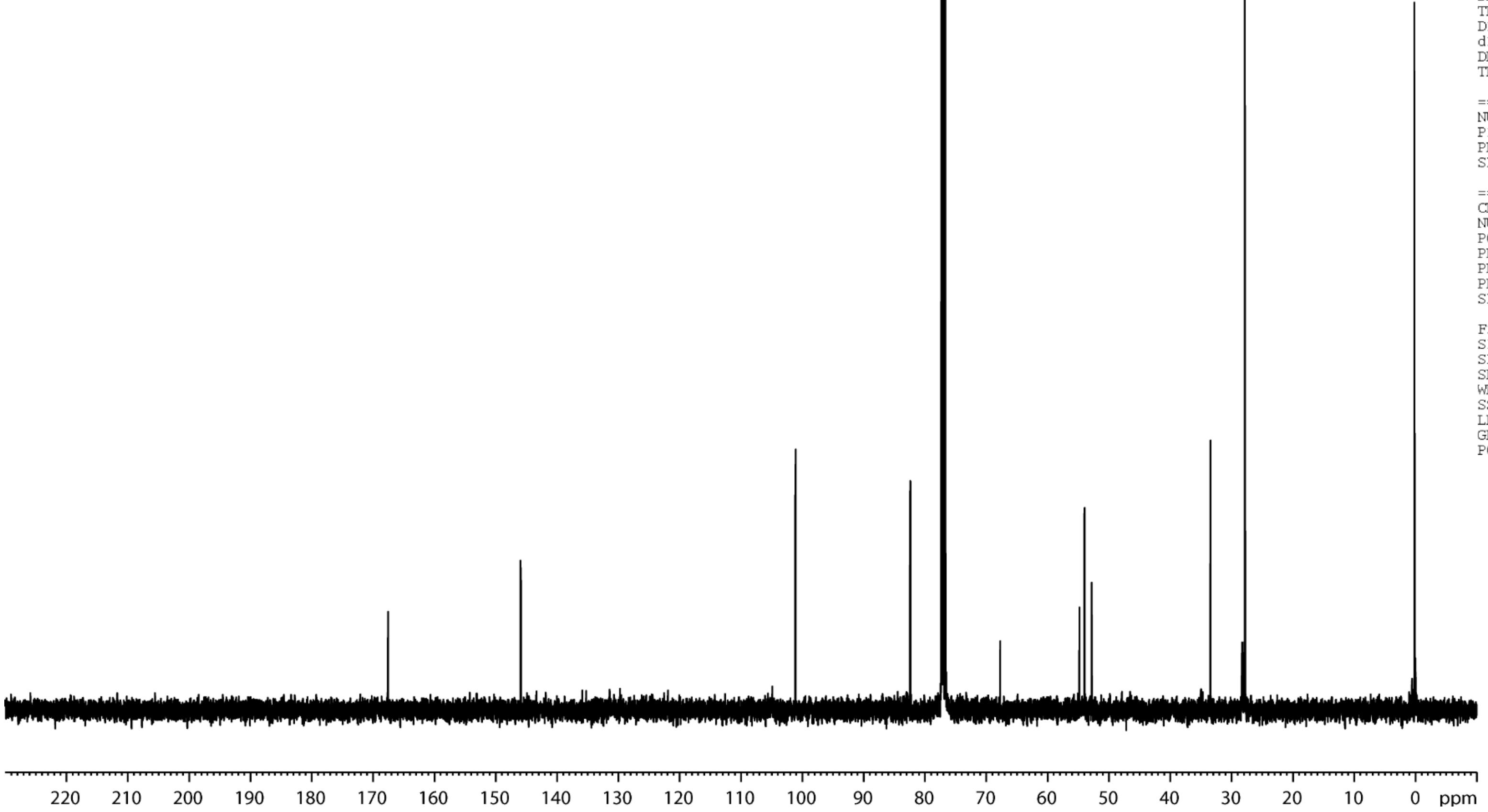

Current Data Parameters
NAME rap120964_od
EXPNO 2
PROCNO 1

F2 Acquisition Parameters
Date_ 20130606
Time 11.27
INSTRUM drx400
PROBHD 5 mm QNP 1H/13
PULPROG zgpg30
TD 131072
SOLVENT CDCl3
NS 1024
DS 4
SWH 26315.789 Hz
FIDRES 0.200774 Hz
AQ 2.4904180 sec
RG 9195.2
DW 19.000 usec
DE 6.00 usec
TE 299.2 K
D1 2.00000000 sec
d11 0.03000000 sec
DELTA 1.89999998 sec
TD0 1

======== CHANNEL f1 ========
NUC1 13C
P1 11.00 usec
PL1 3.00 dB
SFO1 100.5585542 MHz

======== CHANNEL f2 ========
CPDPRG2 waltz16
NUC2 1H
PCPD2 80.00 usec
PL2 2.00 dB
PL12 16.06 dB
PL13 16.06 dB
SFO2 399.8715995 MHz

F2 Processing parameters
SI 65536
SF 100.5473936 MHz
SR 1.61 Hz
WDW EM
SSB 0
LB 1.00 Hz
GB 0
PC 1.40

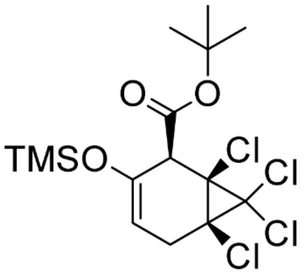

19

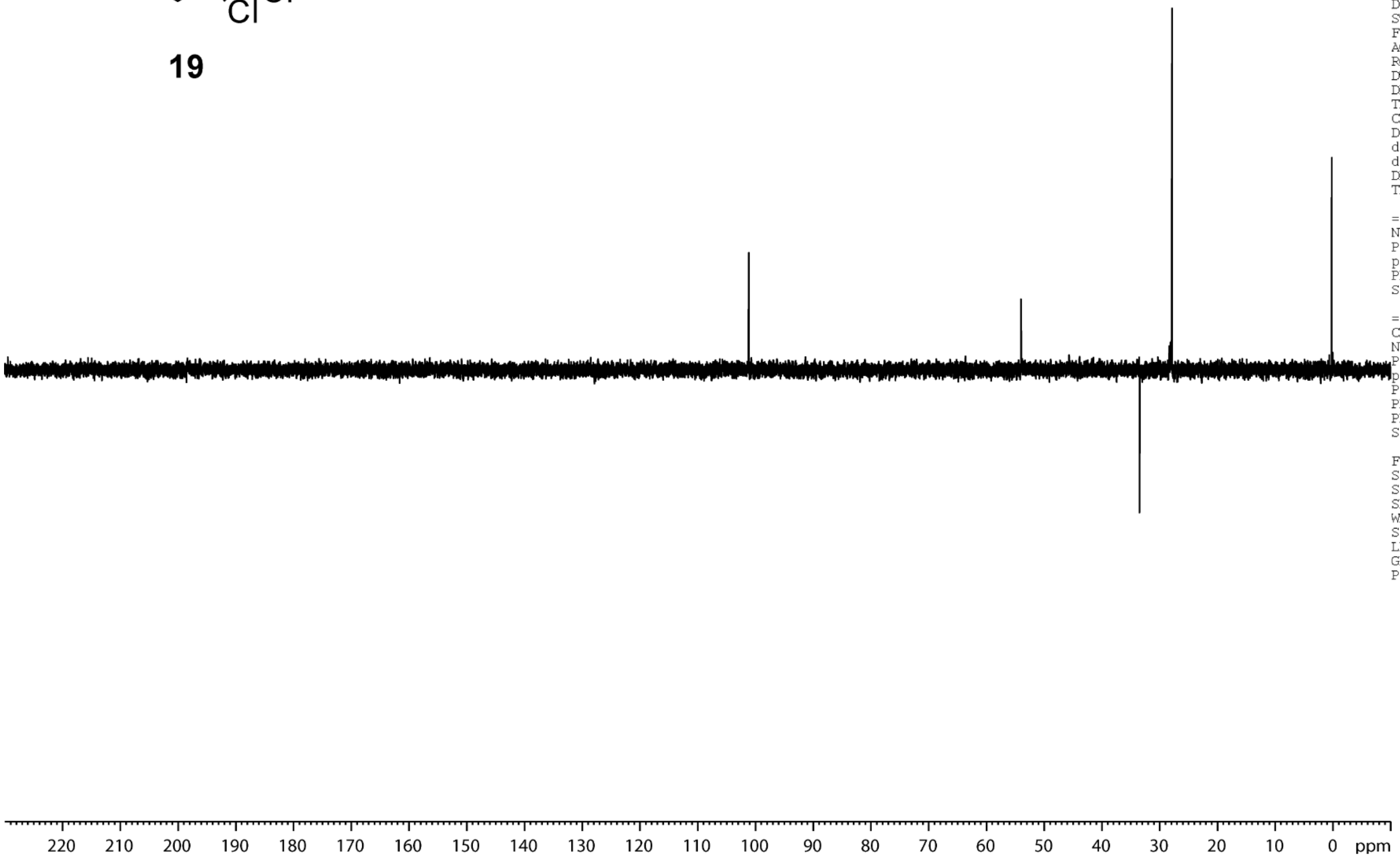

Current Data Parameters
NAME rap120964_od
EXPNO 3
PROCNO 1

F2 Acquisition Parameters
Date_ 20130606
Time 12.33
INSTRUM drx400
PROBHD 5 mm QNP 1H/13
PULPROG dept135
TD 131072
SOLVENT CDCl3
NS 192
DS 4
SWH 26315.789 Hz
FIDRES 0.200774 Hz
AQ 2.4904180 sec
RG 8192
DW 19.000 usec
DE 7.00 usec
TE 299.2 K
CNST2 145.0000000
D1 2.00000000 sec
d2 0.00344828 sec
d12 0.00002000 sec
DELTA 0.00001401 sec
TD0 1

======== CHANNEL f1 ========
NUC1 13C
P1 11.00 usec
p2 22.00 usec
PL1 3.00 dB
SFO1 100.5585542 MHz

======== CHANNEL f2 ========
CPDPRG2 waltz16
NUC2 1H
P3 10.00 usec
p4 20.00 usec
PCPD2 80.00 usec
PL2 2.00 dB
PL12 16.06 dB
SFO2 399.8715995 MHz

F2 Processing parameters
SI 65536
SF 100.5473888 MHz
SR 3.20 Hz
WDW EM
SSB 0
LB 1.00 Hz
GB 0
PC 1.40

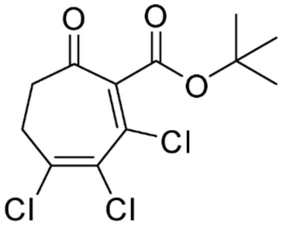

20

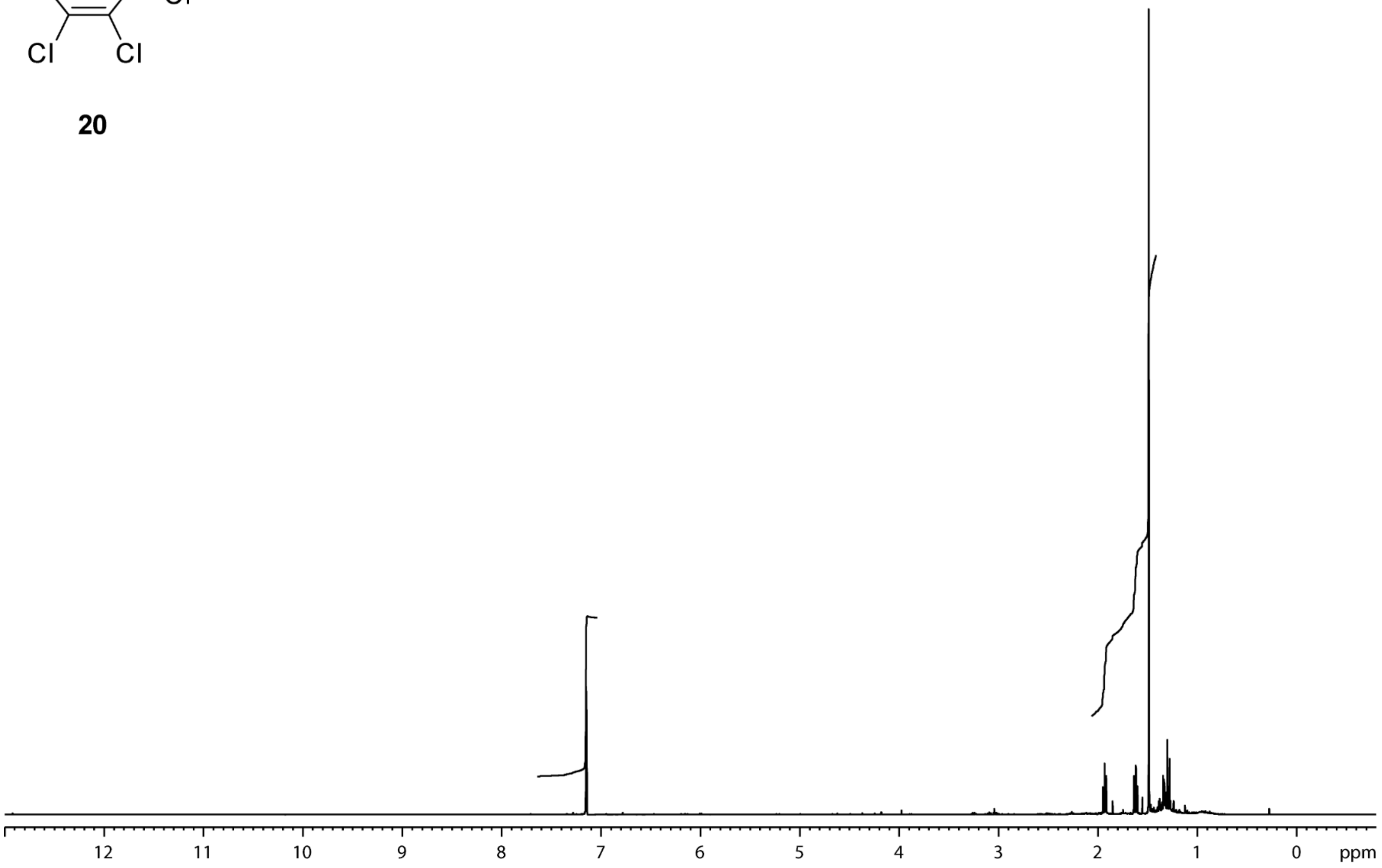

Current Data Parameters
NAME rap122191_od
EXPNO 1
PROCNO 1

F2 Acquisition Parameters
Date_ 20131009
Time 12.59
INSTRUM drx400
PROBHD 5 mm QNP 1H/13
PULPROG zg30
TD 65536
SOLVENT C6D6
NS 64
DS 2
SWH 8278.146 Hz
FIDRES 0.126314 Hz
AQ 3.9584243 sec
RG 101.6
DW 60.400 usec
DE 6.00 usec
TE 297.2 K
D1 1.00000000 sec
TD0 1

======== CHANNEL f1 ========
NUC1 1H
P1 10.20 usec
PL1 2.00 dB
SFO1 399.8524687 MHz

F2 Processing parameters
SI 32768
SF 399.8500407 MHz
SR 40.68 Hz
WDW EM
SSB 0
LB 0.00 Hz
GB 0
PC 1.40

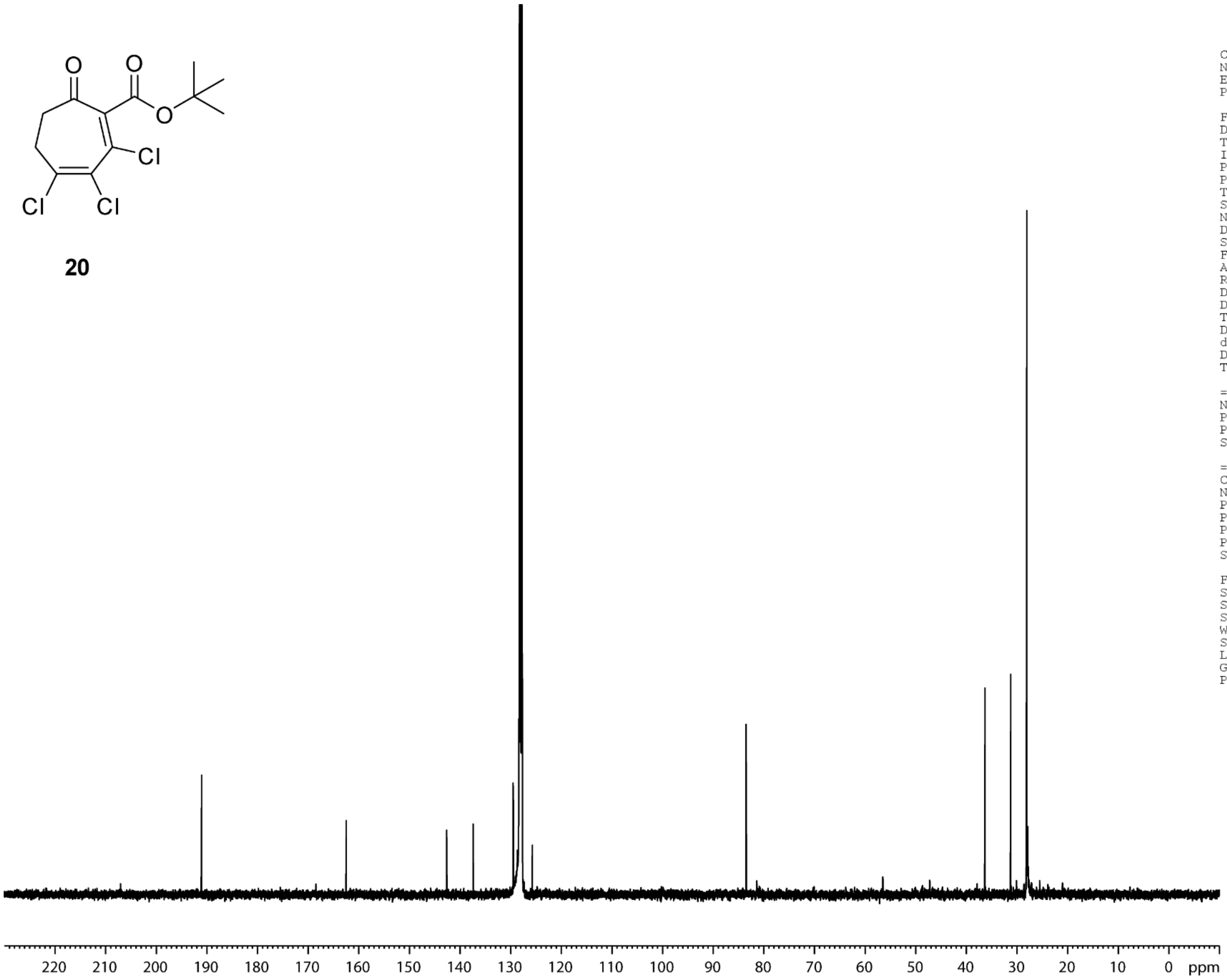

20

```
Current Data Parameters
NAME        rap122191_od
EXPNO                  2
PROCNO                 1

F2  Acquisition Parameters
Date_           20131009
Time               16.53
INSTRUM           drx400
PROBHD   5 mm QNP 1H/13
PULPROG          zgpg30
TD                131072
SOLVENT             C6D6
NS                  3072
DS                     4
SWH           26315.789 Hz
FIDRES         0.200774 Hz
AQ            2.4904180 sec
RG                7298.2
DW                19.000 usec
DE                  6.00 usec
TE                 298.2 K
D1            2.00000000 sec
d11           0.03000000 sec
DELTA         1.89999998 sec
TD0                    1

======== CHANNEL f1 ========
NUC1                 13C
P1                 11.00 usec
PL1                 3.00 dB
SFO1         100.5535241 MHz

======== CHANNEL f2 ========
CPDPRG2          waltz16
NUC2                  1H
PCPD2              80.00 usec
PL2                 2.00 dB
PL12               16.06 dB
PL13               16.06 dB
SFO2         399.8515994 MHz

F2  Processing parameters
SI                 65536
SF           100.5423266 MHz
SR                 36.36 Hz
WDW                   EM
SSB                    0
LB                  1.00 Hz
GB                     0
PC                  1.40
```

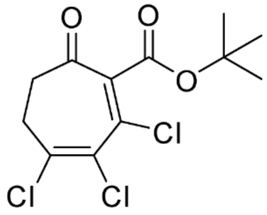

20

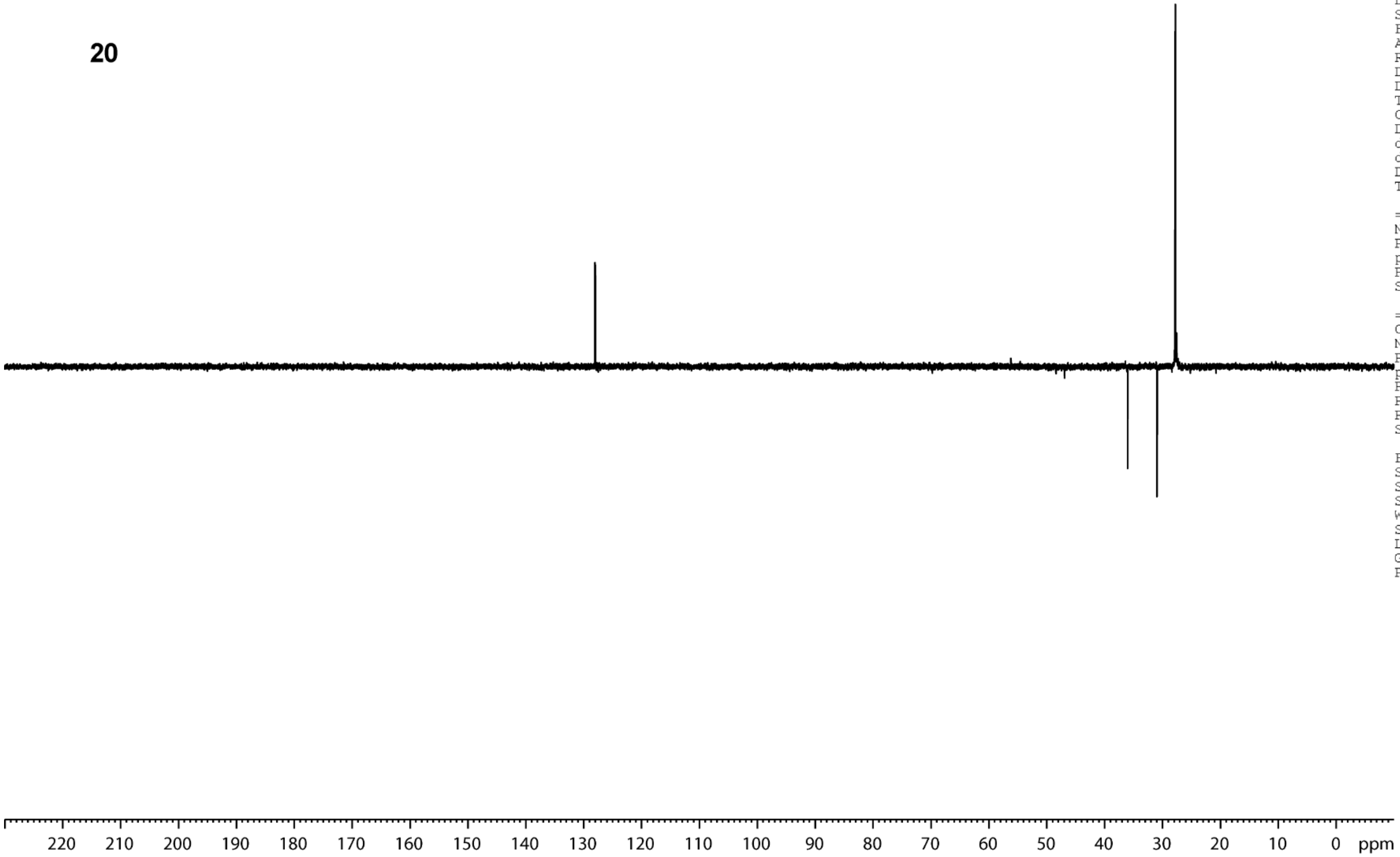


```
Current Data Parameters
NAME       rap122191_od
EXPNO                 3
PROCNO                1

F2  Acquisition Parameters
Date_          20131009
Time              18.12
INSTRUM          drx400
PROBHD   5 mm QNP 1H/13
PULPROG         dept135
TD               131072
SOLVENT            C6D6
NS                 1024
DS                    4
SWH          26315.789 Hz
FIDRES        0.200774 Hz
AQ           2.4904180 sec
RG               9195.2
DW               19.000 usec
DE                 7.00 usec
TE                298.2 K
CNST2       145.0000000
D1           2.00000000 sec
d2           0.00344828 sec
d12          0.00002000 sec
DELTA        0.00001401 sec
TD0                   1

======== CHANNEL f1 ========
NUC1                13C
P1                11.00 usec
p2                22.00 usec
PL1                3.00 dB
SFO1        100.5535241 MHz

======== CHANNEL f2 ========
CPDPRG2         waltz16
NUC2                 1H
P3                10.00 usec
p4                20.00 usec
PCPD2             80.00 usec
PL2                2.00 dB
PL12              16.06 dB
SFO2        399.8515994 MHz

F2  Processing parameters
SI                65536
SF          100.5423561 MHz
SR                 6.92 Hz
WDW                  EM
SSB                   0
LB                 1.00 Hz
GB                    0
PC                 1.40
```

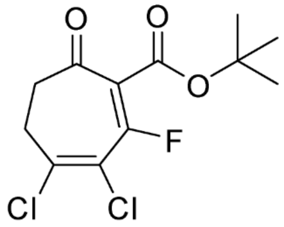

21

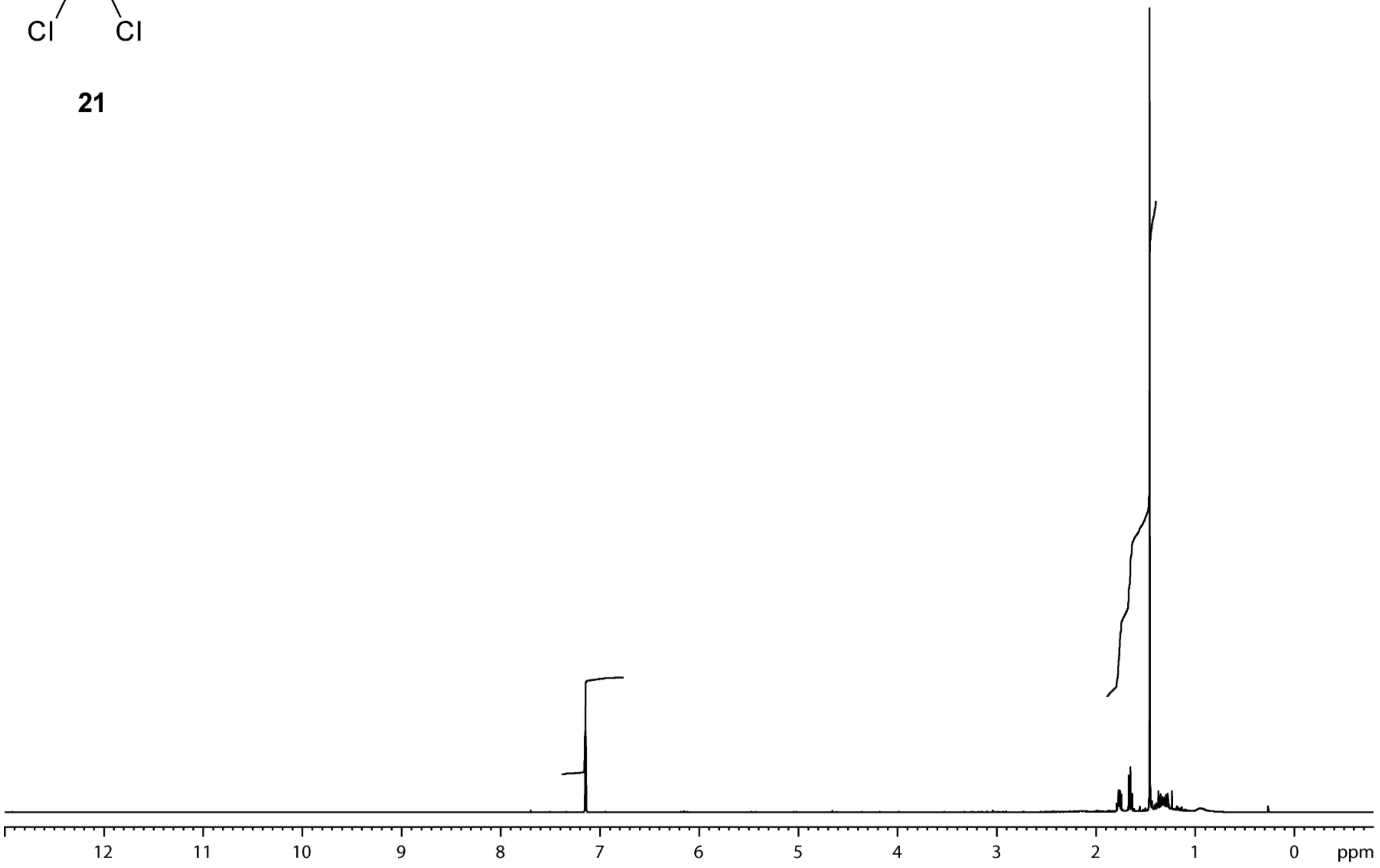


```
Current Data Parameters
NAME       rap122192_od
EXPNO                 1
PROCNO                1

F2  Acquisition Parameters
Date_          20131009
Time              18.22
INSTRUM          drx400
PROBHD   5 mm QNP 1H/13
PULPROG            zg30
TD                65536
SOLVENT            C6D6
NS                   64
DS                    2
SWH            8278.146 Hz
FIDRES         0.126314 Hz
AQ            3.9584243 sec
RG                 71.8
DW               60.400 usec
DE                 6.00 usec
TE                297.2 K
D1           1.00000000 sec
TD0                   1

======== CHANNEL f1 ========
NUC1                 1H
P1                10.20 usec
PL1                2.00 dB
SFO1        399.8524687 MHz

F2  Processing parameters
SI                32768
SF          399.8500000 MHz
SR                 0.00 Hz
WDW                  EM
SSB                   0
LB                 0.00 Hz
GB                    0
PC                 1.40
```

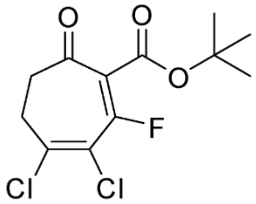
O
O
O
F
Cl
Cl

21

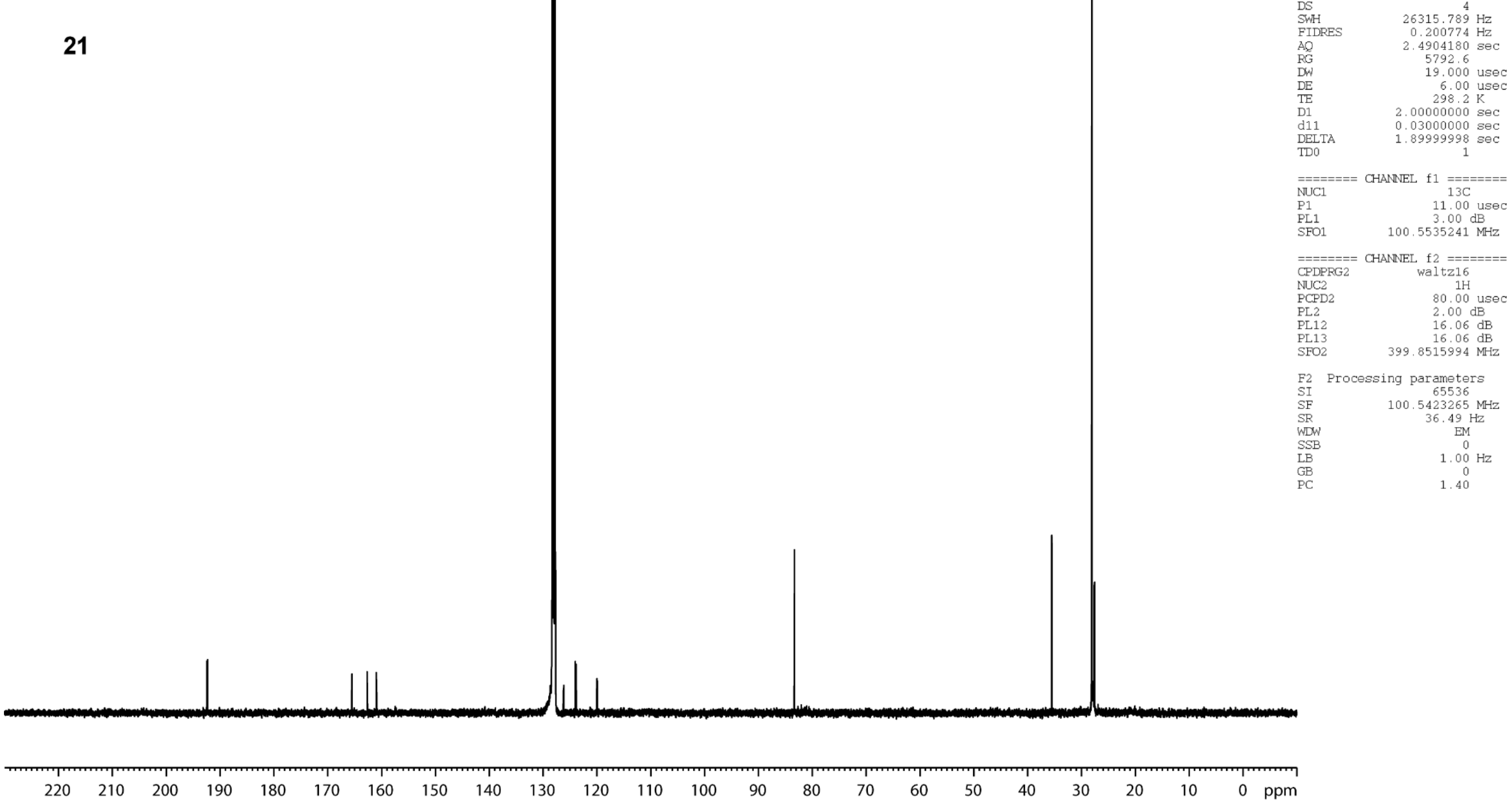
Current Data Parameters
NAME rap122192_od
EXPNO 2
PROCNO 1
F2 Acquisition Parameters
Date_ 20131009
Time 20.59
INSTRUM drx400
PROBHD 5 mm QNP 1H/13
PULPROG zgpg30
TD 131072
SOLVENT C6D6
NS 2048
DS 4
SWH 26315.789 Hz
FIDRES 0.200774 Hz
AQ 2.4904180 sec
RG 5792.6
DW 19.000 usec
DE 6.00 usec
TE 298.2 K
D1 2.00000000 sec
d11 0.03000000 sec
DELTA 1.89999998 sec
TD0 1
======== CHANNEL f1 ========
NUC1 13C
P1 11.00 usec
PL1 3.00 dB
SFO1 100.5535241 MHz
======== CHANNEL f2 ========
CPDPRG2 waltz16
NUC2 1H
PCPD2 80.00 usec
PL2 2.00 dB
PL12 16.06 dB
PL13 16.06 dB
SFO2 399.8515994 MHz
F2 Processing parameters
SI 65536
SF 100.5423265 MHz
SR 36.49 Hz
WDW EM
SSB 0
LB 1.00 Hz
GB 0
PC 1.40
220 210 200 190 180 170 160 150 140 130 120 110 100 90 80 70 60 50 40 30 20 10 0 ppm

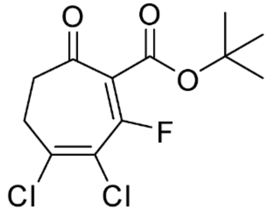

21

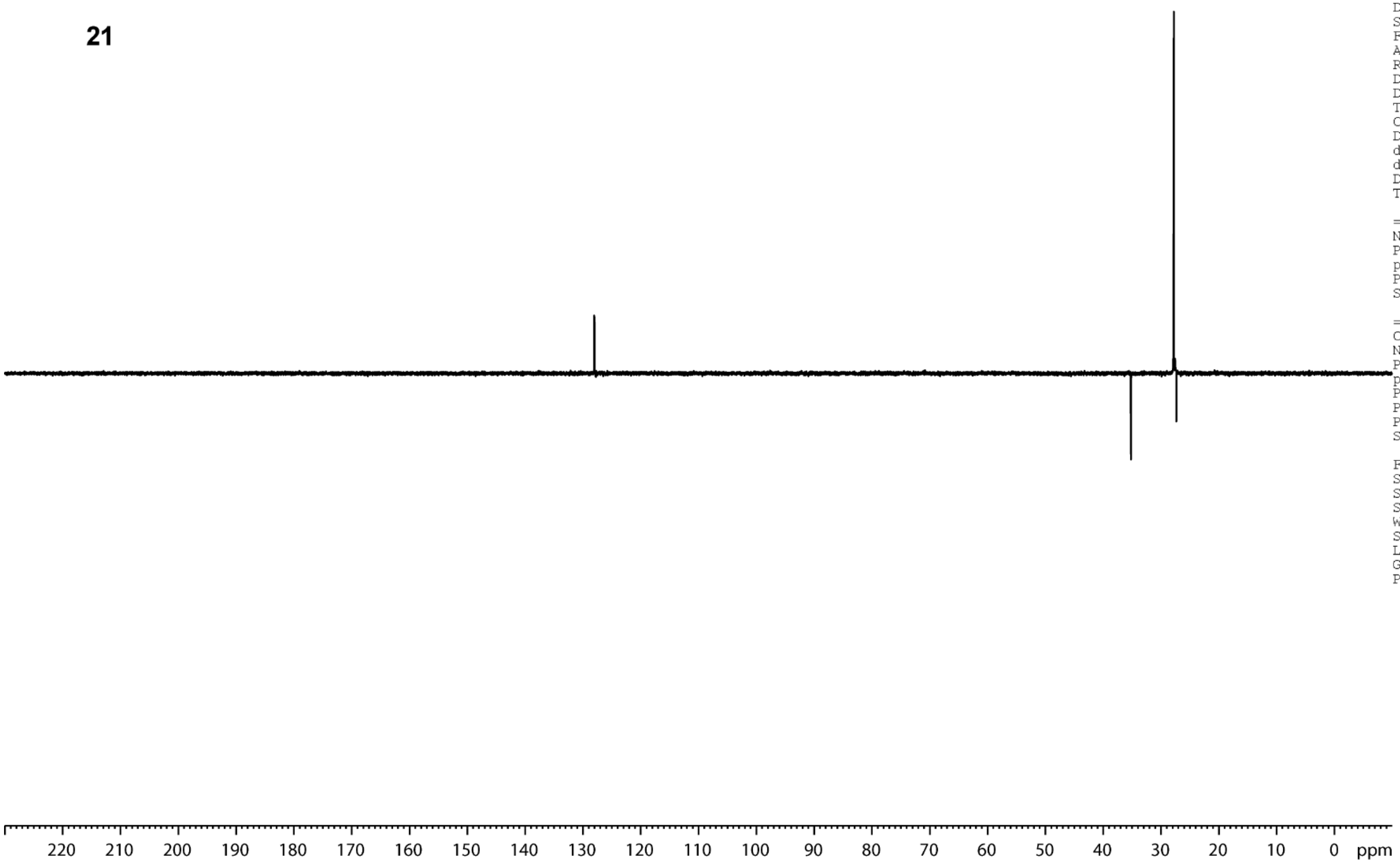


```
Current Data Parameters
NAME        rap122192_od
EXPNO                  3
PROCNO                 1

F2  Acquisition Parameters
Date_           20131009
Time               22.17
INSTRUM           drx400
PROBHD    5 mm QNP 1H/13
PULPROG          dept135
TD                131072
SOLVENT             C6D6
NS                  1024
DS                     4
SWH            26315.789 Hz
FIDRES          0.200774 Hz
AQ             2.4904180 sec
RG                9195.2
DW                19.000 usec
DE                  7.00 usec
TE                 297.2 K
CNST2        145.0000000
D1            2.00000000 sec
d2            0.00344828 sec
d12           0.00002000 sec
DELTA         0.00001401 sec
TD0                    1

======== CHANNEL f1 ========
NUC1                 13C
P1                 11.00 usec
p2                 22.00 usec
PL1                 3.00 dB
SFO1         100.5535241 MHz

======== CHANNEL f2 ========
CPDPRG2          waltz16
NUC2                  1H
P3                 10.00 usec
p4                 20.00 usec
PCPD2              80.00 usec
PL2                 2.00 dB
PL12               16.06 dB
SFO2         399.8515994 MHz

F2  Processing parameters
SI                 65536
SF           100.5423561 MHz
SR                  6.87 Hz
WDW                   EM
SSB                    0
LB                  1.00 Hz
GB                     0
PC                  1.40
```

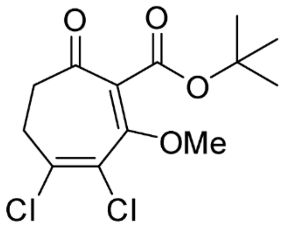

22

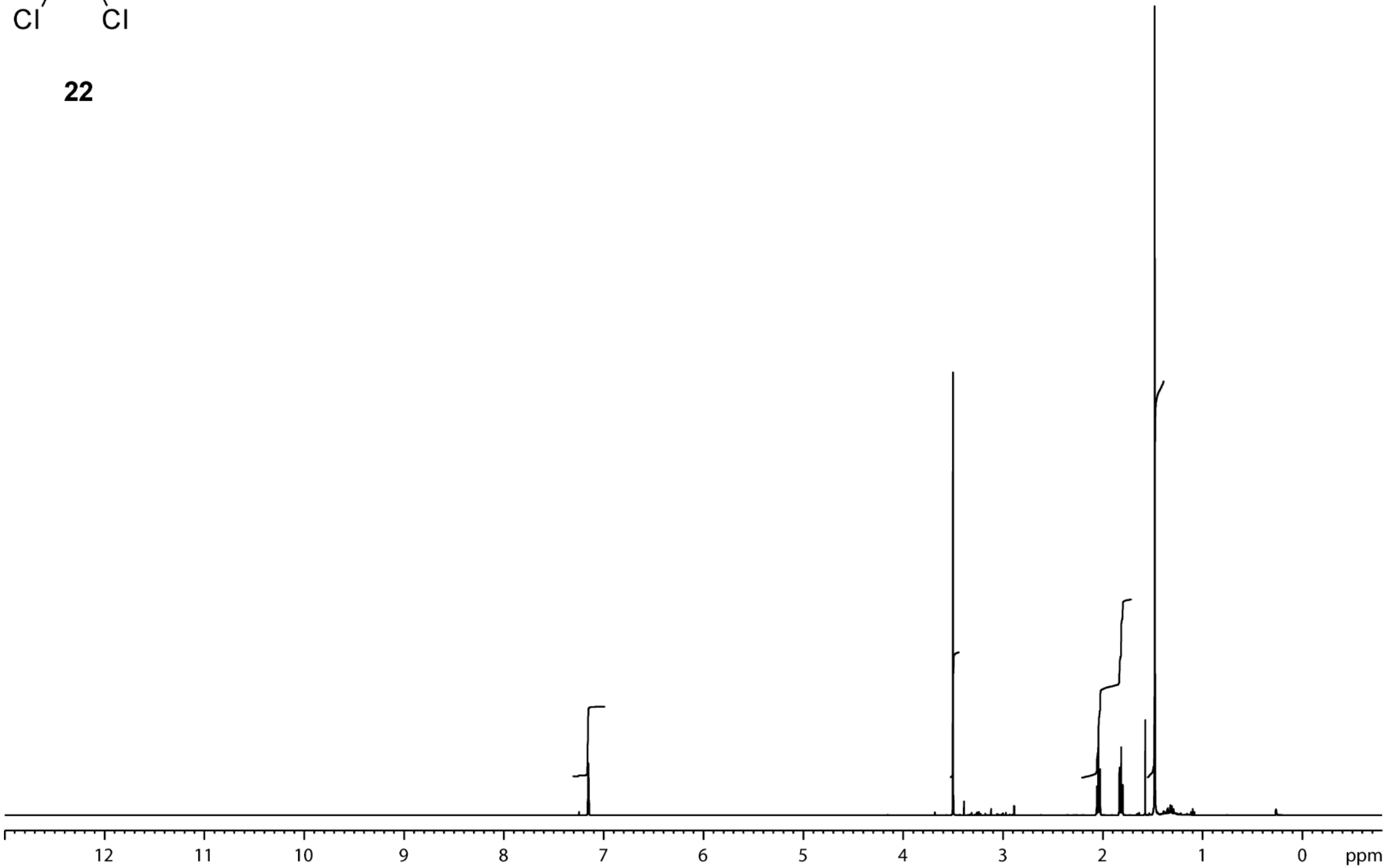

NAME	rap122188_od
EXPNO	1
PROCNO	1
Date_	20131009
Time	22.39
INSTRUM	AVIII400
PROBHD	5 mm PABBO BB
PULPROG	zg30
TD	65536
SOLVENT	C6D6
NS	64
DS	2
SWH	8223.685 Hz
FIDRES	0.125483 Hz
AQ	3.9846387 sec
RG	45.2
DW	60.800 usec
DE	6.50 usec
TE	300.0 K
D1	1.00000000 sec
TD0	1

======== CHANNEL f1 ========

NUC1	1H
P1	10.33 usec
PL1	4.00 dB
SFO1	400.4024726 MHz
SI	32768
SF	400.4000945 MHz
SR	94.50 Hz
WDW	EM
SSB	0
LB	0.00 Hz
GB	0
PC	1.40
F1P	13.000 ppm
F2P	0.800 ppm

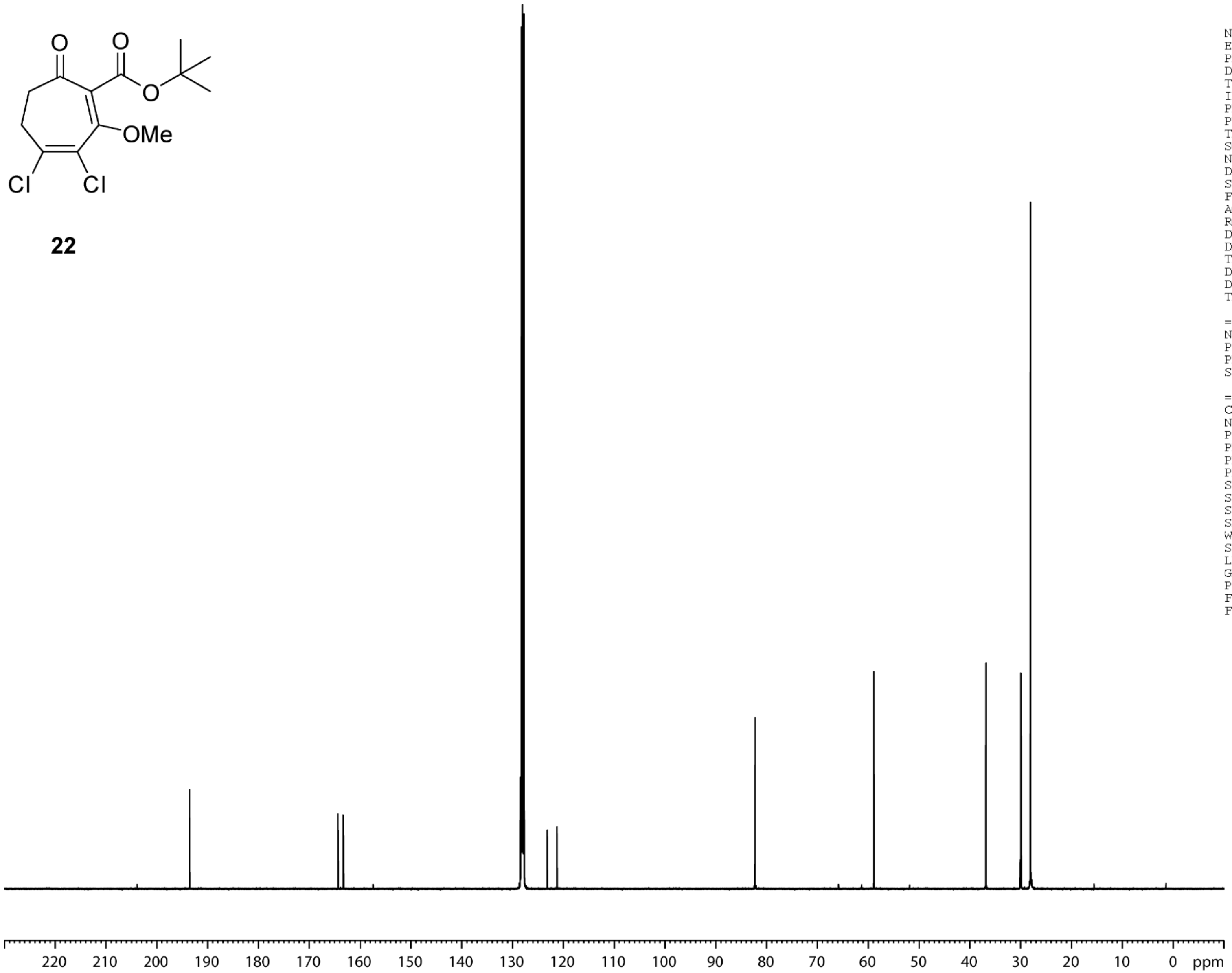

NAME	rap122188_od
EXPNO	2
PROCNO	1
Date_	20131010
Time	1.16
INSTRUM	AVIII400
PROBHD	5 mm PABBO BB
PULPROG	zgpg30
TD	131072
SOLVENT	C6D6
NS	2048
DS	4
SWH	26315.789 Hz
FIDRES	0.200774 Hz
AQ	2.4904180 sec
RG	101
DW	19.000 usec
DE	6.50 usec
TE	300.0 K
D1	2.00000000 sec
D11	0.03000000 sec
TD0	1
======== CHANNEL f1 ========	
NUC1	13C
P1	8.50 usec
PL1	3.00 dB
SFO1	100.6918371 MHz
======== CHANNEL f2 ========	
CPDPRG2	waltz16
NUC2	1H
PCPD2	80.00 usec
PL2	4.00 dB
PL12	13.78 dB
PL13	14.00 dB
SFO2	400.4016016 MHz
SI	65536
SF	100.6806254 MHz
SR	35.62 Hz
WDW	EM
SSB	0
LB	1.00 Hz
GB	0
PC	1.40
F1P	230.000 ppm
F2P	10.000 ppm

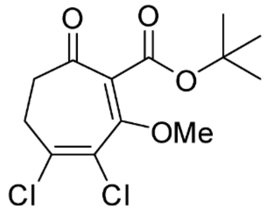

22

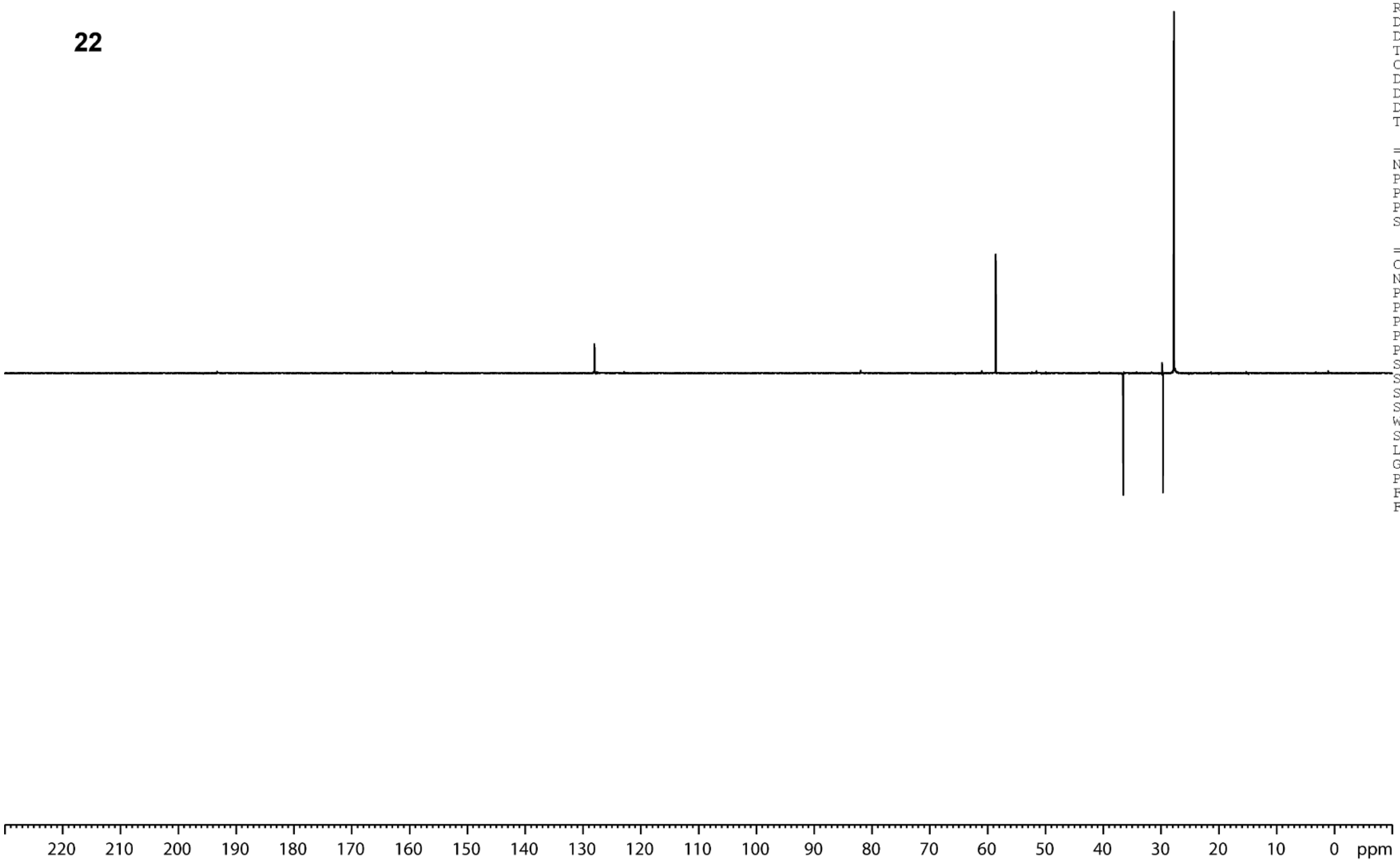


```
NAME        rap122188_od
EXPNO                  3
PROCNO                 1
Date_           20131010
Time                2.36
INSTRUM         AVIII400
PROBHD   5 mm PABBO BB
PULPROG          dept135
TD                131072
SOLVENT             C6D6
NS                  1024
DS                     4
SWH            26315.789 Hz
FIDRES          0.200774 Hz
AQ             2.4904180 sec
RG                  2050
DW                19.000 usec
DE                  6.50 usec
TE                 300.0 K
CNST2        145.0000000
D1            2.00000000 sec
D2            0.00344828 sec
D12           0.00002000 sec
TD0                    1

======== CHANNEL f1 ========
NUC1                 13C
P1                  8.50 usec
P2                 17.00 usec
PL1                 3.00 dB
SFO1         100.6919063 MHz

======== CHANNEL f2 ========
CPDPRG2          waltz16
NUC2                  1H
P3                 10.33 usec
P4                 20.66 usec
PCPD2              80.00 usec
PL2                 4.00 dB
PL12               13.78 dB
SFO2         400.4016016 MHz
SI                 65536
SF           100.6806551 MHz
SR                  5.86 Hz
WDW                   EM
SSB                    0
LB                  1.00 Hz
GB                     0
PC                  1.40
F1P              230.000 ppm
F2P               10.000 ppm
```

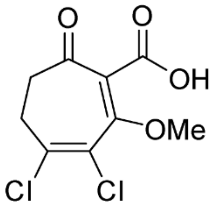
O
O
OH
OMe
Cl
Cl

23

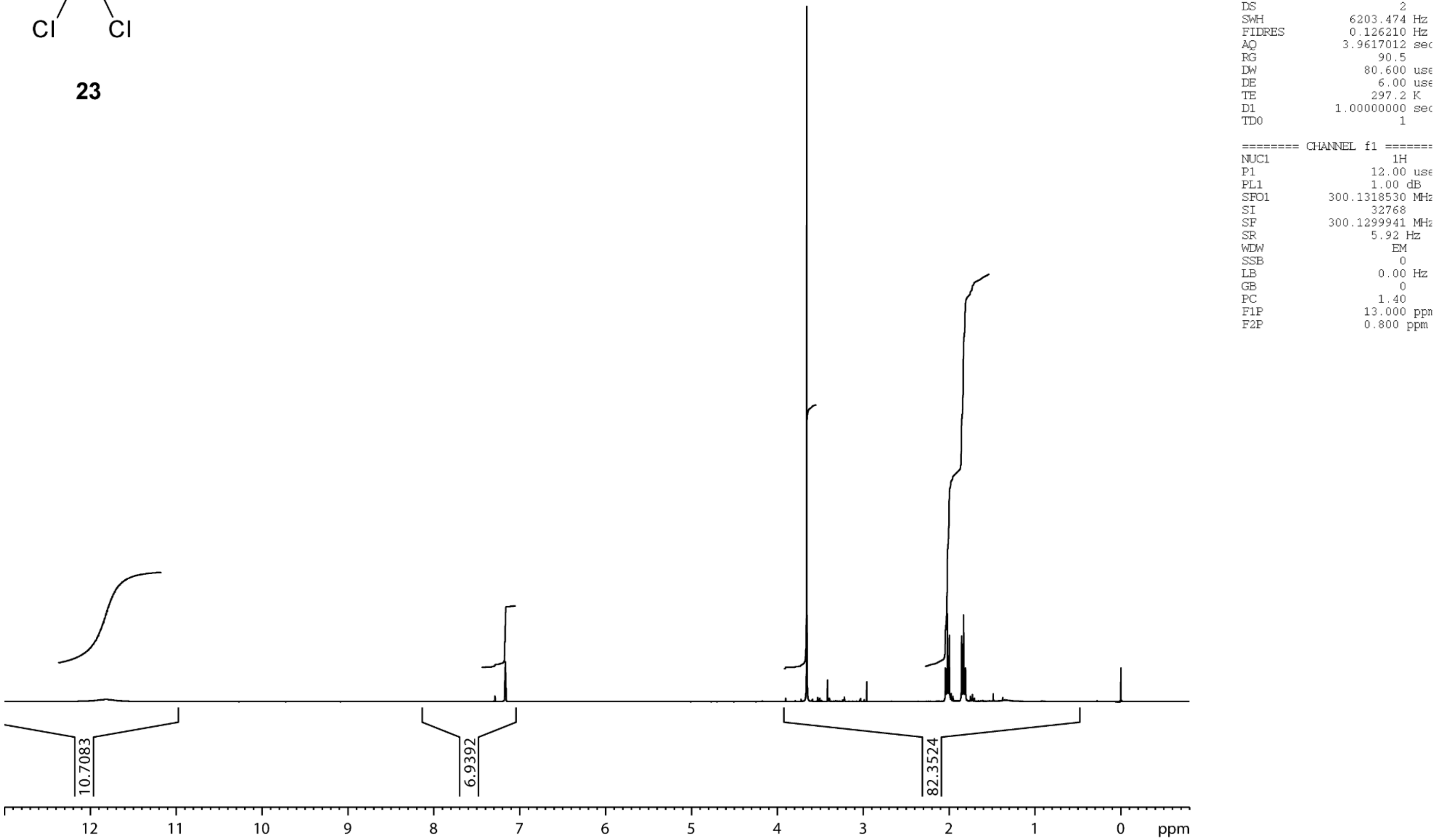
NAME rap140409170619_oc
EXPNO 1
PROCNO 1
Date_ 20140409
Time 17.33
INSTRUM av300
PROBHD 5 mm PABBO BB
PULPROG zg30
TD 49152
SOLVENT C6D6
NS 32
DS 2
SWH 6203.474 Hz
FIDRES 0.126210 Hz
AQ 3.9617012 sec
RG 90.5
DW 80.600 use
DE 6.00 use
TE 297.2 K
D1 1.00000000 sec
TD0 1
======== CHANNEL f1 =======
NUC1 1H
P1 12.00 use
PL1 1.00 dB
SFO1 300.1318530 MHz
SI 32768
SF 300.1299941 MHz
SR 5.92 Hz
WDW EM
SSB 0
LB 0.00 Hz
GB 0
PC 1.40
F1P 13.000 ppm
F2P 0.800 ppm
10.7083
6.9392
82.3524
12
11
10
9
8
7
6
5
4
3
2
1
0
ppm

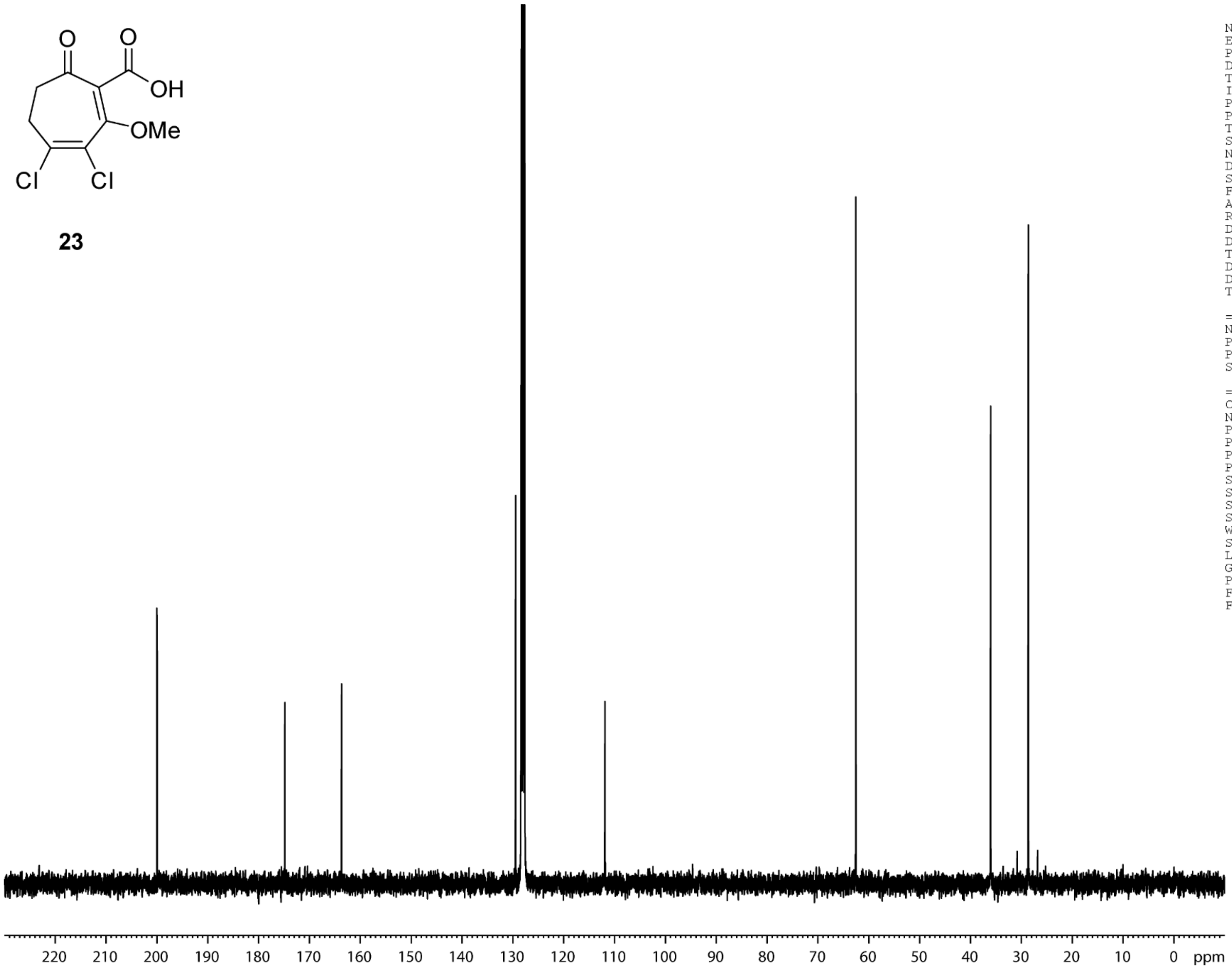

NAME	rap140409170619_oc
EXPNO	2
PROCNO	1
Date_	20140409
Time	17.48
INSTRUM	av300
PROBHD	5 mm PABBO BB
PULPROG	zgpg30
TD	98304
SOLVENT	C6D6
NS	152
DS	4
SWH	19736.842 Hz
FIDRES	0.200774 Hz
AQ	2.4904180 sec
RG	2050
DW	25.333 use
DE	6.00 use
TE	298.2 K
D1	2.00000000 sec
D11	0.03000000 sec
TD0	1

======== CHANNEL f1 =======

NUC1	13C
P1	9.60 use
PL1	1.00 dB
SFO1	75.4761254 MHz

======== CHANNEL f2 =======

CPDPRG2	waltz16
NUC2	1H
PCPD2	80.00 use
PL2	1.00 dB
PL12	15.98 dB
PL13	16.00 dB
SFO2	300.1312005 MHz
SI	65536
SF	75.4677227 MHz
SR	26.31 Hz
WDW	EM
SSB	0
LB	1.00 Hz
GB	0
PC	1.40
F1P	230.000 ppm
F2P	10.000 ppm

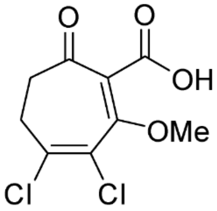

23

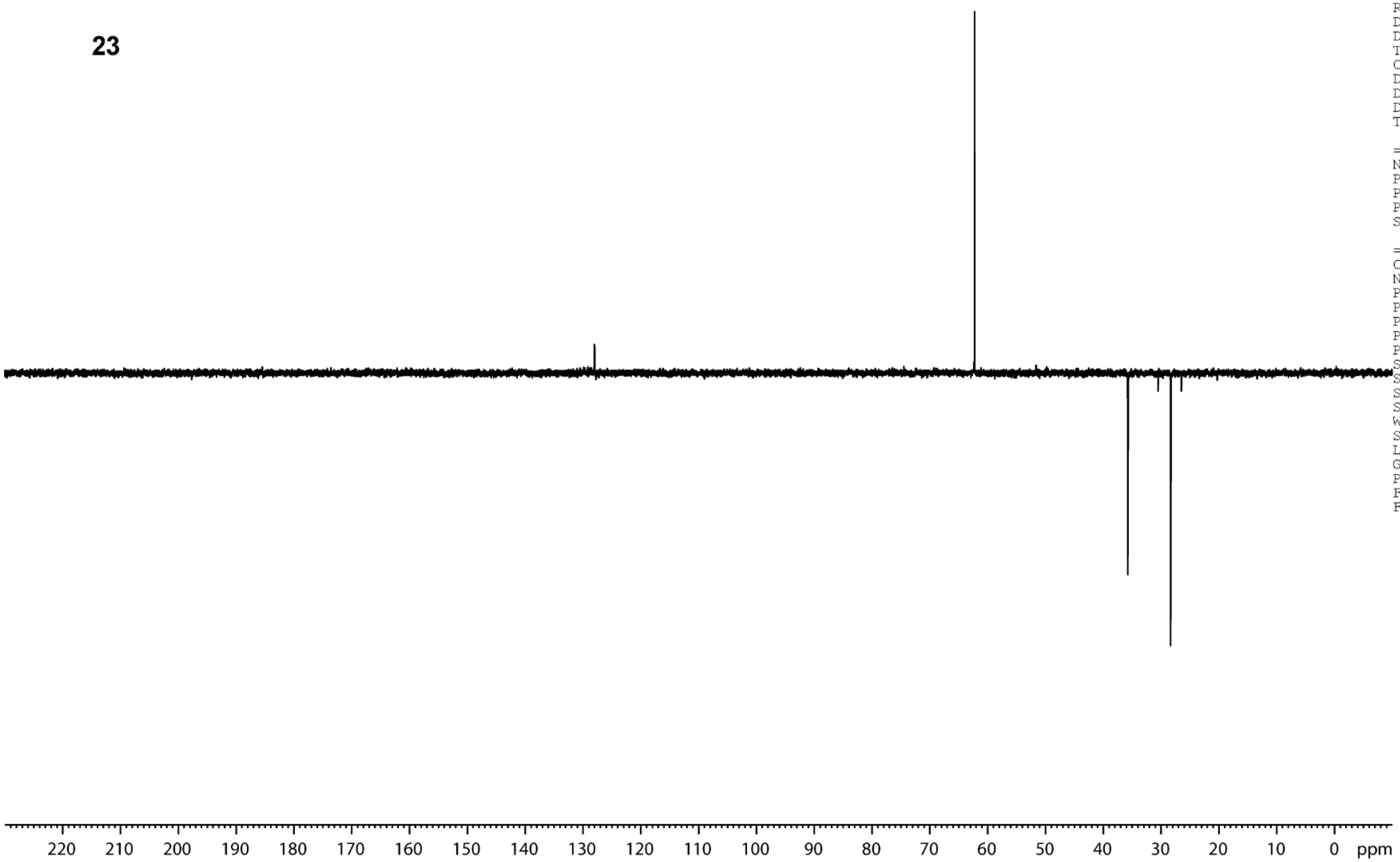


```
NAME       rap140409170619_od
EXPNO                      3
PROCNO                     1
Date_               20140409
Time                   17.56
INSTRUM                av300
PROBHD     5 mm PABBO BB
PULPROG              dept135
TD                     98304
SOLVENT                 C6D6
NS                        76
DS                         4
SWH              19736.842 Hz
FIDRES            0.200774 Hz
AQ               2.4904180 sec
RG                      1030
DW                    25.333 usec
DE                      6.00 usec
TE                     298.2 K
CNST2            145.0000000
D1               2.00000000 sec
D2               0.00344828 sec
D12              0.00002000 sec
TD0                        1

======== CHANNEL f1 ========
NUC1                     13C
P1                      9.60 usec
P2                     19.20 usec
PL1                     1.00 dB
SFO1             75.4761254 MHz

======== CHANNEL f2 ========
CPDPRG2              waltz16
NUC2                      1H
P3                     11.60 usec
P4                     23.20 usec
PCPD2                  80.00 usec
PL2                     1.00 dB
PL12                   15.98 dB
SFO2             300.1312005 MHz
SI                     65536
SF               75.4677450 MHz
SR                      4.03 Hz
WDW                       EM
SSB                        0
LB                      1.00 Hz
GB                         0
PC                      1.40
F1P                  230.000 ppm
F2P                   10.000 ppm
```
